# Cooperation is not rewarded by friendship, but generous and selfish students repel each other in social networks

**DOI:** 10.1371/journal.pone.0326564

**Published:** 2025-06-25

**Authors:** Flóra Samu, Simone Piras, Simone Righi, Marco Setti, Károly Takács

**Affiliations:** 1 CSS-RECENS, HUN-REN Centre for Social Sciences, Budapest, Hungary; 2 Social, Economic and Geographical Sciences, The James Hutton Institute, Aberdeen, Scotland, United Kingdom; 3 Department of Economics ‘Marco Biagi’, University of Modena and Reggio Emilia, Modena, Italy; 4 Department of Agricultural and Food Sciences, University of Bologna, Bologna, Italy; 5 Institute for Analytical Sociology, Department of Management and Engineering, Linköping University, Norrköping, Sweden; Teikyo University - Hachioji Campus: Teikyo Daigaku - Hachioji Campus, JAPAN

## Abstract

Humans cooperate across various contexts, despite the individual costs involved. Cooperation and prosocial behavior may persist because these costs are offset by reputation and other social benefits. Specifically, cooperators and prosocial individuals may receive more friendship nominations and be less likely to face exclusion or avoidance. We test whether such beneficial network dynamics are present in a unique dataset of twenty primary school classes in northern Italy. Cooperation and social preferences of 420 students in grades 4 and 5 were measured with incentivized social dilemma games, and the social network of the entire classroom was traced on two subsequent occasions. We modeled the dynamics of friendship and negative ties with Stochastic Actor-Oriented Models, and conducted a meta-analysis of the results. Our key finding is that, while we do not observe evidence of homophily based on social preferences in friendship nominations - and being prosocial does not lead to receiving more friendship nominations, individuals are significantly more likely to direct negative tie nominations toward peers who made different offers in the dictator game. These results suggest that social network dynamics support cooperation not by rewarding prosocial behavior with friendship, but through repulsion between prosocial and selfish students.

## Introduction

Understanding the drivers of human cooperation is a fundamental challenge for a variety of disciplines [1–10]. Cooperation and prosociality could be attributed to a variety of psychological, economic, cognitive, and social determinants [5, 7, 8, 11]. The key social driver of prosocial behavior is arguably the expectation that favors will be reciprocated directly or indirectly [12, 13].

In addition, it has been argued that individuals cooperate because there are social benefits that can be acquired from cooperation and from appearing to be prosocial [14, 15]. Examples of such social benefits are reputation and high status in the community [16–18]. Cooperation makes individuals attractive partners as their individually costly and other-regarding actions signal their ability to care for others and the potential for further cooperation [19]. Prosocial traits, however, are not always readily observable. Individuals learn about each other through the acquaintance process, in repeated interactions with each other and relevant third parties [20]. Accordingly, individuals seek and collect information about the cooperativeness and prosociality of potential partners - interaction, observation [21, 22], and gossip [22–24].

The direct reciprocation of prosocial acts with a stronger relationship signifies interest in repeated interactions in any social context. Mutually beneficial social ties are maintained, while defection might lead to the termination of existing partnerships [25–32]. Among members who did not form a partnership before, link formation could also be associated with prosociality. Proposing new partnerships to prosocial individuals and avoiding individuals with bad reputation would be both beneficial individually and foster cooperation in the community [25–27, 33, 34]. Consequently, social relationships and their dynamics help explain cooperation and prosociality, i.e., the willingness of individuals to endure personal sacrifices that help overcome social dilemmas [25–27, 33–35].

Considerations about the reputation and relational benefits of cooperation and prosociality are expected to have observable consequences for social network dynamics [36]. First, the preference to establish and maintain relations with prosocial partners should be reflected in higher degrees of cooperators in the partnership network [37–39]. Second, if a tie can be terminated unilaterally [33, 40–42], partnerships between prosocials are more likely to persist. These imply a network pattern, where similarly prosocial individuals are sorted into partnerships [27, 33, 43, 44]. Experimental research provides strong evidence of generosity-based network dynamics [25–27, 33, 34, 45]. Some empirical studies that examined the impact of prosociality on real-life networks confirm that prosocials receive more friendship nominations from classmates and friends are similar to each other in their prosociality due to selection [46, 47].

By mapping only positive affections, such as friendship, previous research fails to capture repulsive forces that also shape social networks in a real-world setting [48]. Negative relations are particularly important in the context of prosocial behavior. For this reason, as a novel contribution, we analyze the impact of prosociality and selfishness on the dynamics of negative ties.

Empirical studies of prosociality are typically based on self-reports. They link prosociality with friendship, as it is known to be associated with support, trust, and kindness [49]. With a few exceptions [50], empirical studies are conducted in the school environment. Schools offer the possibility of collecting complete network data, which is extremely valuable when assessing social network dynamics. In the school setting, a relatively stable set of individuals of similar age repeatedly and systematically interact with each other, while developing their own informal norms and hierarchies [46, 47, 51, 52]. Furthermore, social preferences acquired during school years stabilize in adolescence and may last to adulthood [53–55]. Despite the accessibility and relevance of the school context, previous studies have not linked cooperation experiments with monetary incentives with actual social network dynamics. In this paper, we test the theoretical prediction about the preference for prosocial partners among primary school students using experiments with real stakes and self-reported social network dynamics well after the experiments. Our examined developmental context is the start of early adolescence (10 years of age), when both prosocial behavior [56] and peer relations become increasingly important [57], and social networks gain relevance both in positive [58] and negative dimensions [59].

Although the potential compensation by friendship for cooperation can motivate prosocial acts, we also need to argue why others nominate cooperators and prosocial individuals as friends. First, cooperative and prosocial acts are directly beneficial for interaction partners, and friends are the primary beneficiaries. Cooperation repeatedly occurs in everyday interactions, which makes prosocials desired people to hang out with. Second, a friendship nomination is likely to be reciprocated or exchanged with offers of help and advice [60–63]. Third, prosociality is beneficial to the community and therefore provides reputational and status benefits in the classroom. The formation of a friendship with prosocial individuals with a high reputation and status could bring indirect status benefits [64, 65].

Concerning the opposite end of behaviors and traits, defectors and selfish individuals will likely be avoided and disliked. Defection and selfish behavior has been shown to evoke negative emotions. Defectors are punished voluntarily, even if punishment applies costs to the punisher [66, 67]. Hence, we could expect a higher probability of negative ties toward selfish individuals. Therefore, we hypothesize the following:

**H1 Cooperators and prosocial students are more likely to receive friendship nominations, while defectors and selfish students are more likely to receive negative ties.**

As a result of popularity, prosocial individuals might be overwhelmed by friendship requests. As friendship nominations are largely reciprocal in nature [68], they would not be able to reciprocate all incoming nominations. Due to their own interest, they themselves will be more likely to approve and reciprocate requests from other prosocial individuals. In addition, their own friendship initiations would more likely be toward prosocial individuals. In addition, similar people are likely to be attracted to each other and prosociality might be a relevant dimension of homophily [34, 69, 70]. The tendency of prosocial individuals to befriend each other has been confirmed in both cross-sectional [71] and longitudinal studies [47, 52]. Based on their similarities, selfish individuals may also have a homophilous tendency to form friendship ties with each other. They could also be paired with each other just because they are left out from better opportunities [72–74]. These arguments would imply a stronger tendency of friendship formation among prosocial individuals and an exclusion of selfish classmates to relatively peripheric positions in the friendship network [44]. Similarly, negative ties are expected to occur less likely between similar individuals and form more likely towards defectors and selfish individuals.

In many dimensions, sending a negative tie is a way of informal punishment that might involve certain costs to the sender. The costly punishment of defectors is often characterized as a second-order social dilemma [75]. The dilemma is to bear the cost of punishment for the benefit of other members of the group because punishment can deter selfish behavior [76]. Previous studies show that prosocial individuals are more likely to engage in punishment of non-cooperators [66, 77, 78]. We will be able to test the presence of a tendency to send more negative ties towards selfish individuals and whether such a tendency is more pronounced for prosocial individuals.

As a consequence, we expect to observe assortative mixing by social preferences in the friendship network and disassortative mixing in the network of negative ties. First, as a result of the sorting, prosocials would be likely to befriend with other prosocials, and selfish students would have selfish friends. We also expect that the homophilous selection of friendship will be stronger among prosocials than among selfish students. Consequently, we test whether there is homophily in friendship nominations based on cooperation and prosociality.

**H2 Students, especially cooperative and prosocial students, are more likely to create and maintain friendship relations with similarly cooperative and prosocial others. Negative ties are more likely to occur between individuals with different social preferences and are expected to be directed towards defectors and selfish individuals in particular.**

To contribute to the literature on what compensates for and explains cooperation, the aim of this study is to test the impact of cooperation and prosociality on friendship formation in primary schools. For this purpose, we combine the strengths of previous research that advanced behavioral measures and network analysis. We employ network evolution models to assess how friendship formation is affected by individuals’ social preferences elicited through three incentivized experimental tasks. In addition to the methodological innovations, our dataset also contains data on negative relationships, which allows us to shed a unique light on the role of social preferences in their formation. In addition, we are able to document processes that segregate individuals with different social preferences.

## Data and methods

### Participants

Data were collected in nine primary schools in northern Italy among students in grades 4 and 5, with a median age of 10 years [79]. The classes involved in the study were selected in agreement with the Centre for Education of Sustainability (CEAS) and the Emilia-Romagna Regions in September 2017. A total of 420 students from 20 classes participated in at least one step of the research, but only 405 are retained for analysis (49.1% women). We sought parental consent and received it for all students in all classes, except one, who, for this reason, did not participate in the study. We surveyed the social networks of the classes twice, in the first and third waves of data collection. Data collection for the first wave (including experimental sessions) was conducted between 05/11/2017 and 13/12/2017. Data collection for the second wave (including a lesson given by CEAS, not used in this study) took place between 15/01/2018 and 25/01/2018. Finally, data collection for the last wave (questionnaire only, administered simultaneously in all classes) was carried out on 14/05/2018. Student social preferences were measured in the first wave through incentivized economic games. Data collection for the games was performed on tablets or netbooks provided by the Reggio Emilia Behavioral and Experimental Laboratory (REBEL). The games were programmed using the web-based platform oTree [80]. The questionnaires were filled out on paper with the support of the research team and the teachers.

### Ethics statement

All participants gave informed written consent before participation. The study and its ethics have been approved as part of the project “Food waste and students’ food behaviours”, funded by the Emilia-Romagna Region, Italy, by the University of Modena and Reggio Emilia, Modena. The experimental protocol and consent forms have been approved by the Experimental mobile Laboratory of the University of Bologna (Department of Agricultural and Food Sciences), and the Reggio Emilia Behavioral and Experimental mobile Laboratory (REBEL) of the University of Modena and Reggio Emilia. Ethics approval was issued on 6 September 2017.

### Measurements

#### Social preferences.

We use incentivized economic games to measure cooperation and social preferences rather than vignettes to measure attitudes. In this way, we intend to overcome the complexities in the relationship between social value orientations and cooperation [13, 81]. During the games, participants collected tokens, and depending on the number of tokens collected, they were rewarded with zero to two cinema tickets. In addition, they received a pen of the University of Bologna as a show-up fee. In the end, the computer ranked participants in each class by the number of tokens earned. Students in the top tercile received two cinema tickets, those in the second tercile one. The children played four tasks, but the payoffs for each of them, as well as the total one, were displayed only at the end of the full session. On the last oTree page, students saw how they had performed in the games and to which third of the rankings they belonged to. Rewards were handed over privately and we also protected the anonymity of participants by giving them identification numbers. We chose to implement three standard games to measure cooperation and prosociality, with the objective of keeping the design simple and accessible to children of 10 years of age. The selected games are widely used in experimental economics and social dilemma research to measure cooperation and prosocial behavior. Groups were reshuffled between the games. In all cases, groups were anonymous, with students knowing only they would play with someone else in their class.

The first game, the Public Goods Game (PGG) [82], is a social dilemma game used to measure cooperation [83]. The PGG was played in groups of four. Each participant received 40 tokens and then decided how much to contribute to a group account – 0, 10, 20, 30 or 40 tokens – knowing that twice the total contribution would be divided equally among the four participants. This game aims at measuring the individual willingness to contribute to the creation of a public good that benefits everyone in the group.

The second game was the Dictator Game (DG) [84], which is typically used as a measure of generosity [83]. Students received 100 tokens and could donate any discrete amount between 0 and 100 to a classmate with whom they were randomly and anonymously paired. Everybody played both the roles of the donor and of the recipient, but at the end of the session the computer selected only half of the players to be the donors, and both their and their peers’ payoff was defined by their decision. Dictator game choices measure pure prosociality, i.e., the willingness to pay a cost for the benefit of another individual.

The third game was the Trust Game [85]. In the Trust Game, students played in two roles. Trustors received 100 tokens and had to decide how much to give (TG = Trust Game investment) to their partner, knowing that their partner would receive three times the amount donated and could give back (TGB = Trust Game Back returns) any amount from the obtained tokens (including zero). Participants made decisions in both roles and the computer decided randomly which decisions would be payoff-relevant. Participants were made aware of this process, which was explained with examples. This game was played after the PGG and the DG, to ensure that the received amount would not affect further decisions unintendedly. Since all participants played these games in the same order, previous decisions certainly influence subsequent choices; however, the absence of feedback between tasks reduces the scope for past decisions’ outcomes to influence future decisions. All tasks included 2-4 test questions to assess whether participants understood the game. All questions were answered, and further doubts raised by the participants were addressed before taking the payoff-relevant decisions. Further, all tasks included in the instructions examples and vignettes to enhance understanding.

#### Social relations.

A snapshot of a class network contains essential information about the social processes taking place in the classroom. We collected social network data by asking students to evaluate their peers in the classroom along various dimensions in two waves. Longitudinal data allow us to measure how the relationships in the classroom change over time. In the following, we perform two analyses to examine both positive and negative ties as dependent variables. To study how friendship changes, participants were asked to rate how much they consider their classmates as friends. Classmates’ names were listed in the questionnaire and next to each name, respondents could select a response on a 5-point scale: 1 - good friend, 2 - friend, 3 - indifferent, 4 - not friend, 5 - absolutely not friend. For the analysis, we restrict our attention to good friend nominations. If someone nominated another classmate as good friend, that tie is set to 1, and everything else becomes 0. Based upon these nominations, we constructed one network for each of the classes, where nodes represent children and links between them represent good friend nominations.

In addition to examining friendship dynamics, we investigate how negative relationships develop. To assess the characteristics of negative relations, we similarly constructed one networks for each class, where links between them represent negative ties. Due to the sensitivity of negative nominations, these are naturally sparser. For this reason, we constructed a measurement combining multiple network items. A negative tie exists if at least one negative nomination was sent in the following three questions. First, from the friendship network, not friend and absolutely not friend nominations were treated as negative ties. Second, students were also asked how nice they think others are on a 5-point scale. The two values at the negative end of this scale, namely unpleasant and very unpleasant, were classified as negative. The third question used to build the negative networks asked about the desired desk mates. A negative tie is considered to exist if children nominated a peer as someone, they ‘would not sit next to’, the other options being ‘would sit next to’. In sum, a tie is set to 1 if at least one of five potential nominations (not friend, absolutely not friend, unpleasant, very unpleasant, would not sit next to them) is present, 0 if none of them exist.

**Fig 1:**
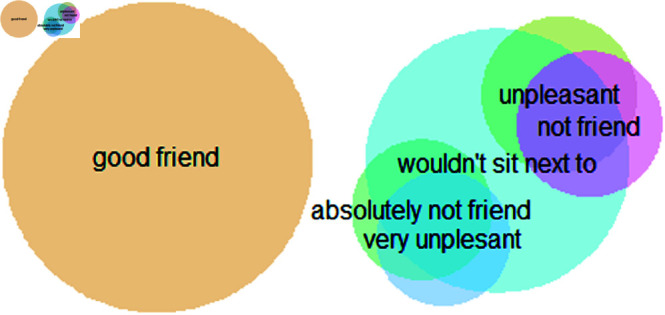
The overlap between the network questions.

The composite negative networks constructed in this way represent avoidance (would not sit) to a greater extent than strong emotions that have been reported less frequently (see overlaps in Table 1). The other four negative nominations (unpleasant, very unpleasant, not friend, absolutely not friend) overlap only 21-29% with the composite network (Fig 1). Given that nominations might not be reciprocated, both positive and negative networks are directed networks, where the fact that *i* is connected to *j* does not imply that *j* is linked to *i*.

**Table 1:** Network descriptives.

	Stability	Reciprocity	Average outdegree	Relative degree	Overlap negative tie
good friend	0.46	0.69	5.74	0.27	0.00
unpleasant	0.12	0.88	1.31	0.06	0.29
very unpleasant	0.18	0.92	0.99	0.05	0.21
not friend	0.11	0.89	1.24	0.06	0.26
absolutely not friend	0.24	0.92	1.05	0.05	0.22
would not sit	0.35	0.74	3.90	0.18	0.84
negative tie	0.38	0.72	5.02	0.25	1.00

### Controls

In both analyses of positive and negative relations, we control for dynamics determined by gender and school performance. We include gender because it has a well-known positive effect on giving in the DG [86, 87], and at the same time children prefer to select same-gender friends [48, 69]. In the data, boys are coded as 0 and girls as 1.

Similarly, school performance may also correlate positively with social preferences [53] and network dynamics [88]. Disregarding these factors could lead to confounding and we might overestimate the preference for prosocial individuals. Here, school performance is measured as the average score in the last tests in Italian language and mathematics, with a range between 0 and 10.

In addition to gender and academic achievement, popularity-based homophily was also revealed in previous studies [89, 90]. Although popularity is expected to be positively related to prosocial preferences, it has a distinct effect on the development of friendship relations [52]. If we do not control for the assortative bonding of popular kids, we may overestimate the impact of social preferences, if popularity and prosociality correlate [52]. In our data, popularity is measured by a network question, where students were asked to nominate those who they think are ‘liked by everyone’ or ‘liked by many others’ in the class. For each child, we divided all incoming nominations by the size of the class (excluding children that were absent on the day of the survey) and after a transformation of this ratio to discrete numbers between 0 and 10, we included it as a node attribute into our models.

### Analytical approach

Our goal is to find evidence for the attractive and repulsive dynamics that shape social networks. In line with our hypotheses, we expect prosocial individuals are more likely to be nominated as good friend and less likely to receive negative ties (H1). Moreover, it is expected that students make friends with similarly prosocial peers, and maintain negative relations with dissimilar others (H2). To test these hypotheses, we apply the standard statistical methodology used to study network dynamics in small, closed groups, called Stochastic Actor-Oriented Models (SAOMs) [91]. This method enables us to test theories about social mechanisms influencing the formation, persistence, and termination of relationships. To estimate SAOMs, the R package RSiena has been developed and constantly maintained by network researchers [92]. SAOMs model the evolution of social ties over time by simulating mechanisms that may be responsible for changes between the empirically observed snapshots of the networks. At each step of the simulations, an actor - chosen at random - can create, maintain, or terminate a tie as a result of the state of the network in a given step of the simulation, which are sequentially modeled [92]. Individual characteristics such as gender or social preferences are also used to explain network changes. The effects of network structures and individual or dyadic attributes on tie formation are expressed as log odds of tie creation or maintenance.

Several well-known network effects can be assessed during the estimation process, such as the general tendency of sending positive or negative social ties (*outdegree*), their bilateral nature (*reciprocity*), the closure of triadic relations (*transitivity*), or even higher-level structural effects. We listed the specifications of the effects in our models in S1 File. After controlling for these structural effects, we are able to test the hypothesized effects of prosociality on tie creation or maintenance.

The decisions in our economic games represent node level characteristics that may affect tie creation. Since decisions in the games are correlated, we run separate models for each game. Effects related to our main hypotheses are specified in a similar way for each model, using the following three effect specifications. First, we surmise that prosocial behavior can influence the tendency of receiving a friendship or a negative tie. The *altX* effect incorporates whether individuals nominate cooperative or prosocial classmates as friends with higher probability and send fewer negative ties to them (H1). This effect is formalized as a preference for forming a tie by actor (*i*) to receivers (*j*) with prosocial behavior (*v*): $\sum_{j}x_{ij}v_{j}$.

Second, we expect a dyadic effect based on the similarity or dissimilarity between the sender and the receiver. To assess whether individuals tend to befriend or avoid negative ties with similarly cooperative or prosocial others (H2) we use an effect called *simX*, which is the sum of centered (for each class) similarity scores between *i* and *j*: $\sum_{j}x_{ij}(sim_{ij}^{v}-\hat{sim^{v}})$, where similarity scores are adjusted to the maximum distance in prosociality $sim_{ij}^{v}=\frac{\max_{ij}|v_{i}-v_{j}|-|v_{i}-v_{j}|}{\max_{ij}|v_{i}-v_{j}|}$ and $\hat{sim^{v}}$ is the mean score. High values indicate nominations between similarly prosocial peers either in the friendship or in the negative network and low values refer to dissimilar relations.

In addition, we expect a differential impact of prosociality on sending negative ties to selfish classmates and sorting into homophilous friendship relations. To test these, we use an interaction effect between the above mentioned *simX* effect and *egoX* which measures the tendency to send more friendship or negative ties by prosocial individuals. Using mathematical terms, *egoX* is the sender’s out-degree weighted by sender’s prosociality: $v_{i}x_{i.}$. The interaction of these two effects allows us to assess significant differences in similarity-based tie formation along the sender’s prosociality. If prosocials are more likely to nominate similar others in the friendship network, and less likely to do so in the negative network compared to selfish children, the interaction of *simXegoX* effect will be significant positive and negative, respectively.

Using these effects, we run 2x4 models for changes in the two networks (friendship and negative) based on the four decisions made in the three economic games (PGG, DG, TG, TGB) and we run each model with and without the interaction term between the activity of prosocial students (*egoX*) and homophily between children with similar social preferences (*simX*). We transformed all decisions in the games into a reduced scale between 0 and 10 (0 and 4 in PGG) by dividing the original values by 10, because RSiena estimations are scale-dependent ([92] p. 27). The detailed results of all SAOM analyses for each of the 20 classes in our sample are reported in S1 File. To summarize the results, we apply meta-analysis which we implement afterwards to the estimates produced by the separate SAOMs. We assume that the collected networks are a sample from the population of networks, and we infer the mean population value for each effect using the restricted maximum-likelihood estimator from the ‘metafor’ R package [93]. It is possible to use different model specification if needed, because in the meta-analysis each effect is tested independently from other effects in the model. It can be traced which effects were included during the estimation process in the detailed results of the meta-analyses reported in S1 File (see column N in the tables).

In the following, before we discuss the results of the meta-analysis, we provide descriptive statistics on the decisions in the games, and we give a general overview of friendship and negative networks.

## Results

### Descriptive results

#### Social preferences.

**Fig 2:**
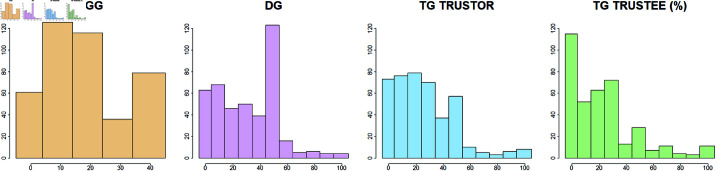
Distribution of allocations in the four economic game decisions. PGG = Public Goods Game, DG = Dictator Game, TG = Trust Game investment, TGB = Trust Game Back returns. The bar charts indicate the sum of choices by available alternatives on the horizontal axis rounded by 10 for DG, TG, and TGB (%).

In the PGG, the average contribution was 18.76 tokens, with a standard deviation of 13.18. 14.53% contributed with 0 tokens (Fig 2). In the DG, 32.45% of the children donated at least half the amount and 9.93% gave nothing to the other player. The average offer was 30.53 tokens, with a standard deviation of 21.71. In the first step of the TG, 25.76 tokens were sent to trustees on average (SD = 21.52). 46 students (11.36%) did not receive anything and therefore they could not decide in the second step, where children returned an average of 22.30% of their available amount to the trustor (SD = 23.27). 19.57% did not return anything.

Correlation is strong between DG and TG decisions ($\rho_{DG,TG}=0.61$, $\rho_{DG,TG}=0.43$, $\rho_{TG,TGB}=0.47,p<0.001$), and we only see moderate correlation between the PGG and the other two games ($\rho_{PGG,DG}=0.34$, $\rho_{PGG,TG}=0.28$, $\rho_{PGG,TGB}=0.19,p<0.001$). The moderate correlation confirms previous research that has differentiated between measuring cooperation with the PGG and generosity with the DG [83].

#### Friendship ties.

The network of *good friends* contains 2495 ties (out of 8655 possible ties) in the first wave and 2128 (out of 8510) in the second, and while these numbers are close, only half of the nominations remained stable over time. The first column in Table 1 shows the stability of ties measured by the Jaccard coefficient expressing the proportion of stable ties compared to the union of the ties in the two waves ($t_{11}/(t_{01}+t_{10}+t_{11})$). Overall, 69% of the good friend nominations are reciprocated (first row, column 2). An average of 5.74 individuals are nominated as a good friend (first row, column 3), which makes up a dense network where 27% of all the possible ties is a good friend tie, taking into account absent children who could not nominate anyone (first row, column 4).

#### Negative ties.

While friendship ties decreased between the two waves, the total number of negative ties – sent by either of the nodes – increased from 1935 to 2323. Only 38% of these ties are stable over time (see column 1 in Table 1). Overall, 72% of the negative ties are reciprocated (column 2, ibid.). On average, one child has 5.02 negative relationships (column 3, ibid.), and a quarter of all possible pairs in the class have a negative relationship (column 4, ibid.).

### Results of the meta-analysis

We summarize the results of separate SAOMs using a meta-analysis. Key results of the meta-analysis are extracted and reported in Table 2 for friendship ties and in Table 3 for negative relations. The detailed results of the meta-analyses including all structural parameters and controls are reported in S1 File.

**Table 2:** Main results of the meta-analysis for friendship ties.

	Friendship							
	Model 1				Model 2			
effect	est	se	*p*	*Q*_*p*_	est	se	*p*	*Q*_*p*_
altX PGG *(H1)*	0.023	0.028	0.398	0.560	0.018	0.036	0.623	0.465
simX PGG *(H2)*	-0.009	0.116	0.935	0.082	-0.018	0.111	0.872	0.150
egoX x simX PGG *(H2)*					0.049	0.101	0.629	0.546
altX DG *(H1)*	-0.003	0.019	0.885	0.503	0.034	0.031	0.284	0.528
simX DG *(H2)*	-0.139	0.085	0.102	0.839	-0.139	0.091	0.127	0.486
egoX x simX DG *(H2)*					-0.129	0.078	0.098	0.251
altX TG *(H1)*	-0.024	0.024	0.311	0.016	0.012	0.024	0.606	0.359
simX TG *(H2)*	-0.081	0.121	0.505	0.470	0.009	0.112	0.933	0.718
egoX x simX TG *(H2)*					-0.129	0.067	0.054	0.870
altX TGB *(H1)*	0.003	0.019	0.865	0.837	-0.003	0.024	0.911	0.916
simX TGB *(H2)*	-0.009	0.190	0.961	0.213	-0.130	0.215	0.546	0.378
egoX x simX TGB *(H2)*					-0.092	0.105	0.379	0.083

**Table 3:** Main results of the meta-analysis for negative ties.

	Negative tie							
	Model 1				Model 2			
effect	est	se	*p*	*Q*_*p*_	est	se	*p*	*Q*_*p*_
altX PGG *(H1)*	0.002	0.025	0.944	0.493	-0.037	0.031	0.224	0.927
simX PGG *(H2)*	-0.049	0.110	0.655	0.193	-0.096	0.113	0.395	0.158
egoX x simX PGG *(H2)*					0.238	0.095	0.012	0.362
altX DG *(H1)*	-0.007	0.017	0.688	0.325	-0.007	0.025	0.768	0.835
simX DG *(H2)*	-0.201	0.083	0.015	0.118	-0.204	0.086	0.018	0.069
egoX x simX DG *(H2)*					0.018	0.083	0.828	0.093
altX TG *(H1)*	-0.01	0.02	0.71	0.02	0.02	0.02	0.48	0.40
simX TG *(H2)*	-0.16	0.11	0.13	0.30	-0.13	0.12	0.27	0.40
egoX x simX TG *(H2)*					-0.09	0.06	0.14	0.38
altX TGB *(H1)*	-0.00	0.02	0.84	0.89	-0.01	0.02	0.43	0.84
simX TGB *(H2)*	-0.21	0.21	0.34	0.03	-0.30	0.22	0.17	0.05
egoX x simX TGB *(H2)*					0.04	0.07	0.58	0.41

Our first hypothesis predicts that prosocial individuals will be popular and will be nominated more likely as friends (H1). More precisely, we tested whether a student with higher contribution in the four economic games is more likely to be nominated as a good friend by their peers. The estimated *altX* effects and the corresponding p-values in Model 1 in Table 2 demonstrate a lack of evidence for generosity-based partner selection. The PGG *altX* parameter does not differ significantly from zero. The results are consistent across the classrooms, as no significant positive *altX* effect have been found in any of the classrooms except one. Results are qualitatively the same for the other games: the null-hypothesis, namely that the effect is null, cannot be rejected. Except one or two classrooms, the result is consistent across all classrooms concerning all games. Hence, we do not find support for friendship formation to prosocial alters expressed in our hypothesis H1.

We do not find evidence for the second hypothesis, in which we expected assortative matching between peers with similar levels of prosociality and protective friendship formation among prosocial individuals (H2). Neither *simX* (Model 1 in Table 2) nor the interaction between *egoX* and *simX* are significantly different from zero (Model 2 in Table 2). The results are consistent across all classrooms for all games with few exceptions only.

Next to these non-significant results, robust effects are found for well-known structural effects (cf. [69, 88, 91, 92]). Friendship dynamics is consistently driven by reciprocity and transitivity, by the preference for same-sex friends, and by forming ties to popular classmates. We also find that popular individuals nominate each other as good friends with a higher probability.

The meta-analysis of negative network dynamics finds non-significant results with regard to sending negative ties toward selfish others (H1). Lower contributions in the PGG do not increase the likelihood of receiving negative nominations within the class (see *altX* PGG in Model 1 in Table 3). Similarly, selfish offers in the DG do not increase the probability of incoming negative ties (*altX* DG ibid.). The same applies to the two decisions in the TG (*altX* TG, *altX* TGB, ibid.).

We found evidence in support of our hypothesis about the dissimilarity effect in generosity in the negative network (H2). Considering DG offers, we found a significant similarity effect (*simX* DG, Model 1 in Table 3), which does not depend on the generosity of the sender (see non-significant effect of *egoX* x *simX* interaction in Model 2). This means that the probability of having negative relationships decreases as the similarity in individuals’ DG offer increases (${\hat{\mu }}_{DG}=-0.201,p<0.05$). The test for heterogeneity does not show significant heterogeneity of this effect (*Q*_*p*_>0.05). This similarity effect has not been found in the TG (*simX* TG, *simX* TGB in Model 1 in Table 3).

Our results contradict the expectation of an elevated impact of generosity on dissimilarity-driven negative ties. We hypothesized that prosocial individuals show higher willingness to punish selfish peers via the creation and maintenance of negative ties. A negative but non-significant effect of similarity indicates no average tendency of sending negative ties to individuals with dissimilar PGG investments. There is a significant interaction effect with cooperativeness in the PGG in Model 2 (*egoX* x *simX*). The significant positive interaction effect shows that cooperative students are more likely to send and maintain negative ties to classmates with high contribution in the PGG. The significant positive effect shows that prosocial children are more likely to nominate other prosocial peers in the negative network (${\hat{\mu }}_{PGG}=0.238,p<0.05$). The interaction of the sender’s investments and the similarity between the sender and the receiver of a negative tie in the other three models fail to repeat this result (see *egoX* x *simX* effects in Model 2). The heterogeneity test [93] suggests that this effect is homogeneous in the population of networks (*Q*_*p*_>0.05), which strengthens the validity of the result.

Concerning other structural effects and controls in models of negative networks, we found a robust positive effect of reciprocity, indicating that negative nominations are reciprocated, and a negative effect of transitivity, both are in line with structural balance considerations [20, 48]. Furthermore, we consistently found a positive effect of indegree-popularity, meaning that those individuals who receive more negative nominations are more likely to receive further ones. We found a robust positive effect of outdegree-activity and a negative effect of gender homophily for negative ties. In addition, we consistently found a negative effect of popularity, indicating that popular peers are less avoided. We also found that popular individuals form negative relationships less likely with each other. These structural effects are consistent considering all four games.

### Goodness of fit

We give an overview of how well our models perform. The goodness of fit (GOF) of SAOMs can be assessed by contrasting the empirical networks with simulated ones using the parameter estimations from the models. Global characteristics are the bases for comparison that are not fitted by the parameters in the model unequivocally; thus, they can provide information on how well a model represents the empirical data. We examine the distributions of indegree, outdegree, and geodesic distance (i.e., the length of the shortest path between two individuals); and the triad census (the occurrence of triadic configurations [94, 95]). Dissimilarity between the simulated and empirical data is evaluated using p-values from a Mahalanobis’ distance measure, where a low number indicates a poor fit ([92] p. 59). Summaries of goodness of fit statistics are reported in S1 File. It is natural that models do not fit perfectly in every classroom. As conventional, due to differences in model fit, some classroom models have been slightly adjusted. We highlight classes with inadequate model fit only in S1 File. In the following, we discuss classes with poorer model fit.

Models of friendship dynamics in general have a better model fit than models of negative ties, since fewer classes have p-values below 0.05. While model variations were hardly ever necessary for modeling friendship dynamics, this has more often been the case for modeling negative ties. Beyond the conventional indegree-popularity and outdegree-activity effects, an indegree-activity effect has been included for five classrooms for PGG and DG models, for four classrooms in TG and TGB models, and a truncated outdegree effect has been included for four classrooms in all models to improve model fit. After the slight modifications, model fits improved. Please note that violin plots are reported only for classrooms with inadequate fit in the final model specifications.

First, we evaluate indegree distributions, where we check whether the observed frequencies by the number of incoming nominations fit well to the simulated values. Indegree distribution fails to fit the negative network in two classes. The number of individuals without any friendship nomination is close to the confidence intervals of the simulated values in class 7 and individuals with a maximum indegree of five are higher than the upper quartile of the simulated values in class 18 (see S1 File).

The outdegree distribution is not increasing gradually with the number of outgoing ties in class 11 in the friendship models (S1 File). The cumulative number of students by outgoing friendship nominations in the second wave shows that the number of individuals without any friendship nomination is higher than expected. We overestimate the number of students with low outdegrees, and since there is a big jump in the empirical network between the outdegree of four and six, we underestimate outdegrees afterwards. The very small p-value suggests that the observed data are far from the simulated data; therefore, we exclude class 11 from the friendship analysis. Our models for negative ties simulate slightly different cumulative outdegree distributions than it is observed in class 6 and 10 (S1 File), but these classes are not excluded from the meta-analysis.

The third measure (triad census) tests the occurrence of 16 triadic configurations. None of the observed values fit badly with the simulated values calculated by our model on friendship and negative dynamics.

The fourth property examined is geodesic distance. The model on negative ties does not fit well two classes (class 6 and 13) regarding the cumulative number of pairs of actors with the shortest path of one, two and three. Due to the small class sizes, the fit of pairs at distance three are poor.

## Discussion

In this study, we examined the role of cooperation and social preferences in shaping social networks dynamics in a primary school environment. We argued that one reason why we observe individually costly cooperation in general among humans is the social compensation cooperators might receive. Such social compensation might take place with the increasing relevance of peer relations in early adolescence. Based on these theoretical arguments, we have expected that cooperators and prosocial individuals would receive more friendship nominations and would be less likely the targets of negative relations among our study population of 10-year-old students. We also argued that friendship to cooperative and prosocial peers might be directly and indirectly beneficial.

Based on these arguments, we formulated hypotheses emphasizing the role of cooperation and social preferences in friendship formation and negative ties among early adolescents. Considering friendship and negative relations within the classroom, we tested the relevance of cooperation and social preferences using social network analysis. By estimating the dynamics of friendship and negative relations in two waves of social network data after the measurement of cooperation and social preferences, we could make valid statistical inference about the long-term social impact of generous decisions in economic games. Unlike other studies [46, 47], we found no evidence of a preference for more generous friends. Moreover, we failed to prove the existence of repulsive forces toward selfish individuals. These results contradict theoretical arguments that friendship would be a major compensation for the costs of cooperation [19, 46] and also those that argue for the relevance of seeking friendship in the expectation of returns. Friendship formation among classmates is not driven by such calculative rationality [96], but by known structural mechanisms such as reciprocity, gender homophily, and indegree popularity. Some of these factors could correlate with prosociality, but the consideration of prosociality of alters and prosociality-based homophily did not add significantly to the explanation of friendship dynamics.

Cooperativeness and prosociality, however, could be prematurely perceived among 10-year-old students, with less associations to monetary and material benefits. In addition, social preferences could be hidden traits and peers might not be aware of others’ social preferences that might explain the lack of their impact in terms of network formation [20] Moreover, defectors and selfish individuals might be motivated to deceive others and act as if they had prosocial attitudes in expectation of future returns [97, 98]. Nevertheless, unobserved preferences can be signaled honestly with cooperation and reputation. Reputation and prosocial behavior are strongly linked: it has been shown empirically that generosity leads to reputational gains [99]. It is therefore conceivable that the effects of prosociality are mediated by perceived popularity, a tendency to form positive ties to classmates, who already perceived to be popular. We see in our models that popular individuals, indeed, attract more friendship and receive less negative nominations. In addition, friendship nominations between popular students are more likely, while negative nominations are less likely (see the results of the meta-analyses). In our case, however, this is more likely to be a genuine popularity effect rather than a mediation of prosociality by perceived popularity. While other studies find a strong correlation between popularity and prosociality [52], there is no correlation in our sample ($\rho_{PGG}=-0.189,p<0.001;\rho_{DG}=-0.013,p=0.773;\rho_{TG}=-0.046,p=0.314;\rho_{TGB}=-0.011,p=0.829$).

Another potential reason for the absence of the impact of cooperation and prosociality on friendship formation is that our research has taken place in classrooms with established social networks. Network dynamics in these classrooms are no longer driven by considerations of prosociality and indirect reciprocity, because students already spent enough time together to gain direct experience about each other. During this time, they have already explored relevant traits of others and incorporated them into their friendship choices. Based on our arguments, however, we should still observe that friendships were formed more likely among peers similar with regard to their prosociality. Therefore, we should have observed that individuals organize themselves into partnerships with similar peers and maintain negative relations with dissimilar others. Our results do not provide evidence for such sorting in the friendship network. This negative result is in line with empirical research that found no evidence of altruism-based homophily in social networks [100].

At the same time, we found evidence that dissimilar individuals are more likely to establish and maintain negative relations. This result supports theories that argue for the importance of repulsion in dynamics of social networks [48, 101] and for the maintenance of cooperation [102]. Moreover, while friendship ties might be driven by other determinants of preferences, dimensions of homophily, and opportunities, negative ties could be closer associated with cooperation. Negative ties between cooperators and defectors signify their potential as a form of informal punishment [103]. We document that differences in generosity increase the probability of negative relationships between students. We found a strong and significant effect considering behavior in the Dictator Game (DG). The DG is better suited to measure generosity and intrinsic social preferences, while other games (PGG, and the first decision in the TG) require more strategic thinking [11]. The second decision of the trust game (TGB) is somewhat closer to the dictator game and shows a similar but non-significant effect. Mutually negative nominations between prosocial and selfish individuals may indicate the combined presence of prosocial and antisocial punishment [104]. A limitation of the study is the compensation of participants with cinema tickets instead of exact monetary payoffs. In addition, participants were ranked and relative payoffs could have created additional incentives for competition and against cooperation. Furthermore, incentivized economic games with such low stakes might have limited external validity and impact on real social processes such as friendship formation. In addition, behavior in our games could have been less reliable than games played with adults and could have been less stable than responses to social value orientation questionnaires [105].

Overall, as a main contribution of the study we highlighted the relevance of cooperation and social preferences for the dynamics of negative relations in early adolescence. Friendship ties, on their own, do not cover all dimensions of social relationships, and the examination of forces acting negative ties is important [48, 106]. One of the limitations of this study is that it does not model these two forces simultaneously. The signs of the studied effects in the two networks (e.g., *simX*) suggest that these dynamics are not just the two sides of the coin but have non-symmetrical and more complex effects. Their joint consideration is very much underrepresented in social network research though it would be necessary to better understand structural, attitudinal, and behavioral dynamics in various social contexts.

## Supporting information

S1 File Supporting information and analysis (PDF)
