# Supplementary material for: Cooperation is not rewarded by friendship, but generous and selfish students repel each other in social networks
Source: PLoS One. 2025 Jun 25;20(6):e0326564. doi: 10.1371/journal.pone.0326564 (PMC12194103; doi:10.1371/journal.pone.0326564)
Supplement: S1 File — (PDF) [file pone.0326564.s001.pdf]

Table 1: Effects

name	figure	equation	description
density		$\sum_j x_{ij}$	constant, the out-degree if all other effects are zero
recip		$\sum_j x_{ij}x_{ji}$	the tendency to reciprocate a tie
transTrip	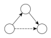	$\sum_{j,h} x_{ij}x_{ih}x_{hj}$	the tendency to close open triads in sender's neighbourhood
transRecTrip	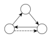	$\sum_{j,h} x_{ij}x_{ji}x_{ih}x_{hj}$	the interaction between the transTrip and recip
outAct	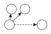	x_{i+}^2	sender's activity based on their outgoing ties
inAct	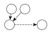	$x_{i+}x_{+i}$	sender's activity based on their incoming ties
inPop	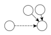	$\sum_j x_{ij}x_{+i}$	the tendency to send a tie based on receiver's incoming ties
outPop	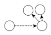	$\sum_j x_{ij}x_{j+}$	the tendency to send a tie based on receiver's outgoing ties
outTrunc	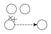	$\min\left(x_{i+}, p\right)$	the tendency to be an isolate based on sender's outgoing ties
egoX		$v_i x_{i+}$	sender's activity based on her value in a given covariate
altX		$\sum_j x_{ij}v_j$	the tendency to send a tie based on receiver's value in a given covariate
sameX		$\sum_j x_{ij}I\{v_i = v_j\}$	the tendency to send a tie to receivers with exactly the same value in a given covariate
egoXaltX		$v_i \sum_j x_{ij}v_j$	the tendency to send a tie to receivers with similar value in a given covariate

simX		$\sum_j x_{ij}(sim^v_{ij} - \widehat{sim^v})$	the tendency to send a tie to receivers with similar value in a given covariate, centered (where $sim^v_{ij} = \frac{\max_{ij}\lvert v_i - v_j\rvert - \lvert v_i - v_j\rvert}{\max_{ij}\lvert v_i - v_j\rvert}$ and $\widehat{sim^v}$ is the mean score)

See more about the effects in [1].

Table 2: Results of the meta-analysis for friendship network and PGG, Model 1

	est	se	N	p	tau2	Q	Qp
outdegree (density)	-1.219	0.2917	20	0.000	0.801	33.791	0.019
reciprocity	1.057	0.1219	20	0.000	0.000	11.539	0.904
transitive triplets	0.174	0.0275	20	0.000	0.001	18.253	0.506
transitive recipr. triplets	-0.043	0.0432	20	0.319	0.064	16.402	0.630
indegree - popularity	-0.024	0.0197	20	0.217	0.000	21.030	0.335
outdegree - popularity	-0.099	0.0204	19	0.000	0.045	22.870	0.196
outdegree - activity	0.025	0.0128	20	0.054	0.034	41.696	0.002
sex alter	0.009	0.0969	20	0.926	0.273	31.901	0.032
sex ego	0.012	0.0753	20	0.872	0.155	23.419	0.219
same sex	0.555	0.0865	20	0.000	0.253	36.779	0.008
grade alter	0.070	0.0430	20	0.103	0.086	22.802	0.246
grade ego	-0.043	0.0414	20	0.299	0.079	23.615	0.211
grade similarity	0.211	0.1492	20	0.158	0.383	30.023	0.052
PGG alter	0.023	0.0276	20	0.398	0.001	17.436	0.560
PGG ego	0.008	0.0385	20	0.828	0.103	33.709	0.020
PGG similarity	-0.009	0.1164	20	0.935	0.239	28.093	0.082
popularity alter	0.185	0.0277	20	0.000	0.048	21.725	0.298
popularity ego	0.013	0.0200	20	0.526	0.026	22.842	0.244
popularity ego x popularity alter	0.024	0.0072	20	0.001	0.012	25.770	0.137

Table 3: Goodness of fit statistics, Friendship & PGG, Model 1

	Indegree distribution	Outdegree distribution	Geodesic Distance	Triad Census
1	0.746	0.535	0.969	0.992
2	0.908	0.095	0.572	0.982
3	0.911	0.641	0.243	0.529
4	0.797	0.428	0.884	0.998
5	0.479	0.070	0.290	0.160
6	0.673	0.773	0.359	0.212
7	0.217	0.698	0.686	0.840
8	0.470	0.707	0.983	0.967
9	0.906	0.933	0.582	0.951
10	0.804	0.623	0.441	0.990
11	0.541	0.004	0.332	0.730
12	0.246	0.726	0.774	0.826
13	0.291	0.271	0.156	0.693
14	0.870	0.786	0.953	0.881
15	0.807	0.982	0.880	0.849
16	0.872	0.442	0.438	0.226
17	0.635	0.804	0.988	0.994
18	0.993	0.531	0.904	0.967
19	0.984	0.694	0.344	0.716
20	0.820	0.712	0.959	0.821

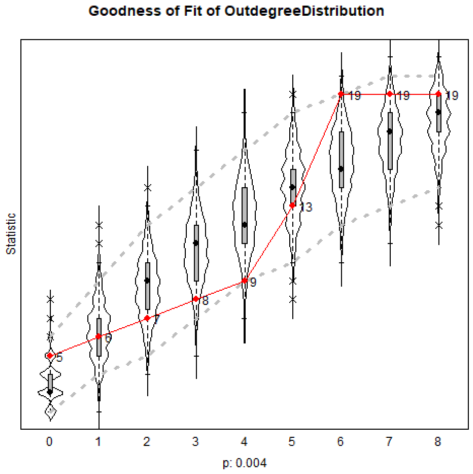

(a) Class 11

Figure 1: Classes with poor fit, Friendship & PGG, Model 1

Table 4: Results of separate SAOMs for friendship network and PGG, Model 1

	1	2	3	4	5	6	7	8	9	10
outdegree (density)	-0.875	0.29	-5.615	1.178	-2.769	-0.253	-0.017	1.474	-1.314	-1.65
	[0.608]	[1.844]	[1.885]	[1.38]	[0.68]	[0.815]	[0.961]	[1.154]	[0.636]	[0.976]
reciprocity	1.151	1.227	1.809	0.588	1.777	0.505	0.671	1.107	1.085	1.889
	[0.384]	[1.063]	[0.909]	[0.61]	[0.537]	[0.429]	[0.641]	[0.403]	[0.366]	[0.726]
transitive triplets	0.213	0.45	0.195	0.029	-0.012	0.111	0.279	0.317	0.19	0.312
	[0.092]	[0.295]	[0.211]	[0.169]	[0.098]	[0.126]	[0.154]	[0.124]	[0.082]	[0.133]
transitive recipr. triplets	0.048	-0.248	-0.276	0.213	0.194	0.031	-0.062	-0.119	-0.114	-0.251
	[0.136]	[0.388]	[0.284]	[0.22]	[0.151]	[0.185]	[0.246]	[0.184]	[0.137]	[0.207]
indegree - popularity	0.018	-0.11	0.12	-0.087	0.049	-0.103	-0.12	-0.332	-0.064	-0.107
	[0.042]	[0.171]	[0.139]	[0.127]	[0.07]	[0.092]	[0.102]	[0.14]	[0.062]	[0.094]
outdegree - popularity	-0.207	-0.205	0.048	-0.134	-0.137	-0.087	-0.131	-0.283	-0.097	-0.14
	[0.071]	[0.236]	[0.099]	[0.162]	[0.05]	[0.055]	[0.106]	[0.083]	[0.038]	[0.051]
outdegree - activity	-0.041	-0.081	0.245	-0.086	0.093	0.022	-0.093	0.003	0.026	0.067
	[0.034]	[0.114]	[0.12]	[0.087]	[0.027]	[0.032]	[0.063]	[0.034]	[0.023]	[0.035]
sex alter	-0.035	0.169	-0.442	-0.601	0.355	0.226	-0.188	-1.062	-0.093	-0.362
	[0.189]	[0.499]	[0.57]	[0.351]	[0.218]	[0.265]	[0.362]	[0.353]	[0.214]	[0.285]
sex ego	-0.15	-0.041	-0.055	-0.23	0.045	-0.096	0.187	0.011	0.499	-0.011
	[0.212]	[0.482]	[0.586]	[0.394]	[0.23]	[0.258]	[0.381]	[0.231]	[0.212]	[0.219]
same sex	0.487	0.773	1.56	-0.063	0.948	0.173	0.842	0.124	0.681	0.9
	[0.179]	[0.561]	[0.614]	[0.276]	[0.232]	[0.17]	[0.275]	[0.265]	[0.196]	[0.332]
grade alter	-0.148	0.215	0.294	0.204	-0.006	-0.075	-0.129	0.457	0.173	0.184
	[0.171]	[0.23]	[0.26]	[0.193]	[0.123]	[0.1]	[0.319]	[0.181]	[0.169]	[0.147]
grade ego	-0.363	-0.296	-0.56	-0.099	-0.084	-0.049	-0.311	-0.017	0.149	0.228
	[0.202]	[0.243]	[0.458]	[0.185]	[0.122]	[0.069]	[0.31]	[0.138]	[0.16]	[0.145]
grade similarity	0.345	0.824	0.188	-0.203	0.268	0.882	0.586	0.856	-0.591	-1.416
	[0.37]	[0.797]	[0.615]	[0.546]	[0.636]	[0.357]	[0.476]	[0.459]	[0.328]	[0.685]
PGG alter	-0.008	1.199	0.402	0.058	0.007	-0.064	0.156	-0.159	0.087	-0.039
	[0.095]	[0.594]	[0.285]	[0.157]	[0.101]	[0.105]	[0.177]	[0.13]	[0.09]	[0.132]
PGG ego	0.253	0.213	-0.099	-0.362	-0.138	0.059	0.111	-0.073	-0.016	-0.312
	[0.151]	[0.424]	[0.256]	[0.216]	[0.094]	[0.092]	[0.196]	[0.111]	[0.086]	[0.12]
PGG similarity	-0.338	-1.513	-0.074	0.325	0.333	-0.717	1.204	-0.28	0.015	-0.817
	[0.438]	[0.895]	[0.839]	[0.535]	[0.354]	[0.351]	[0.56]	[0.477]	[0.285]	[0.486]
popularity alter	0.087	0.267	0.069	0.229	0.235	0.096	0.418	0.378	0.191	0.293
	[0.066]	[0.221]	[0.165]	[0.126]	[0.084]	[0.068]	[0.188]	[0.134]	[0.075]	[0.109]
popularity ego	0.191	-0.092	0.309	-0.008	-0.017	-0.016	0.138	-0.031	0.099	0.017
	[0.086]	[0.203]	[0.211]	[0.11]	[0.06]	[0.048]	[0.166]	[0.06]	[0.053]	[0.079]
popularity ego x popularity alter	0.019	0.292	-0.164	0.059	0.033	0.036	-0.056	0.026	-0.027	0.059
	[0.014]	[0.205]	[0.083]	[0.038]	[0.03]	[0.018]	[0.055]	[0.022]	[0.02]	[0.034]
Overall maximum convergence ratio:	0.156	0.193	0.109	0.195	0.209	0.203	0.207	0.217	0.156	0.193

	11	12	13	14	15	16	17	18	19	20
outdegree (density)	-3.069	-2.333	-1.886	-1.233	-0.009	-0.927	1.304	-0.38	-1.754	-2.83
	[0.822]	[0.79]	[0.691]	[0.696]	[1.169]	[0.624]	[2.771]	[1.7]	[2.119]	[3.196]
reciprocity	0.625	1.575	1.189	1.259	0.758	0.51	1.285	2.011	1.583	1.866
	[0.656]	[0.687]	[0.573]	[0.436]	[0.473]	[0.391]	[0.594]	[1.187]	[0.983]	[1.442]
transitive triplets	0.086	0.101	0.364	0.272	0.206	0.08	0.397	1.315	0.224	-0.176
	[0.145]	[0.08]	[0.176]	[0.138]	[0.074]	[0.083]	[0.196]	[0.615]	[0.29]	[0.436]
transitive recipr. triplets	0.077	-0.088	-0.265	-0.271	-0.158	0.167	0.032	-0.999	-0.114	0.322
	[0.187]	[0.121]	[0.268]	[0.192]	[0.084]	[0.111]	[0.315]	[0.882]	[0.456]	[0.757]
indegree - popularity	-0.087	0.056	0.087	-0.104	-0.244	-0.018	-0.12	-0.026	-0.303	0.362
	[0.123]	[0.073]	[0.084]	[0.11]	[0.146]	[0.05]	[0.256]	[0.148]	[0.276]	[0.243]
outdegree - popularity	-0.021	-0.085	-0.105	-0.022	0.087	-0.125	0.126	0.188	-0.157	0
	[0.077]	[0.046]	[0.113]	[0.092]	[0.061]	[0.041]	[0.865]	[0.564]	[0.14]	[NA]
outdegree - activity	0.108	0.067	-0.003	-0.004	0.012	0.003	-0.526	-0.687	0.195	-0.212
	[0.037]	[0.026]	[0.045]	[0.038]	[0.025]	[0.027]	[0.228]	[0.426]	[0.124]	[0.342]
sex alter	0.549	0.168	0.871	-0.005	-0.434	0.323	0.286	0.098	-0.162	0.423
	[0.354]	[0.228]	[0.351]	[0.241]	[0.306]	[0.217]	[0.61]	[0.546]	[0.885]	[0.831]
sex ego	-0.221	0.409	0.841	0.191	-0.363	0.02	-0.855	-0.358	-1.306	-2.119
	[0.242]	[0.274]	[0.405]	[0.235]	[0.169]	[0.213]	[0.691]	[0.721]	[1.318]	[1.941]
same sex	1.328	0.379	0.248	0.889	0.227	0.427	0.565	0.852	0.318	1.968
	[0.325]	[0.221]	[0.362]	[0.239]	[0.154]	[0.18]	[0.359]	[0.472]	[0.598]	[0.986]
grade alter	-0.149	-0.229	0.032	0.31	-0.104	0.149	0.338	0.038	0.286	0.284
	[0.178]	[0.193]	[0.12]	[0.175]	[0.109]	[0.117]	[0.213]	[0.29]	[0.404]	[0.333]
grade ego	-0.261	0.326	0.028	-0.064	-0.229	0.15	0.197	0.077	0.4	0.3
	[0.155]	[0.229]	[0.157]	[0.121]	[0.095]	[0.135]	[0.29]	[0.412]	[0.437]	[0.565]
grade similarity	1.292	-0.264	0.978	-0.159	0.362	-0.1	-0.369	1.09	-1.567	0.85
	[0.598]	[0.407]	[0.663]	[0.483]	[0.389]	[0.384]	[0.946]	[0.882]	[1.25]	[1.245]
PGG alter	0.048	-0.005	0.04	-0.005	0.36	0.046	0.146	-0.138	0.036	-0.449
	[0.096]	[0.096]	[0.147]	[0.091]	[0.16]	[0.077]	[0.213]	[0.138]	[0.242]	[0.444]
PGG ego	-0.076	0.199	-0.058	0.029	0.173	0.143	0.256	0.084	-0.678	0.697
	[0.073]	[0.138]	[0.163]	[0.09]	[0.075]	[0.07]	[0.276]	[0.174]	[0.36]	[0.86]
PGG similarity	0.411	-0.583	0.522	0.049	-0.047	-0.26	0.922	1.179	0.872	2.114
	[0.379]	[0.401]	[0.658]	[0.324]	[0.293]	[0.31]	[0.617]	[0.734]	[1.055]	[1.852]
popularity alter	0.459	0.199	-0.029	0.132	0.401	0.263	0.168	-0.013	0.402	0.218
	[0.162]	[0.082]	[0.096]	[0.091]	[0.206]	[0.092]	[0.197]	[0.185]	[0.22]	[0.289]
popularity ego	-0.012	0.085	-0.199	0.04	0.051	-0.081	-0.136	-0.285	-0.458	-0.146
	[0.091]	[0.075]	[0.13]	[0.073]	[0.063]	[0.062]	[0.198]	[0.286]	[0.249]	[0.334]
popularity ego x popularity alter	-0.019	0.024	0.08	0.027	0.057	0.025	0.075	-0.004	0.062	0.086
	[0.028]	[0.022]	[0.045]	[0.023]	[0.024]	[0.032]	[0.053]	[0.07]	[0.055]	[0.081]
Overall maximum convergence ratio:	0.219	0.179	0.173	0.182	0.144	0.146	0.176	0.115	0.244	0.116

Table 5: Results of the meta-analysis for friendship network and PGG, Model 2

	est	se	N	p	tau2	Q	Qp
outdegree (density)	-1.247	0.295	20.000	0.000	0.803	33.420	0.021
reciprocity	1.070	0.127	20.000	0.000	0.000	9.857	0.956
transitive triplets	0.178	0.029	20.000	0.000	0.000	14.764	0.737
transitive recipr. triplets	-0.045	0.043	20.000	0.295	0.045	14.077	0.779
indegree - popularity	-0.022	0.020	20.000	0.273	0.002	20.704	0.353
outdegree - popularity	-0.102	0.020	19.000	0.000	0.041	21.945	0.234
outdegree - activity	0.026	0.012	20.000	0.029	0.031	37.279	0.007
sex alter	0.012	0.099	20.000	0.906	0.282	32.188	0.030
sex ego	0.011	0.076	20.000	0.884	0.153	23.363	0.222
same sex	0.566	0.087	20.000	0.000	0.254	36.868	0.008
grade alter	0.072	0.042	20.000	0.089	0.070	21.335	0.319
grade ego	-0.036	0.042	20.000	0.396	0.083	23.531	0.215
grade similarity	0.219	0.148	20.000	0.139	0.379	29.760	0.055
PGG alter	0.018	0.036	20.000	0.623	0.002	18.877	0.465
PGG ego	0.019	0.035	20.000	0.592	0.076	27.212	0.100
PGG similarity	-0.018	0.111	20.000	0.872	0.169	25.313	0.150
popularity alter	0.184	0.029	20.000	0.000	0.051	21.689	0.300
popularity ego	0.009	0.022	20.000	0.668	0.037	24.560	0.176
popularity ego x popularity alter	0.023	0.007	20.000	0.002	0.013	27.587	0.092
int. PGG ego x PGG similarity	0.049	0.101	20.000	0.629	0.000	17.652	0.546

Table 6: Goodness of fit statistics, Friendship & PGG, Model 2

	Indegree distribution	Outdegree distribution	Geodesic Distance	Triad Census
1	0.759	0.686	0.968	0.994
2	0.910	0.077	0.557	0.977
3	0.866	0.747	0.291	0.624
4	0.748	0.402	0.847	0.994
5	0.456	0.038	0.290	0.162
6	0.660	0.759	0.349	0.246
7	0.233	0.671	0.713	0.858
8	0.386	0.768	0.987	0.979
9	0.883	0.931	0.583	0.963
10	0.848	0.553	0.489	0.989
11	0.469	0.002	0.325	0.835
12	0.216	0.666	0.762	0.822
13	0.226	0.328	0.144	0.652
14	0.846	0.810	0.947	0.893
15	0.804	0.971	0.871	0.853
16	0.878	0.468	0.397	0.241
17	0.612	0.814	0.994	0.993
18	0.991	0.514	0.926	0.965
19	0.985	0.697	0.352	0.673
20	0.743	0.649	0.946	0.766

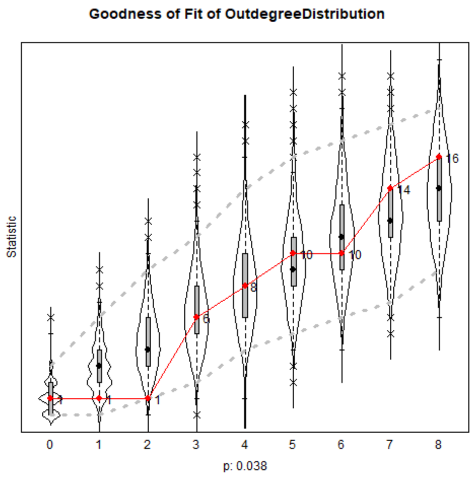

(a) Class 5

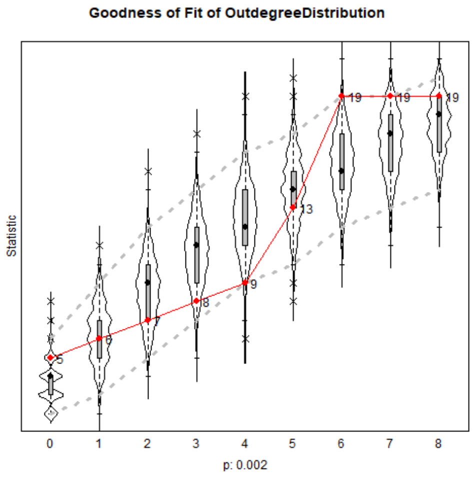

(b) Class 11

Figure 2: Classes with poor fit, Friendship & PGG, Model 2

Table 7: Results of separate SAOMs for friendship network and PGG, Model 2

	1	2	3	4	5	6	7	8	9	10
outdegree (density)	-0.94	0.466	-5.541	1.112	-2.828	-0.252	-0.078	1.487	-1.289	-1.654
	[0.628]	[1.878]	[1.816]	[1.44]	[0.806]	[0.886]	[0.902]	[1.181]	[0.642]	[0.942]
reciprocity	1.158	1.136	1.727	0.584	1.779	0.503	0.642	1.086	1.044	1.888
	[0.385]	[1.016]	[0.873]	[0.619]	[0.576]	[0.444]	[0.696]	[0.397]	[0.41]	[0.787]
transitive triplets	0.219	0.428	0.197	0.028	-0.019	0.106	0.266	0.299	0.176	0.308
	[0.093]	[0.301]	[0.196]	[0.177]	[0.116]	[0.128]	[0.152]	[0.115]	[0.083]	[0.132]
transitive recipr. triplets	0.033	-0.2	-0.217	0.212	0.228	0.046	-0.038	-0.082	-0.085	-0.255
	[0.131]	[0.367]	[0.287]	[0.246]	[0.191]	[0.196]	[0.261]	[0.18]	[0.14]	[0.197]
indegree - popularity	0.02	-0.112	0.108	-0.09	0.058	-0.102	-0.116	-0.338	-0.06	-0.11
	[0.045]	[0.171]	[0.133]	[0.119]	[0.074]	[0.096]	[0.092]	[0.15]	[0.063]	[0.095]
outdegree - popularity	-0.198	-0.231	0.027	-0.123	-0.151	-0.091	-0.133	-0.283	-0.099	-0.133
	[0.066]	[0.233]	[0.102]	[0.16]	[0.053]	[0.056]	[0.096]	[0.083]	[0.036]	[0.052]
outdegree - activity	-0.04	-0.081	0.232	-0.086	0.094	0.023	-0.088	0.009	0.029	0.068
	[0.034]	[0.115]	[0.108]	[0.089]	[0.032]	[0.03]	[0.062]	[0.034]	[0.022]	[0.036]
sex alter	-0.035	0.139	-0.467	-0.579	0.356	0.232	-0.177	-1.068	-0.08	-0.365
	[0.184]	[0.483]	[0.532]	[0.368]	[0.23]	[0.289]	[0.376]	[0.341]	[0.22]	[0.305]
sex ego	-0.135	0.02	-0.188	-0.164	0.044	-0.092	0.171	0.024	0.478	-0.011
	[0.222]	[0.539]	[0.584]	[0.373]	[0.221]	[0.256]	[0.399]	[0.229]	[0.201]	[0.229]
same sex	0.492	0.736	1.474	-0.041	0.978	0.165	0.844	0.127	0.674	0.902
	[0.176]	[0.569]	[0.583]	[0.278]	[0.253]	[0.171]	[0.273]	[0.271]	[0.199]	[0.321]
grade alter	-0.147	0.219	0.302	0.222	-0.021	-0.077	-0.136	0.463	0.156	0.176
	[0.17]	[0.24]	[0.24]	[0.183]	[0.128]	[0.11]	[0.308]	[0.196]	[0.176]	[0.15]
grade ego	-0.36	-0.309	-0.502	-0.09	-0.074	-0.034	-0.315	-0.032	0.164	0.228
	[0.196]	[0.245]	[0.435]	[0.187]	[0.128]	[0.074]	[0.314]	[0.131]	[0.159]	[0.139]
grade similarity	0.338	0.814	0.181	-0.188	0.311	0.882	0.577	0.859	-0.578	-1.401
	[0.348]	[0.775]	[0.626]	[0.563]	[0.631]	[0.356]	[0.448]	[0.458]	[0.321]	[0.693]
PGG alter	0.002	1.325	0.747	-0.04	-0.112	-0.002	0.182	-0.279	-0.013	-0.047
	[0.113]	[0.69]	[0.413]	[0.182]	[0.136]	[0.118]	[0.182]	[0.157]	[0.146]	[0.143]
PGG ego	0.241	0.151	-0.237	-0.323	-0.089	0.023	0.039	0.033	0.005	-0.303
	[0.139]	[0.487]	[0.265]	[0.219]	[0.109]	[0.092]	[0.261]	[0.122]	[0.091]	[0.149]
PGG similarity	-0.32	-1.407	0.638	0.304	0.355	-0.694	1.288	-0.532	-0.076	-0.87
	[0.415]	[0.963]	[0.996]	[0.528]	[0.365]	[0.353]	[0.607]	[0.511]	[0.288]	[0.552]
popularity alter	0.079	0.262	0.062	0.224	0.239	0.096	0.417	0.385	0.188	0.299
	[0.063]	[0.225]	[0.156]	[0.126]	[0.091]	[0.072]	[0.167]	[0.139]	[0.072]	[0.112]
popularity ego	0.191	-0.099	0.269	-0.017	-0.02	-0.021	0.144	-0.045	0.093	0.015
	[0.081]	[0.201]	[0.211]	[0.11]	[0.068]	[0.047]	[0.137]	[0.06]	[0.053]	[0.086]
popularity ego x popularity alter	0.019	0.295	-0.153	0.062	0.034	0.036	-0.057	0.025	-0.026	0.06
	[0.014]	[0.226]	[0.072]	[0.039]	[0.029]	[0.017]	[0.051]	[0.022]	[0.02]	[0.037]
int. PGG ego x PGG similarity	-0.062	-0.345	-1.225	0.554	0.699	-0.324	-0.282	0.606	0.297	0.073
	[0.413]	[1.526]	[0.89]	[0.499]	[0.48]	[0.328]	[0.679]	[0.378]	[0.354]	[0.416]
Overall maximum convergence ratio:	0.204	0.156	0.130	0.142	0.223	0.167	0.173	0.128	0.196	0.184

	11	12	13	14	15	16	17	18	19	20
outdegree (density)	-3.126	-2.413	-1.955	-1.403	0.082	-0.885	1.161	-0.239	-1.726	-3.018
	[0.822]	[0.76]	[0.691]	[0.705]	[1.358]	[0.617]	[2.093]	[2.014]	[2.113]	[3.548]
reciprocity	0.736	1.518	1.214	1.298	0.789	0.54	1.351	1.999	1.603	2.018
	[0.63]	[0.677]	[0.547]	[0.443]	[0.528]	[0.445]	[0.662]	[1.629]	[1.048]	[1.363]
transitive triplets	0.095	0.1	0.365	0.281	0.21	0.083	0.408	1.282	0.246	-0.251
	[0.15]	[0.095]	[0.164]	[0.134]	[0.081]	[0.084]	[0.223]	[1.018]	[0.291]	[0.441]
transitive recipr. triplets	0.06	-0.088	-0.267	-0.287	-0.163	0.162	0.015	-0.952	-0.134	0.468
	[0.191]	[0.146]	[0.257]	[0.189]	[0.091]	[0.117]	[0.354]	[1.427]	[0.439]	[0.688]
indegree - popularity	-0.069	0.061	0.088	-0.099	-0.254	-0.021	-0.101	-0.026	-0.312	0.407
	[0.12]	[0.068]	[0.082]	[0.117]	[0.161]	[0.053]	[0.235]	[0.159]	[0.279]	[0.268]
outdegree - popularity	-0.039	-0.081	-0.109	-0.02	0.089	-0.127	0.136	0.15	-0.158	0
	[0.073]	[0.045]	[0.121]	[0.09]	[0.063]	[0.04]	[0.799]	[0.931]	[0.146]	[NA]
outdegree - activity	0.109	0.067	-0.005	0.001	0.011	0.003	-0.522	-0.672	0.191	-0.254
	[0.037]	[0.025]	[0.043]	[0.037]	[0.025]	[0.026]	[0.266]	[0.568]	[0.119]	[0.391]
sex alter	0.535	0.162	0.905	-0.012	-0.447	0.328	0.33	0.133	-0.156	0.559
	[0.358]	[0.231]	[0.352]	[0.234]	[0.32]	[0.22]	[0.525]	[0.657]	[0.935]	[0.886]
sex ego	-0.231	0.417	0.822	0.202	-0.369	0.024	-0.829	-0.39	-1.328	-2.595
	[0.253]	[0.292]	[0.427]	[0.237]	[0.17]	[0.214]	[0.658]	[0.748]	[1.244]	[2.021]
same sex	1.311	0.394	0.33	0.911	0.231	0.422	0.555	0.807	0.299	2.202
	[0.307]	[0.229]	[0.36]	[0.238]	[0.157]	[0.184]	[0.347]	[0.4]	[0.635]	[1.025]
grade alter	-0.141	-0.217	0.047	0.317	-0.088	0.154	0.351	0.024	0.274	0.372
	[0.182]	[0.197]	[0.123]	[0.191]	[0.115]	[0.12]	[0.206]	[0.372]	[0.449]	[0.383]
grade ego	-0.261	0.302	0.049	-0.042	-0.222	0.162	0.17	0.066	0.433	0.311
	[0.148]	[0.229]	[0.16]	[0.124]	[0.099]	[0.139]	[0.286]	[0.467]	[0.467]	[0.576]
grade similarity	1.255	-0.261	1.007	-0.095	0.382	-0.104	-0.318	1.035	-1.561	0.97
	[0.563]	[0.415]	[0.681]	[0.477]	[0.402]	[0.396]	[0.919]	[0.984]	[1.201]	[1.384]
PGG alter	0.087	0.072	0.232	-0.111	0.296	0.087	-0.016	-0.006	0.09	-0.702
	[0.141]	[0.136]	[0.193]	[0.135]	[0.213]	[0.1]	[0.248]	[0.37]	[0.278]	[0.553]
PGG ego	-0.079	0.176	-0.135	0.047	0.181	0.128	0.22	0.084	-0.737	0.888
	[0.076]	[0.153]	[0.162]	[0.093]	[0.076]	[0.077]	[0.286]	[0.194]	[0.393]	[0.869]
PGG similarity	0.454	-0.429	0.375	0.131	-0.093	-0.258	0.746	1.187	0.719	2.341
	[0.377]	[0.447]	[0.658]	[0.334]	[0.312]	[0.31]	[0.646]	[0.789]	[1.308]	[1.952]
popularity alter	0.45	0.195	-0.035	0.13	0.409	0.267	0.154	-0.009	0.409	0.238
	[0.171]	[0.083]	[0.1]	[0.1]	[0.225]	[0.099]	[0.218]	[0.203]	[0.226]	[0.32]
popularity ego	-0.016	0.096	-0.211	0.039	0.051	-0.086	-0.13	-0.284	-0.448	-0.166
	[0.098]	[0.087]	[0.127]	[0.072]	[0.063]	[0.064]	[0.181]	[0.268]	[0.236]	[0.353]
popularity ego x popularity alter	-0.019	0.022	0.09	0.024	0.057	0.027	0.084	0	0.062	0.09
	[0.028]	[0.021]	[0.047]	[0.024]	[0.025]	[0.033]	[0.054]	[0.078]	[0.054]	[0.089]
int. PGG ego x PGG similarity	-0.154	-0.284	-1.285	0.373	0.26	-0.165	0.649	-0.326	-0.41	0.864
	[0.369]	[0.383]	[0.658]	[0.351]	[0.363]	[0.293]	[0.65]	[0.745]	[1.349]	[1.51]
Overall maximum convergence ratio:	0.204	0.179	0.124	0.167	0.193	0.179	0.167	0.197	0.136	0.173

.

Table 8: Results of the meta-analysis for friendship network and DG, Model 1

	est	se	N	p	tau2	Q	Qp
outdegree (density)	-1.264	0.2926	20	0.000	0.773	32.013	0.031
reciprocity	1.081	0.1254	20	0.000	0.000	10.242	0.947
transitive triplets	0.176	0.0282	20	0.000	0.002	14.303	0.766
transitive recipr. triplets	-0.051	0.0459	20	0.271	0.075	17.035	0.587
indegree - popularity	-0.015	0.0212	20	0.489	0.000	20.871	0.344
outdegree - popularity	-0.110	0.0156	19	0.000	0.000	14.854	0.672
outdegree - activity	0.027	0.0126	20	0.034	0.034	36.108	0.010
sex alter	0.054	0.0949	20	0.571	0.249	30.492	0.046
sex ego	0.038	0.0719	20	0.596	0.142	22.934	0.240
same sex	0.554	0.0867	20	0.000	0.257	37.208	0.007
grade alter	0.058	0.0374	20	0.119	0.000	15.528	0.688
grade ego	-0.041	0.0398	20	0.304	0.074	22.730	0.249
grade similarity	0.232	0.1511	20	0.125	0.412	32.290	0.029
DG alter	-0.003	0.0190	20	0.885	0.000	18.290	0.503
DG ego	-0.013	0.0193	20	0.504	0.034	20.590	0.360
DG similarity	-0.139	0.0852	20	0.102	0.000	12.999	0.839
popularity alter	0.195	0.0286	20	0.000	0.046	19.372	0.433
popularity ego	-0.004	0.0190	20	0.844	0.027	21.314	0.320
popularity ego x popularity alter	0.024	0.0068	20	0.000	0.010	24.887	0.164

Table 9: Goodness of fit statistics, Friendship & DG, Model 1

	Indegree distribution	Outdegree distribution	Geodesic Distance	Triad Census
1	0.832	0.643	0.954	0.986
2	0.866	0.464	0.440	0.861
3	0.836	0.642	0.213	0.575
4	0.754	0.352	0.896	0.994
5	0.494	0.074	0.349	0.227
6	0.719	0.776	0.392	0.205
7	0.051	0.563	0.630	0.648
8	0.404	0.649	0.984	0.958
9	0.842	0.951	0.579	0.944
10	0.824	0.716	0.521	0.977
11	0.524	0.003	0.347	0.836
12	0.192	0.461	0.713	0.783
13	0.229	0.272	0.168	0.767
14	0.887	0.762	0.936	0.866
15	0.942	0.962	0.950	0.870
16	0.851	0.522	0.357	0.258
17	0.592	0.754	0.996	0.996
18	0.996	0.580	0.890	0.955
19	0.989	0.658	0.451	0.741
20	0.791	0.745	0.947	0.742

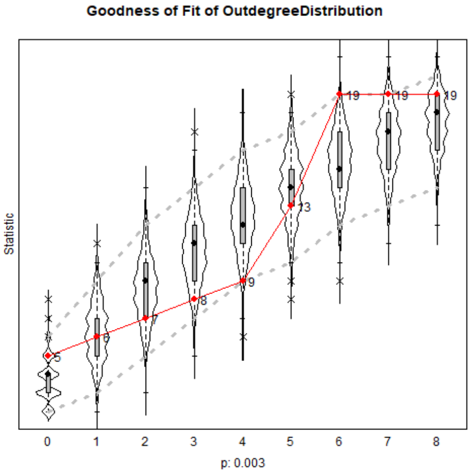

(a) Class 11

Figure 3: Classes with poor fit, Friendship & DG, Model 1

Table 10: Results of separate SAOMs for friendship network and DG, Model 1

	1	2	3	4	5	6	7	8	9	10
outdegree (density)	-0.893	-0.48	-5.373	0.92	-2.771	-0.214	0.3	2.42	-1.072	-1.789
	[0.573]	[1.849]	[2.063]	[1.359]	[0.78]	[0.922]	[1.097]	[1.388]	[0.633]	[0.96]
reciprocity	1.092	0.966	1.738	0.676	1.7	0.594	0.675	1.122	1.018	1.752
	[0.375]	[0.849]	[0.966]	[0.643]	[0.585]	[0.423]	[0.688]	[0.412]	[0.374]	[0.663]
transitive triplets	0.21	0.218	0.176	0.059	-0.016	0.109	0.256	0.313	0.188	0.269
	[0.088]	[0.265]	[0.221]	[0.167]	[0.105]	[0.123]	[0.145]	[0.119]	[0.081]	[0.132]
transitive recipr. triplets	0.051	-0.103	-0.261	0.168	0.232	0.029	0.005	-0.114	-0.072	-0.231
	[0.135]	[0.31]	[0.311]	[0.222]	[0.163]	[0.191]	[0.243]	[0.193]	[0.141]	[0.188]
indegree - popularity	0.012	0.112	0.125	-0.133	0.052	-0.108	-0.263	-0.443	-0.087	-0.091
	[0.043]	[0.148]	[0.132]	[0.134]	[0.082]	[0.121]	[0.207]	[0.178]	[0.07]	[0.096]
outdegree - popularity	-0.188	-0.161	0.03	-0.095	-0.144	-0.076	-0.044	-0.348	-0.107	-0.12
	[0.06]	[0.251]	[0.11]	[0.159]	[0.053]	[0.064]	[0.13]	[0.102]	[0.036]	[0.043]
outdegree - activity	-0.038	-0.073	0.249	-0.062	0.093	0.015	-0.084	0.006	0.024	0.07
	[0.033]	[0.124]	[0.141]	[0.077]	[0.029]	[0.031]	[0.057]	[0.034]	[0.023]	[0.032]
sex alter	-0.042	0.496	0.215	-0.559	0.461	0.251	-0.59	-1.274	-0.079	-0.295
	[0.177]	[0.417]	[0.537]	[0.381]	[0.228]	[0.307]	[0.598]	[0.417]	[0.217]	[0.233]
sex ego	-0.121	0.069	-0.419	-0.313	0.046	-0.082	0.13	0.068	0.514	0.135
	[0.214]	[0.458]	[0.638]	[0.369]	[0.234]	[0.199]	[0.355]	[0.231]	[0.212]	[0.183]
same sex	0.48	0.633	1.399	-0.069	0.954	0.107	0.83	0.1	0.638	0.924
	[0.17]	[0.495]	[0.608]	[0.275]	[0.236]	[0.159]	[0.271]	[0.271]	[0.199]	[0.282]
grade alter	-0.111	0	0.026	0.192	0.094	-0.071	0.011	0.464	0.188	0.164
	[0.178]	[0.185]	[0.204]	[0.164]	[0.139]	[0.106]	[0.362]	[0.214]	[0.17]	[0.147]
grade ego	-0.35	-0.35	-0.411	0.103	-0.199	-0.048	-0.372	-0.015	0.134	0.155
	[0.199]	[0.269]	[0.474]	[0.178]	[0.127]	[0.066]	[0.274]	[0.147]	[0.158]	[0.11]
grade similarity	0.37	0.586	0.117	-0.154	0.978	0.797	0.681	0.868	-0.659	-1.186
	[0.334]	[0.655]	[0.651]	[0.542]	[0.684]	[0.333]	[0.437]	[0.459]	[0.321]	[0.598]
DG alter	0.053	0.098	-0.064	0.165	-0.161	-0.046	0.431	-0.164	-0.077	-0.05
	[0.054]	[0.119]	[0.118]	[0.153]	[0.112]	[0.098]	[0.301]	[0.1]	[0.062]	[0.074]
DG ego	-0.036	0.25	0.116	-0.148	-0.004	0.068	-0.16	0.011	-0.096	-0.061
	[0.059]	[0.167]	[0.152]	[0.13]	[0.104]	[0.049]	[0.132]	[0.071]	[0.056]	[0.054]
DG similarity	-0.395	0.004	0.78	-0.502	-0.236	0.086	0.415	-0.317	-0.215	-0.691
	[0.291]	[0.547]	[0.696]	[0.544]	[0.499]	[0.23]	[0.542]	[0.363]	[0.316]	[0.409]
popularity alter	0.081	0.08	0.177	0.257	0.252	0.124	0.359	0.463	0.235	0.279
	[0.059]	[0.192]	[0.15]	[0.131]	[0.092]	[0.099]	[0.19]	[0.165]	[0.09]	[0.103]
popularity ego	0.108	-0.236	0.264	-0.042	0.023	-0.029	0.208	-0.048	0.115	0.012
	[0.069]	[0.217]	[0.243]	[0.098]	[0.066]	[0.052]	[0.161]	[0.059]	[0.055]	[0.069]
popularity ego x popularity alter	0.015	0.226	-0.16	0.057	0.035	0.034	-0.053	0.027	-0.025	0.055
	[0.013]	[0.171]	[0.084]	[0.038]	[0.034]	[0.017]	[0.06]	[0.022]	[0.021]	[0.032]
Overall maximum convergence ratio:	0.152	0.107	0.105	0.178	0.191	0.177	0.185	0.156	0.143	0.176

	11	12	13	14	15	16	17	18	19	20
outdegree (density)	-2.989	-2.845	-1.804	-1.264	-0.442	-1.03	0.729	-0.289	-1.388	-2.619
	[0.804]	[0.811]	[0.685]	[0.734]	[1.567]	[0.615]	[3.373]	[1.459]	[1.762]	[2.991]
reciprocity	0.766	1.932	1.146	1.305	0.963	0.495	1.501	2.08	1.619	1.742
	[0.718]	[0.708]	[0.574]	[0.412]	[0.564]	[0.406]	[0.844]	[1.252]	[1.026]	[1.283]
transitive triplets	0.082	0.139	0.345	0.281	0.216	0.07	0.408	1.335	0.355	-0.07
	[0.14]	[0.093]	[0.188]	[0.121]	[0.086]	[0.085]	[0.269]	[0.734]	[0.287]	[0.493]
transitive recipr. triplets	0.06	-0.168	-0.239	-0.292	-0.19	0.181	0.132	-0.983	-0.425	-0.063
	[0.184]	[0.135]	[0.273]	[0.179]	[0.096]	[0.123]	[0.384]	[0.964]	[0.512]	[0.54]
indegree - popularity	-0.066	0.077	0.068	-0.094	-0.239	-0.016	-0.433	-0.048	-0.301	0.353
	[0.126]	[0.066]	[0.091]	[0.104]	[0.291]	[0.054]	[0.832]	[0.143]	[0.274]	[0.248]
outdegree - popularity	-0.041	-0.085	-0.1	-0.029	0.131	-0.121	1.196	0.26	-0.112	0
	[0.085]	[0.042]	[0.12]	[0.091]	[0.16]	[0.039]	[2.314]	[0.525]	[0.133]	[NA]
outdegree - activity	0.106	0.081	-0.002	-0.006	0.008	0.011	-0.837	-0.725	0.13	-0.149
	[0.037]	[0.029]	[0.046]	[0.036]	[0.025]	[0.026]	[0.648]	[0.5]	[0.076]	[0.326]
sex alter	0.495	0.142	0.765	0.009	-0.304	0.295	1.321	0.083	0.198	0.115
	[0.332]	[0.225]	[0.342]	[0.235]	[0.388]	[0.203]	[2.217]	[0.55]	[0.88]	[0.768]
sex ego	-0.237	0.373	0.733	0.256	-0.366	0.088	-1.129	-0.575	-0.584	-1.413
	[0.245]	[0.267]	[0.374]	[0.245]	[0.191]	[0.202]	[1.395]	[1.003]	[0.684]	[1.116]
same sex	1.265	0.468	0.309	0.921	0.238	0.401	0.577	0.815	0.74	1.966
	[0.321]	[0.218]	[0.359]	[0.253]	[0.154]	[0.171]	[0.408]	[0.457]	[0.604]	[1.074]
grade alter	-0.148	-0.195	0.018	0.3	-0.056	0.16	0.607	0.064	0.192	0.158
	[0.184]	[0.2]	[0.127]	[0.169]	[0.11]	[0.115]	[0.849]	[0.324]	[0.363]	[0.297]
grade ego	-0.24	0.392	0.014	-0.035	-0.172	0.118	-0.109	-0.116	0.195	0.204
	[0.145]	[0.23]	[0.149]	[0.136]	[0.09]	[0.128]	[0.447]	[0.431]	[0.279]	[0.461]
grade similarity	1.197	-0.206	1.225	-0.091	0.116	-0.098	-0.2	0.928	-1.534	0.711
	[0.585]	[0.405]	[0.67]	[0.517]	[0.408]	[0.378]	[1.086]	[0.777]	[1.128]	[1.005]
DG alter	0.044	0.035	-0.136	-0.006	0.259	0.026	0.614	0.027	0.065	0.12
	[0.079]	[0.06]	[0.081]	[0.069]	[0.24]	[0.043]	[0.923]	[0.127]	[0.13]	[0.181]
DG ego	0.023	-0.118	-0.051	-0.016	-0.042	0.075	-0.54	-0.057	0.06	-0.029
	[0.061]	[0.073]	[0.088]	[0.067]	[0.065]	[0.043]	[0.398]	[0.156]	[0.112]	[0.23]
DG similarity	0.142	-0.07	0.196	-0.621	-0.268	0.001	0.44	-0.166	-0.826	-0.606
	[0.391]	[0.32]	[0.389]	[0.356]	[0.344]	[0.232]	[0.645]	[0.554]	[0.684]	[0.789]
popularity alter	0.454	0.203	-0.003	0.125	0.407	0.259	0.254	0.049	0.48	0.306
	[0.164]	[0.075]	[0.101]	[0.088]	[0.432]	[0.095]	[0.305]	[0.198]	[0.228]	[0.257]
popularity ego	-0.019	-0.026	-0.181	0.038	-0.001	-0.108	0.009	-0.166	-0.223	-0.155
	[0.094]	[0.052]	[0.127]	[0.061]	[0.054]	[0.066]	[0.246]	[0.243]	[0.128]	[0.264]
popularity ego x popularity alter	-0.022	0.024	0.073	0.031	0.06	0.027	0.059	0.021	0.062	0.12
	[0.028]	[0.018]	[0.043]	[0.025]	[0.024]	[0.033]	[0.06]	[0.069]	[0.044]	[0.077]
Overall maximum convergence ratio:	0.139	0.152	0.119	0.152	0.249	0.183	0.213	0.177	0.155	0.162

Table 11: Results of the meta-analysis for friendship network and DG, Model 2

	est	se	N	p	tau2	Q	Qp
outdegree (density)	-1.269	0.2814	20	0.000	0.708	31.198	0.038
reciprocity	1.077	0.1250	20	0.000	0.000	10.414	0.942
transitive triplets	0.180	0.0283	20	0.000	0.002	14.182	0.773
transitive recipr. triplets	-0.057	0.0456	20	0.214	0.069	16.799	0.603
indegree - popularity	-0.017	0.0210	20	0.410	0.000	21.775	0.296
outdegree - popularity	-0.106	0.0160	19	0.000	0.000	17.881	0.463
outdegree - activity	0.028	0.0121	20	0.022	0.031	32.566	0.027
sex alter	0.059	0.0899	20	0.512	0.217	28.531	0.074
sex ego	0.052	0.0733	20	0.479	0.142	21.730	0.298
same sex	0.557	0.0909	20	0.000	0.269	37.818	0.006
grade alter	0.065	0.0375	20	0.084	0.018	15.741	0.675
grade ego	-0.034	0.0378	20	0.367	0.055	22.626	0.254
grade similarity	0.207	0.1480	20	0.163	0.375	29.231	0.062
DG alter	0.034	0.0313	20	0.284	0.043	17.916	0.528
DG ego	-0.017	0.0195	20	0.373	0.029	18.172	0.511
DG similarity	-0.139	0.0914	20	0.127	0.059	18.549	0.486
popularity alter	0.200	0.0283	20	0.000	0.041	19.202	0.444
popularity ego	-0.003	0.0197	20	0.863	0.029	20.848	0.345
popularity ego x popularity alter	0.025	0.0071	20	0.001	0.012	25.850	0.134
int. DG ego x DG similarity	-0.129	0.0782	20	0.098	0.111	22.698	0.251

Table 12: Goodness of fit statistics, Friendship & DG, Model 2

	Indegree distribution	Outdegree distribution	Geodesic Distance	Triad Census
1	0.813	0.587	0.951	0.992
2	0.875	0.591	0.449	0.846
3	0.802	0.687	0.237	0.642
4	0.711	0.269	0.853	0.996
5	0.610	0.079	0.385	0.241
6	0.690	0.789	0.354	0.232
7	0.046	0.534	0.684	0.604
8	0.469	0.656	0.974	0.938
9	0.854	0.962	0.605	0.938
10	0.835	0.651	0.550	0.985
11	0.511	0.002	0.372	0.792
12	0.196	0.412	0.721	0.767
13	0.259	0.293	0.205	0.835
14	0.897	0.764	0.932	0.857
15	0.931	0.973	0.940	0.809
16	0.884	0.528	0.376	0.291
17	0.568	0.756	0.993	0.994
18	0.992	0.480	0.868	0.964
19	0.989	0.708	0.462	0.720
20	0.767	0.724	0.956	0.660

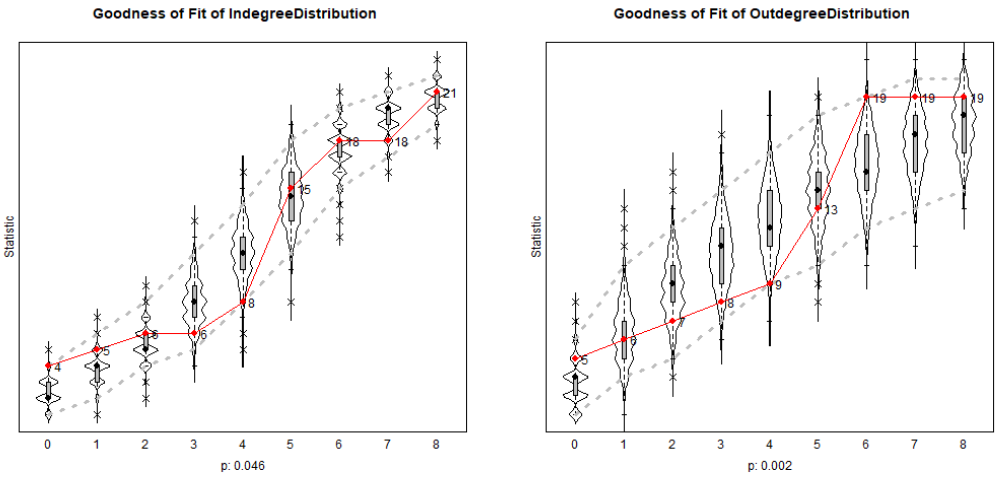

(a) Class 7

(b) Class 11

Figure 4: Classes with poor fit, Friendship & DG, Model 2

Table 13: Results of separate SAOMs for friendship network and DG, Model 2

	1	2	3	4	5	6	7	8	9	10
outdegree (density)	-0.852	-0.281	-5.371	0.936	-2.33	-0.22	0.091	2.486	-1.068	-1.675
	[0.586]	[2.033]	[1.949]	[1.359]	[0.781]	[0.833]	[1.091]	[1.482]	[0.686]	[0.864]
reciprocity	1.09	1.002	1.762	0.637	1.661	0.628	0.643	1.132	1.031	1.695
	[0.386]	[0.826]	[0.93]	[0.602]	[0.627]	[0.436]	[0.662]	[0.411]	[0.383]	[0.64]
transitive triplets	0.209	0.222	0.193	0.039	-0.001	0.125	0.238	0.322	0.185	0.277
	[0.095]	[0.241]	[0.213]	[0.164]	[0.119]	[0.123]	[0.161]	[0.125]	[0.087]	[0.119]
transitive recipr. triplets	0.06	-0.101	-0.283	0.176	0.253	0.015	0.029	-0.129	-0.069	-0.238
	[0.138]	[0.308]	[0.294]	[0.203]	[0.204]	[0.198]	[0.264]	[0.193]	[0.145]	[0.179]
indegree - popularity	0.01	0.109	0.122	-0.147	0.061	-0.114	-0.249	-0.448	-0.087	-0.095
	[0.044]	[0.139]	[0.146]	[0.141]	[0.082]	[0.105]	[0.192]	[0.187]	[0.069]	[0.088]
outdegree - popularity	-0.195	-0.188	0.03	-0.068	-0.16	-0.07	-0.037	-0.348	-0.107	-0.118
	[0.066]	[0.265]	[0.101]	[0.148]	[0.061]	[0.062]	[0.162]	[0.104]	[0.038]	[0.045]
outdegree - activity	-0.038	-0.078	0.249	-0.065	0.082	0.012	-0.078	0.003	0.024	0.064
	[0.034]	[0.122]	[0.127]	[0.082]	[0.032]	[0.031]	[0.067]	[0.036]	[0.024]	[0.03]
sex alter	-0.035	0.486	0.181	-0.564	0.399	0.279	-0.551	-1.274	-0.077	-0.281
	[0.174]	[0.425]	[0.47]	[0.388]	[0.211]	[0.33]	[0.633]	[0.458]	[0.216]	[0.245]
sex ego	-0.13	0.077	-0.444	-0.342	0.17	-0.103	0.12	0.026	0.516	0.147
	[0.21]	[0.464]	[0.595]	[0.347]	[0.242]	[0.223]	[0.348]	[0.245]	[0.212]	[0.195]
same sex	0.483	0.604	1.39	-0.077	0.951	0.091	0.854	0.115	0.622	0.952
	[0.177]	[0.515]	[0.615]	[0.267]	[0.274]	[0.161]	[0.27]	[0.278]	[0.201]	[0.311]
grade alter	-0.113	0.003	0.008	0.206	0.15	-0.075	0.042	0.464	0.194	0.151
	[0.189]	[0.183]	[0.217]	[0.171]	[0.133]	[0.1]	[0.408]	[0.218]	[0.175]	[0.145]
grade ego	-0.359	-0.343	-0.395	0.126	-0.282	-0.062	-0.338	-0.011	0.128	0.14
	[0.205]	[0.26]	[0.392]	[0.175]	[0.165]	[0.068]	[0.306]	[0.137]	[0.151]	[0.114]
grade similarity	0.373	0.591	0.278	-0.159	0.375	0.79	0.692	0.852	-0.655	-1.2
	[0.343]	[0.684]	[0.69]	[0.532]	[0.701]	[0.327]	[0.47]	[0.466]	[0.319]	[0.633]
DG alter	0.074	0.126	0.063	0.136	0.137	0.065	0.508	-0.088	-0.11	-0.153
	[0.083]	[0.187]	[0.162]	[0.165]	[0.188]	[0.117]	[0.317]	[0.128]	[0.081]	[0.094]
DG ego	-0.042	0.246	0.102	-0.137	-0.187	0.061	-0.189	-0.009	-0.083	-0.036
	[0.062]	[0.166]	[0.149]	[0.129]	[0.15]	[0.054]	[0.152]	[0.075]	[0.062]	[0.061]
DG similarity	-0.404	-0.002	0.695	-0.458	-1.062	0.249	0.392	-0.384	-0.182	-0.767
	[0.297]	[0.557]	[0.646]	[0.548]	[0.667]	[0.26]	[0.516]	[0.395]	[0.313]	[0.39]
popularity alter	0.086	0.083	0.195	0.266	0.247	0.131	0.357	0.469	0.237	0.291
	[0.064]	[0.19]	[0.158]	[0.131]	[0.076]	[0.097]	[0.179]	[0.17]	[0.09]	[0.102]
popularity ego	0.109	-0.232	0.249	-0.043	0.035	-0.031	0.209	-0.044	0.114	0.02
	[0.071]	[0.219]	[0.227]	[0.098]	[0.068]	[0.056]	[0.172]	[0.061]	[0.055]	[0.07]
popularity ego x popularity alter	0.015	0.227	-0.164	0.056	0.049	0.034	-0.057	0.029	-0.026	0.059
	[0.013]	[0.171]	[0.085]	[0.038]	[0.035]	[0.016]	[0.059]	[0.022]	[0.02]	[0.033]
int. DG ego x DG similarity	-0.077	-0.074	-0.489	0.187	-0.983	-0.372	-0.432	-0.265	0.128	0.418
	[0.242]	[0.428]	[0.514]	[0.392]	[0.529]	[0.231]	[0.439]	[0.284]	[0.221]	[0.303]
Overall maximum convergence ratio:	0.158	0.171	0.147	0.193	0.131	0.163	0.148	0.129	0.173	0.233

	11	12	13	14	15	16	17	18	19	20
outdegree (density)	-3.326	-2.808	-1.789	-1.294	-0.241	-1.096	1.062	-0.113	-1.353	-2.807
	[0.878]	[0.769]	[0.666]	[0.723]	[1.372]	[0.603]	[4.859]	[1.644]	[1.784]	[3.029]
reciprocity	0.774	1.943	1.172	1.302	0.943	0.534	1.481	2.233	1.684	1.824
	[0.61]	[0.623]	[0.603]	[0.449]	[0.479]	[0.398]	[1.084]	[1.287]	[0.998]	[1.381]
transitive triplets	0.07	0.132	0.363	0.277	0.22	0.065	0.4	1.379	0.384	-0.046
	[0.139]	[0.083]	[0.171]	[0.131]	[0.077]	[0.09]	[0.268]	[0.734]	[0.29]	[0.389]
transitive recipr. triplets	0.061	-0.153	-0.258	-0.27	-0.187	0.184	0.166	-1.016	-0.498	0.019
	[0.176]	[0.129]	[0.281]	[0.201]	[0.087]	[0.127]	[0.592]	[0.928]	[0.509]	[0.572]
indegree - popularity	-0.058	0.073	0.066	-0.089	-0.278	-0.015	-0.429	-0.05	-0.316	0.333
	[0.131]	[0.066]	[0.09]	[0.108]	[0.211]	[0.051]	[1.231]	[0.185]	[0.289]	[0.223]
outdegree - popularity	-0.036	-0.09	-0.101	-0.034	0.146	-0.122	1.178	0.261	-0.108	0
	[0.079]	[0.045]	[0.137]	[0.086]	[0.11]	[0.039]	[4.267]	[0.972]	[0.128]	[NA]
outdegree - activity	0.118	0.082	-0.004	-0.002	0.004	0.014	-0.869	-0.76	0.131	-0.186
	[0.038]	[0.028]	[0.042]	[0.036]	[0.026]	[0.026]	[1.179]	[0.617]	[0.077]	[0.299]
sex alter	0.541	0.152	0.747	0.01	-0.368	0.299	1.348	0.13	0.215	0.306
	[0.334]	[0.243]	[0.341]	[0.237]	[0.309]	[0.218]	[2.8]	[0.598]	[0.922]	[0.793]
sex ego	-0.142	0.356	0.726	0.247	-0.381	0.088	-1.142	-0.431	-0.622	-1.287
	[0.258]	[0.246]	[0.377]	[0.252]	[0.204]	[0.212]	[1.889]	[1.01]	[0.749]	[1.164]
same sex	1.29	0.45	0.293	0.899	0.238	0.413	0.565	0.88	0.775	2.032
	[0.342]	[0.226]	[0.381]	[0.252]	[0.15]	[0.179]	[0.766]	[0.465]	[0.593]	[0.821]
grade alter	-0.114	-0.203	0.022	0.289	-0.049	0.161	0.605	0.047	0.185	0.217
	[0.18]	[0.191]	[0.128]	[0.161]	[0.115]	[0.11]	[1.033]	[0.317]	[0.377]	[0.306]
grade ego	-0.201	0.393	0.022	-0.04	-0.153	0.12	-0.1	-0.178	0.233	0.42
	[0.149]	[0.218]	[0.157]	[0.131]	[0.096]	[0.126]	[0.379]	[0.447]	[0.298]	[0.47]
grade similarity	1.044	-0.195	1.217	-0.07	0.128	-0.076	-0.219	1.037	-1.637	0.954
	[0.613]	[0.407]	[0.701]	[0.528]	[0.409]	[0.384]	[1.18]	[0.876]	[1.145]	[1.212]
DG alter	0.218	0.064	-0.016	0.091	0.181	-0.001	0.695	0.378	0.14	-0.188
	[0.117]	[0.105]	[0.138]	[0.114]	[0.213]	[0.085]	[1.669]	[0.256]	[0.211]	[0.273]
DG ego	-0.019	-0.118	-0.069	-0.023	-0.012	0.076	-0.572	-0.102	0.061	0.085
	[0.067]	[0.065]	[0.088]	[0.067]	[0.07]	[0.046]	[1.014]	[0.157]	[0.112]	[0.263]
DG similarity	0.453	-0.044	0.2	-0.659	-0.026	0.003	0.3	-0.52	-0.915	-0.448
	[0.436]	[0.336]	[0.403]	[0.355]	[0.375]	[0.25]	[0.841]	[0.632]	[0.625]	[0.852]
popularity alter	0.453	0.208	-0.004	0.118	0.465	0.263	0.26	0.057	0.501	0.354
	[0.171]	[0.084]	[0.103]	[0.086]	[0.331]	[0.098]	[0.716]	[0.207]	[0.248]	[0.29]
popularity ego	-0.003	-0.03	-0.183	0.026	0	-0.115	0.008	-0.171	-0.233	-0.201
	[0.092]	[0.054]	[0.135]	[0.068]	[0.057]	[0.065]	[0.241]	[0.28]	[0.139]	[0.291]
popularity ego x popularity alter	-0.021	0.024	0.075	0.029	0.06	0.029	0.057	0.017	0.064	0.116
	[0.028]	[0.018]	[0.045]	[0.024]	[0.026]	[0.033]	[0.084]	[0.067]	[0.048]	[0.074]
int. DG ego x DG similarity	-0.614	-0.104	-0.415	-0.332	0.367	0.082	-0.385	-0.954	-0.213	1.354
	[0.307]	[0.28]	[0.412]	[0.311]	[0.292]	[0.225]	[0.439]	[0.562]	[0.484]	[0.888]
Overall maximum convergence ratio:	0.164	0.132	0.143	0.137	0.197	0.121	0.198	0.179	0.162	0.104

Table 14: Results of the meta-analysis for friendship network and TG, Model 1

	est	se	N	p	tau2	Q	Qp
outdegree (density)	-1.226	0.2878	20	0.000	0.759	33.638	0.020
reciprocity	1.097	0.1251	20	0.000	0.000	9.833	0.957
transitive triplets	0.175	0.0281	20	0.000	0.001	17.132	0.581
transitive recipr. triplets	-0.057	0.0473	20	0.233	0.080	16.691	0.611
indegree - popularity	-0.023	0.0211	20	0.272	0.013	24.255	0.187
outdegree - popularity	-0.101	0.0187	19	0.000	0.033	25.695	0.107
outdegree - activity	0.032	0.0112	20	0.005	0.026	37.437	0.007
sex alter	0.026	0.0915	20	0.773	0.227	32.125	0.030
sex ego	0.007	0.0668	20	0.920	0.106	20.148	0.386
same sex	0.559	0.0859	20	0.000	0.254	37.252	0.007
grade alter	0.040	0.0390	20	0.307	0.038	18.812	0.469
grade ego	-0.037	0.0402	20	0.357	0.082	25.539	0.144
grade similarity	0.199	0.1474	20	0.177	0.392	30.889	0.042
TG alter	-0.024	0.0242	20	0.311	0.063	34.548	0.016
TG ego	-0.030	0.0153	20	0.052	0.000	13.074	0.835
TG similarity	-0.081	0.1208	20	0.505	0.193	18.799	0.470
popularity alter	0.208	0.0264	20	0.000	0.021	20.013	0.394
popularity ego	-0.007	0.0178	20	0.688	0.000	16.220	0.643
popularity ego x popularity alter	0.023	0.0064	20	0.000	0.006	24.111	0.192

Table 15: Goodness of fit statistics, Friendship & TG, Model 1

	Indegree distribution	Outdegree distribution	Geodesic Distance	Triad Census
1	0.836	0.823	0.955	0.995
2	0.868	0.417	0.476	0.880
3	0.800	0.645	0.205	0.644
4	0.635	0.523	0.879	0.991
5	0.568	0.064	0.298	0.156
6	0.674	0.822	0.341	0.207
7	0.142	0.746	0.715	0.856
8	0.360	0.793	0.988	0.973
9	0.843	0.953	0.582	0.948
10	0.848	0.781	0.468	0.992
11	0.491	0.000	0.339	0.802
12	0.185	0.623	0.704	0.823
13	0.268	0.326	0.196	0.732
14	0.868	0.769	0.936	0.944
15	0.925	0.975	0.965	0.861
16	0.843	0.521	0.349	0.227
17	0.611	0.781	0.993	0.988
18	0.993	0.486	0.863	0.931
19	0.993	0.626	0.551	0.701
20	0.689	0.690	0.949	0.695

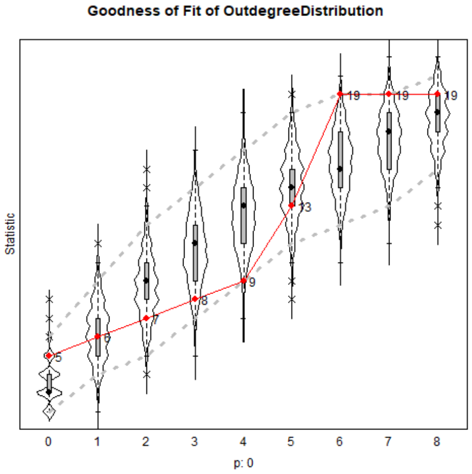

(a) Class 11

Figure 5: Classes with poor fit, Friendship & TG, Model 1

Table 16: Results of separate SAOMs for friendship network and TG, Model 1

	1	2	3	4	5	6	7	8	9	10
outdegree (density)	-0.837	-1.236	-4.849	1.059	-2.719	0.388	0.11	3.853	-1.144	-1.604
	[0.682]	[1.662]	[1.646]	[1.348]	[0.763]	[1.133]	[1.019]	[1.719]	[0.633]	[0.978]
reciprocity	1.133	0.982	1.589	0.541	1.646	0.579	0.768	1.166	1.051	1.69
	[0.385]	[0.81]	[0.783]	[0.558]	[0.576]	[0.552]	[0.845]	[0.444]	[0.384]	[0.573]
transitive triplets	0.225	0.221	0.122	0.045	-0.019	0.122	0.276	0.306	0.186	0.255
	[0.105]	[0.247]	[0.205]	[0.165]	[0.098]	[0.127]	[0.176]	[0.131]	[0.084]	[0.123]
transitive recipr. triplets	0.048	-0.107	-0.178	0.206	0.242	-0.002	-0.028	-0.089	-0.092	-0.197
	[0.146]	[0.304]	[0.29]	[0.211]	[0.185]	[0.237]	[0.31]	[0.21]	[0.155]	[0.177]
indegree - popularity	0.012	0.111	0.04	-0.121	0.061	-0.175	-0.238	-0.566	-0.088	-0.097
	[0.044]	[0.133]	[0.152]	[0.125]	[0.072]	[0.13]	[0.184]	[0.203]	[0.067]	[0.102]
outdegree - popularity	-0.214	-0.095	0.036	-0.19	-0.147	-0.12	-0.007	-0.469	-0.098	-0.129
	[0.076]	[0.252]	[0.101]	[0.134]	[0.061]	[0.063]	[0.202]	[0.132]	[0.036]	[0.043]
outdegree - activity	-0.045	-0.039	0.234	-0.031	0.09	0.023	-0.109	-0.012	0.027	0.065
	[0.046]	[0.1]	[0.102]	[0.073]	[0.029]	[0.028]	[0.087]	[0.04]	[0.022]	[0.03]
sex alter	-0.07	0.438	0.337	-0.675	0.317	-0.144	-0.702	-1.711	-0.03	-0.348
	[0.205]	[0.389]	[0.475]	[0.394]	[0.206]	[0.318]	[0.612]	[0.555]	[0.232]	[0.235]
sex ego	-0.204	0.104	-0.186	-0.255	-0.011	-0.062	0.114	-0.082	0.477	0.132
	[0.248]	[0.408]	[0.436]	[0.345]	[0.211]	[0.213]	[0.357]	[0.244]	[0.218]	[0.209]
same sex	0.515	0.624	1.417	-0.059	0.911	0.092	0.767	0.124	0.673	0.887
	[0.181]	[0.45]	[0.558]	[0.275]	[0.208]	[0.166]	[0.287]	[0.276]	[0.192]	[0.288]
grade alter	-0.213	-0.002	-0.118	0.179	-0.049	-0.118	0.597	0.464	0.227	0.137
	[0.18]	[0.181]	[0.21]	[0.179]	[0.109]	[0.109]	[0.736]	[0.245]	[0.184]	[0.14]
grade ego	-0.483	-0.29	-0.457	0.033	-0.18	-0.071	-0.64	-0.128	0.163	0.128
	[0.249]	[0.206]	[0.377]	[0.159]	[0.12]	[0.068]	[0.525]	[0.152]	[0.149]	[0.101]
grade similarity	0.385	0.661	0.319	-0.129	0.563	0.94	0.553	0.711	-0.624	-1.043
	[0.357]	[0.626]	[0.603]	[0.542]	[0.645]	[0.347]	[0.423]	[0.476]	[0.334]	[0.561]
TG alter	-0.083	0.037	-0.194	-0.067	-0.064	0.219	0.701	-0.395	-0.087	-0.044
	[0.067]	[0.144]	[0.113]	[0.068]	[0.044]	[0.106]	[0.527]	[0.159]	[0.069]	[0.09]
TG ego	-0.187	0.152	0.031	-0.013	-0.06	0.057	-0.326	-0.176	-0.012	-0.027
	[0.182]	[0.126]	[0.105]	[0.063]	[0.041]	[0.054]	[0.277]	[0.09]	[0.059]	[0.059]
TG similarity	-0.329	0.492	-0.093	-0.888	-0.63	0.325	0.807	-0.074	-0.432	0.162
	[0.699]	[0.707]	[0.883]	[0.595]	[0.365]	[0.312]	[0.604]	[0.559]	[0.362]	[0.387]
popularity alter	0.089	0.134	0.28	0.289	0.221	0.121	0.455	0.579	0.256	0.267
	[0.076]	[0.193]	[0.168]	[0.134]	[0.076]	[0.078]	[0.242]	[0.173]	[0.093]	[0.102]
popularity ego	0.114	-0.133	0.246	-0.056	0.005	0.002	0.203	-0.05	0.096	-0.009
	[0.108]	[0.185]	[0.191]	[0.096]	[0.068]	[0.045]	[0.213]	[0.066]	[0.056]	[0.068]
popularity ego x popularity alter	0.014	0.202	-0.16	0.054	0.034	0.036	-0.042	0.032	-0.022	0.053
	[0.013]	[0.161]	[0.074]	[0.037]	[0.03]	[0.017]	[0.06]	[0.023]	[0.021]	[0.033]
Overall maximum convergence ratio:	0.141	0.136	0.140	0.122	0.159	0.197	0.118	0.118	0.132	0.179

	11	12	13	14	15	16	17	18	19	20
outdegree (density)	-2.94	-2.577	-1.619	-1.298	-0.529	-1.03	0.047	-0.613	-1.105	-2.383
	[0.825]	[0.742]	[0.781]	[0.694]	[0.958]	[0.614]	[1.979]	[1.554]	[1.885]	[2.853]
reciprocity	0.682	1.709	1.182	1.263	0.998	0.535	1.538	2.236	1.649	1.722
	[0.603]	[0.687]	[0.582]	[0.43]	[0.472]	[0.389]	[0.815]	[1.165]	[0.997]	[1.272]
transitive triplets	0.075	0.12	0.366	0.272	0.22	0.081	0.451	1.439	0.365	-0.046
	[0.153]	[0.089]	[0.174]	[0.119]	[0.072]	[0.086]	[0.255]	[0.612]	[0.3]	[0.462]
transitive recipr. triplets	0.084	-0.124	-0.251	-0.267	-0.202	0.159	0.007	-1.049	-0.447	-0.007
	[0.2]	[0.142]	[0.27]	[0.182]	[0.08]	[0.117]	[0.373]	[0.905]	[0.504]	[0.524]
indegree - popularity	-0.073	0.065	0.071	-0.093	-0.2	-0.028	-0.317	-0.036	-0.331	0.298
	[0.129]	[0.065]	[0.077]	[0.1]	[0.129]	[0.054]	[0.387]	[0.159]	[0.302]	[0.256]
outdegree - popularity	-0.037	-0.089	-0.157	-0.038	0.09	-0.12	0.824	0.306	-0.122	0
	[0.076]	[0.041]	[0.126]	[0.086]	[0.064]	[0.04]	[1.005]	[0.474]	[0.126]	[NA]
outdegree - activity	0.105	0.077	-0.002	0.004	0.012	0.014	-0.618	-0.78	0.125	-0.157
	[0.036]	[0.027]	[0.045]	[0.036]	[0.023]	[0.026]	[0.317]	[0.37]	[0.076]	[0.291]
sex alter	0.586	0.12	0.867	-0.017	-0.297	0.301	0.533	0.127	0.203	0.133
	[0.363]	[0.231]	[0.375]	[0.233]	[0.289]	[0.212]	[0.706]	[0.655]	[0.874]	[0.722]
sex ego	-0.201	0.309	0.681	0.217	-0.304	0.046	-0.801	-0.786	-0.524	-1.268
	[0.244]	[0.237]	[0.402]	[0.238]	[0.168]	[0.2]	[0.733]	[0.964]	[0.719]	[1.077]
same sex	1.278	0.438	0.256	0.876	0.238	0.409	0.618	1.01	0.68	1.869
	[0.314]	[0.215]	[0.391]	[0.243]	[0.158]	[0.179]	[0.366]	[0.511]	[0.567]	[1.02]
grade alter	-0.136	-0.177	-0.016	0.361	0.033	0.182	0.266	0.052	0.149	0.195
	[0.181]	[0.192]	[0.129]	[0.183]	[0.117]	[0.121]	[0.305]	[0.346]	[0.374]	[0.302]
grade ego	-0.213	0.394	0.004	0.026	-0.168	0.141	0.085	-0.191	0.237	0.179
	[0.147]	[0.215]	[0.147]	[0.128]	[0.09]	[0.121]	[0.321]	[0.458]	[0.276]	[0.422]
grade similarity	1.078	-0.249	1.338	-0.293	0.021	-0.103	-0.172	1.186	-1.537	0.512
	[0.585]	[0.4]	[0.681]	[0.477]	[0.37]	[0.39]	[0.962]	[0.896]	[1.129]	[1.121]
TG alter	-0.013	0.061	-0.184	-0.086	0.059	0.083	-0.002	-0.184	0.073	0.2
	[0.059]	[0.048]	[0.089]	[0.074]	[0.062]	[0.048]	[0.17]	[0.168]	[0.127]	[0.192]
TG ego	-0.03	-0.005	-0.094	-0.082	-0.047	-0.002	-0.081	-0.176	-0.001	-0.083
	[0.056]	[0.05]	[0.085]	[0.072]	[0.044]	[0.05]	[0.188]	[0.168]	[0.091]	[0.19]
TG similarity	-0.302	-0.353	0.279	0.129	-0.968	0.364	0.141	-2.505	-0.682	-0.588
	[0.653]	[0.385]	[0.6]	[0.378]	[0.601]	[0.268]	[0.879]	[1.834]	[0.917]	[0.919]
popularity alter	0.456	0.21	0.027	0.126	0.276	0.288	0.336	0.044	0.484	0.374
	[0.166]	[0.082]	[0.113]	[0.082]	[0.179]	[0.099]	[0.348]	[0.188]	[0.232]	[0.239]
popularity ego	-0.032	-0.012	-0.159	0.011	0.015	-0.101	-0.094	-0.206	-0.233	-0.162
	[0.098]	[0.056]	[0.138]	[0.062]	[0.054]	[0.064]	[0.21]	[0.274]	[0.133]	[0.261]
popularity ego x popularity alter	-0.021	0.025	0.077	0.028	0.059	0.027	0.044	0.012	0.06	0.118
	[0.027]	[0.018]	[0.052]	[0.023]	[0.025]	[0.032]	[0.052]	[0.086]	[0.048]	[0.076]
Overall maximum convergence ratio:	0.162	0.193	0.191	0.172	0.196	0.185	0.137	0.141	0.158	0.097

Table 17: Results of the meta-analysis for friendship network and TG, Model 2

	est	se	N	p	tau2	Q	Qp
outdegree (density)	-1.227	0.309	20.000	0.000	0.843	33.660	0.020
reciprocity	1.080	0.128	20.000	0.000	0.000	9.396	0.966
transitive triplets	0.175	0.028	20.000	0.000	0.001	15.099	0.716
transitive recipr. triplets	-0.050	0.048	20.000	0.291	0.084	17.168	0.579
indegree - popularity	-0.026	0.022	20.000	0.247	0.018	24.336	0.184
outdegree - popularity	-0.101	0.017	19.000	0.000	0.020	23.785	0.162
outdegree - activity	0.032	0.011	20.000	0.004	0.026	32.112	0.030
sex alter	0.025	0.089	20.000	0.781	0.214	31.744	0.033
sex ego	0.011	0.068	20.000	0.873	0.098	20.187	0.383
same sex	0.563	0.085	20.000	0.000	0.245	35.240	0.013
grade alter	0.046	0.039	20.000	0.238	0.030	19.216	0.443
grade ego	-0.032	0.036	20.000	0.382	0.052	21.320	0.319
grade similarity	0.210	0.155	20.000	0.177	0.421	32.345	0.029
TG alter	0.012	0.024	20.000	0.606	0.001	20.605	0.359
TG ego	-0.041	0.017	20.000	0.015	0.000	14.739	0.739
TG similarity	0.009	0.112	20.000	0.933	0.000	15.070	0.718
popularity alter	0.206	0.026	20.000	0.000	0.000	19.179	0.445
popularity ego	-0.009	0.017	20.000	0.616	0.000	15.354	0.700
popularity ego x popularity alter	0.024	0.007	20.000	0.000	0.010	24.138	0.191
int. TG ego x TG similarity	-0.129	0.067	20.000	0.054	0.000	12.355	0.870

Table 18: Goodness of fit statistics, Friendship & TG, Model 2

	Indegree distribution	Outdegree distribution	Geodesic Distance	Triad Census
1	0.857	0.775	0.956	0.998
2	0.863	0.391	0.426	0.864
3	0.755	0.654	0.211	0.545
4	0.715	0.541	0.879	0.994
5	0.531	0.068	0.311	0.207
6	0.739	0.798	0.344	0.224
7	0.191	0.714	0.745	0.838
8	0.454	0.796	0.989	0.976
9	0.822	0.923	0.589	0.940
10	0.858	0.719	0.570	0.997
11	0.458	0.002	0.376	0.675
12	0.286	0.584	0.706	0.788
13	0.225	0.225	0.168	0.746
14	0.900	0.800	0.964	0.854
15	0.936	0.969	0.993	0.872
16	0.840	0.468	0.353	0.250
17	0.633	0.785	0.988	0.994
18	0.989	0.586	0.897	0.948
19	0.983	0.561	0.559	0.646
20	0.703	0.756	0.953	0.635

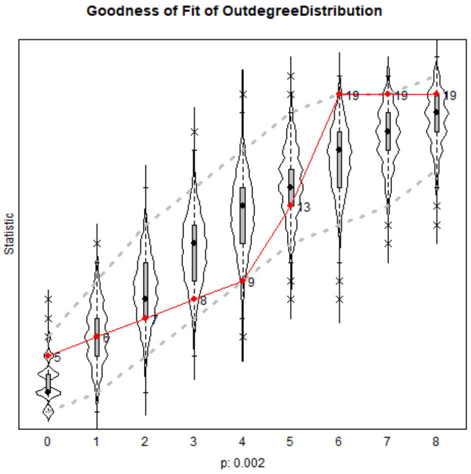

(a) Class 11

Figure 6: Classes with poor fit, Friendship & TG, Model 2

Table 19: Results of separate SAOMs for friendship network and TG, Model 2

	1	2	3	4	5	6	7	8	9	10
outdegree (density)	-0.702	-1.177	-4.713	1.078	-2.84	0.254	0.101	3.845	-1.09	-1.561
	[0.915]	[1.702]	[1.759]	[1.257]	[0.786]	[1.202]	[0.977]	[1.668]	[0.63]	[0.972]
reciprocity	1.131	0.951	1.654	0.535	1.639	0.604	0.751	1.155	1.06	1.678
	[0.413]	[0.807]	[0.946]	[0.578]	[0.587]	[0.463]	[0.655]	[0.437]	[0.395]	[0.65]
transitive triplets	0.232	0.225	0.135	0.046	-0.023	0.119	0.277	0.31	0.19	0.271
	[0.122]	[0.264]	[0.226]	[0.168]	[0.107]	[0.13]	[0.161]	[0.132]	[0.077]	[0.125]
transitive recipr. triplets	0.052	-0.094	-0.198	0.202	0.231	-0.005	-0.029	-0.092	-0.095	-0.225
	[0.152]	[0.309]	[0.301]	[0.209]	[0.177]	[0.196]	[0.242]	[0.204]	[0.139]	[0.194]
indegree - popularity	0.007	0.104	0.043	-0.121	0.067	-0.163	-0.232	-0.566	-0.094	-0.107
	[0.049]	[0.125]	[0.152]	[0.127]	[0.079]	[0.131]	[0.167]	[0.203]	[0.067]	[0.099]
outdegree - popularity	-0.225	-0.088	0.019	-0.188	-0.142	-0.117	-0.014	-0.463	-0.099	-0.122
	[0.089]	[0.242]	[0.108]	[0.129]	[0.056]	[0.067]	[0.16]	[0.127]	[0.037]	[0.037]
outdegree - activity	-0.045	-0.045	0.22	-0.033	0.089	0.026	-0.108	-0.013	0.026	0.062
	[0.063]	[0.107]	[0.115]	[0.07]	[0.028]	[0.032]	[0.073]	[0.038]	[0.021]	[0.029]
sex alter	-0.067	0.443	0.324	-0.675	0.296	-0.125	-0.722	-1.742	-0.032	-0.348
	[0.205]	[0.414]	[0.464]	[0.405]	[0.195]	[0.319]	[0.578]	[0.583]	[0.233]	[0.235]
sex ego	-0.233	0.102	-0.177	-0.299	-0.047	-0.052	0.143	-0.091	0.484	0.14
	[0.305]	[0.399]	[0.447]	[0.375]	[0.219]	[0.21]	[0.346]	[0.257]	[0.227]	[0.203]
same sex	0.52	0.592	1.398	-0.058	0.922	0.094	0.757	0.108	0.673	0.883
	[0.177]	[0.464]	[0.594]	[0.281]	[0.229]	[0.18]	[0.274]	[0.284]	[0.194]	[0.28]
grade alter	-0.223	-0.002	-0.147	0.175	-0.049	-0.116	0.574	0.458	0.224	0.15
	[0.184]	[0.178]	[0.219]	[0.181]	[0.115]	[0.116]	[0.592]	[0.235]	[0.18]	[0.142]
grade ego	-0.458	-0.291	-0.449	0.036	-0.166	-0.072	-0.641	-0.132	0.157	0.122
	[0.344]	[0.208]	[0.477]	[0.161]	[0.117]	[0.067]	[0.459]	[0.149]	[0.157]	[0.103]
grade similarity	0.391	0.669	0.41	-0.097	0.562	0.954	0.577	0.736	-0.624	-1.143
	[0.36]	[0.646]	[0.689]	[0.538]	[0.646]	[0.347]	[0.457]	[0.475]	[0.324]	[0.617]
TG alter	-0.16	0.006	-0.063	-0.058	0.022	0.201	0.617	-0.413	-0.098	0.003
	[0.273]	[0.162]	[0.21]	[0.08]	[0.062]	[0.136]	[0.429]	[0.183]	[0.085]	[0.111]
TG ego	-0.164	0.164	0.001	-0.023	-0.087	0.062	-0.269	-0.168	-0.008	-0.046
	[0.242]	[0.138]	[0.103]	[0.076]	[0.046]	[0.059]	[0.304]	[0.096]	[0.062]	[0.069]
TG similarity	-0.691	0.448	0.089	-0.846	-0.231	0.31	0.911	-0.095	-0.441	0.239
	[1.113]	[0.693]	[0.897]	[0.611]	[0.404]	[0.327]	[0.631]	[0.565]	[0.371]	[0.408]
popularity alter	0.107	0.132	0.272	0.288	0.225	0.12	0.447	0.574	0.263	0.27
	[0.082]	[0.196]	[0.163]	[0.135]	[0.076]	[0.071]	[0.226]	[0.182]	[0.095]	[0.104]
popularity ego	0.108	-0.141	0.232	-0.056	0.014	0.003	0.203	-0.052	0.096	0.006
	[0.129]	[0.201]	[0.208]	[0.098]	[0.063]	[0.042]	[0.202]	[0.067]	[0.056]	[0.067]
popularity ego x popularity alter	0.012	0.202	-0.151	0.053	0.03	0.036	-0.043	0.034	-0.021	0.057
	[0.014]	[0.168]	[0.077]	[0.038]	[0.03]	[0.017]	[0.06]	[0.023]	[0.021]	[0.031]
int. TG ego x TG similarity	0.428	0.145	-0.703	-0.064	-0.285	0.059	0.452	0.137	0.048	-0.217
	[1.28]	[0.432]	[1.194]	[0.257]	[0.178]	[0.254]	[0.602]	[0.36]	[0.243]	[0.33]
Overall maximum convergence ratio:	0.224	0.114	0.124	0.127	0.153	0.205	0.166	0.175	0.112	0.221

	11	12	13	14	15	16	17	18	19	20
outdegree (density)	-3.091	-2.604	-1.745	-1.274	-0.531	-1.046	0.094	-0.832	-1.163	-2.376
	[0.892]	[0.753]	[0.721]	[0.702]	[1.062]	[0.62]	[2.41]	[2.363]	[1.795]	[2.749]
reciprocity	0.704	1.629	1.179	1.291	1.072	0.523	1.583	2.288	1.693	1.929
	[0.712]	[0.627]	[0.609]	[0.415]	[0.586]	[0.388]	[0.904]	[1.631]	[1.012]	[1.358]
transitive triplets	0.086	0.112	0.362	0.282	0.234	0.077	0.458	1.454	0.357	-0.015
	[0.156]	[0.081]	[0.177]	[0.126]	[0.079]	[0.084]	[0.278]	[0.856]	[0.295]	[0.432]
transitive recipr. triplets	0.096	-0.107	-0.256	-0.287	-0.217	0.169	0.006	-1.103	-0.447	-0.031
	[0.214]	[0.135]	[0.284]	[0.18]	[0.096]	[0.11]	[0.375]	[1.098]	[0.501]	[0.572]
indegree - popularity	-0.086	0.066	0.076	-0.105	-0.195	-0.027	-0.308	-0.019	-0.341	0.298
	[0.151]	[0.064]	[0.08]	[0.11]	[0.137]	[0.056]	[0.498]	[0.159]	[0.291]	[0.232]
outdegree - popularity	-0.035	-0.087	-0.138	-0.031	0.083	-0.121	0.847	0.297	-0.119	0
	[0.088]	[0.043]	[0.117]	[0.084]	[0.063]	[0.042]	[1.639]	[0.774]	[0.122]	[NA]
outdegree - activity	0.105	0.08	0	0.003	0.008	0.014	-0.643	-0.817	0.131	-0.177
	[0.039]	[0.027]	[0.044]	[0.037]	[0.023]	[0.027]	[0.478]	[0.56]	[0.076]	[0.29]
sex alter	0.629	0.154	0.862	-0.025	-0.297	0.3	0.435	0.169	0.21	0.152
	[0.396]	[0.231]	[0.372]	[0.228]	[0.25]	[0.209]	[0.751]	[0.645]	[0.877]	[0.686]
sex ego	-0.19	0.341	0.703	0.218	-0.294	0.042	-0.768	-0.971	-0.557	-1.324
	[0.302]	[0.249]	[0.428]	[0.24]	[0.168]	[0.193]	[0.739]	[0.857]	[0.683]	[1.12]
same sex	1.274	0.438	0.279	0.884	0.256	0.409	0.691	1.04	0.74	1.842
	[0.325]	[0.231]	[0.388]	[0.239]	[0.155]	[0.173]	[0.421]	[0.54]	[0.584]	[0.879]
grade alter	-0.139	-0.167	-0.011	0.381	0.037	0.182	0.29	0.059	0.175	0.199
	[0.187]	[0.192]	[0.13]	[0.193]	[0.117]	[0.12]	[0.318]	[0.33]	[0.369]	[0.291]
grade ego	-0.167	0.384	0.022	0.027	-0.148	0.142	0.125	-0.378	0.13	0.154
	[0.151]	[0.217]	[0.148]	[0.128]	[0.093]	[0.121]	[0.314]	[0.488]	[0.304]	[0.414]
grade similarity	1.156	-0.267	1.497	-0.29	-0.012	-0.106	-0.114	1.406	-1.673	0.583
	[0.617]	[0.415]	[0.687]	[0.497]	[0.394]	[0.387]	[1.032]	[1.177]	[1.163]	[1.165]
TG alter	0.076	0.113	-0.069	-0.083	0.09	0.095	-0.097	-0.079	-0.065	0.355
	[0.085]	[0.085]	[0.11]	[0.108]	[0.069]	[0.082]	[0.146]	[0.141]	[0.154]	[0.28]
TG ego	-0.078	-0.016	-0.154	-0.082	-0.075	-0.004	-0.026	-0.368	0.073	-0.137
	[0.068]	[0.052]	[0.096]	[0.073]	[0.05]	[0.052]	[0.165]	[0.316]	[0.117]	[0.218]
TG similarity	0.409	-0.283	0.241	0.129	-0.688	0.379	-0.082	-2.307	-0.712	-0.69
	[0.776]	[0.378]	[0.6]	[0.385]	[0.589]	[0.271]	[0.966]	[1.851]	[0.899]	[0.862]
popularity alter	0.475	0.21	0.022	0.132	0.261	0.291	0.322	0.042	0.508	0.4
	[0.199]	[0.075]	[0.107]	[0.085]	[0.178]	[0.106]	[0.405]	[0.189]	[0.227]	[0.239]
popularity ego	-0.054	-0.018	-0.182	0.015	0.002	-0.106	-0.113	-0.201	-0.185	-0.159
	[0.106]	[0.051]	[0.134]	[0.061]	[0.055]	[0.061]	[0.226]	[0.261]	[0.136]	[0.3]
popularity ego x popularity alter	-0.026	0.024	0.069	0.028	0.058	0.027	0.038	0.01	0.065	0.118
	[0.029]	[0.019]	[0.048]	[0.023]	[0.023]	[0.032]	[0.067]	[0.081]	[0.047]	[0.068]
int. TG ego x TG similarity	-0.323	-0.205	-0.627	-0.021	-0.218	-0.043	0.594	-0.621	0.674	-0.638
	[0.18]	[0.279]	[0.441]	[0.336]	[0.201]	[0.218]	[0.586]	[0.592]	[0.519]	[0.721]
Overall maximum convergence ratio:	0.215	0.190	0.132	0.152	0.204	0.109	0.192	0.164	0.160	0.148

.

Table 20: Results of the meta-analysis for friendship network and TGB, Model 1

	est	se	N	p	tau2	Q	Qp
outdegree (density)	-1.190	0.2708	20	0.000	0.636	27.515	0.093
reciprocity	1.059	0.1260	20	0.000	0.000	10.405	0.942
transitive triplets	0.170	0.0290	20	0.000	0.001	15.244	0.707
transitive recipr. triplets	-0.049	0.0455	20	0.282	0.065	16.058	0.653
indegree - popularity	-0.030	0.0244	20	0.221	0.042	23.739	0.206
outdegree - popularity	-0.101	0.0216	19	0.000	0.053	26.077	0.098
outdegree - activity	0.029	0.0117	20	0.014	0.028	32.412	0.028
sex alter	0.038	0.0911	20	0.677	0.230	29.591	0.057
sex ego	0.014	0.0676	20	0.839	0.092	18.618	0.482
same sex	0.537	0.0838	20	0.000	0.233	33.674	0.020
grade alter	0.040	0.0384	20	0.298	0.000	17.345	0.566
grade ego	-0.022	0.0426	20	0.600	0.089	24.403	0.181
grade similarity	0.227	0.1504	20	0.131	0.400	30.754	0.043
TGB alter	0.003	0.0192	20	0.865	0.000	13.028	0.837
TGB ego	-0.036	0.0187	20	0.056	0.002	15.423	0.695
TGB similarity	-0.009	0.1895	20	0.961	0.369	23.582	0.213
popularity alter	0.194	0.0298	20	0.000	0.055	23.502	0.216
popularity ego	-0.019	0.0183	20	0.295	0.000	17.635	0.547
popularity ego x popularity alter	0.026	0.0072	20	0.000	0.012	25.011	0.160

Table 21: Goodness of fit statistics, Friendship & TGB, Model 1

	Indegree distribution	Outdegree distribution	Geodesic Distance	Triad Census
1	0.828	0.580	0.953	0.996
2	0.863	0.385	0.416	0.904
3	0.857	0.448	0.171	0.470
4	0.480	0.596	0.838	0.981
5	0.453	0.074	0.324	0.147
6	0.761	0.744	0.370	0.241
7	0.160	0.657	0.799	0.885
8	0.496	0.730	0.986	0.969
9	0.903	0.912	0.583	0.869
10	0.835	0.690	0.471	0.985
11	0.398	0.002	0.367	0.723
12	0.147	0.607	0.731	0.883
13	0.226	0.269	0.173	0.723
14	0.872	0.827	0.887	0.891
15	0.933	0.973	0.980	0.849
16	0.855	0.380	0.296	0.311
17	0.646	0.781	0.993	0.989
18	0.992	0.547	0.892	0.939
19	0.986	0.611	0.594	0.830
20	0.934	0.763	0.970	0.804

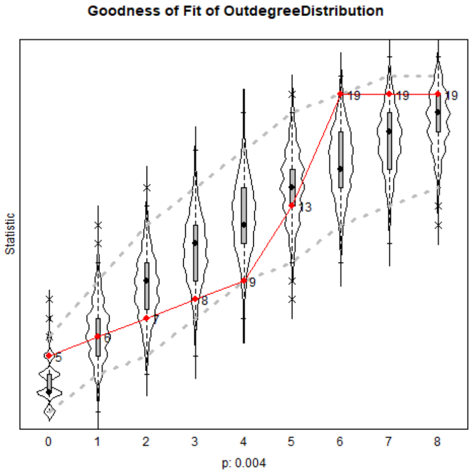

(a) Class 11

Figure 7: Classes with poor fit, Friendship & TGB, Model 1

Table 22: Results of separate SAOMs for friendship network and TGB, Model 1

	1	2	3	4	5	6	7	8	9	10
outdegree (density)	-0.907	-1.354	-7.465	0.865	-2.565	0.048	0.362	1.758	-1.169	-1.597
	[0.711]	[1.716]	[3.83]	[1.232]	[0.811]	[0.939]	[1.148]	[1.272]	[0.696]	[1.074]
reciprocity	1.125	0.956	2.092	0.552	1.59	0.6	0.742	1.109	1.077	1.743
	[0.378]	[0.831]	[1.237]	[0.59]	[0.539]	[0.455]	[0.767]	[0.407]	[0.465]	[0.65]
transitive triplets	0.218	0.217	0.117	0.004	-0.024	0.13	0.317	0.297	0.209	0.257
	[0.098]	[0.252]	[0.273]	[0.17]	[0.118]	[0.129]	[0.171]	[0.128]	[0.106]	[0.116]
transitive recipr. triplets	0.06	-0.069	-0.329	0.214	0.261	-0.017	-0.082	-0.094	-0.106	-0.205
	[0.144]	[0.298]	[0.398]	[0.213]	[0.2]	[0.205]	[0.306]	[0.201]	[0.175]	[0.163]
indegree - popularity	0.014	0.106	0.273	-0.163	0.061	-0.109	-0.174	-0.365	-0.085	-0.099
	[0.046]	[0.135]	[0.243]	[0.149]	[0.079]	[0.101]	[0.108]	[0.158]	[0.07]	[0.108]
outdegree - popularity	-0.209	-0.108	0.062	-0.119	-0.157	-0.12	-0.149	-0.303	-0.104	-0.129
	[0.074]	[0.221]	[0.117]	[0.12]	[0.052]	[0.065]	[0.112]	[0.093]	[0.039]	[0.044]
outdegree - activity	-0.035	-0.023	0.361	-0.007	0.085	0.012	-0.099	0.002	0.013	0.065
	[0.039]	[0.104]	[0.226]	[0.065]	[0.029]	[0.033]	[0.066]	[0.035]	[0.029]	[0.032]
sex alter	0.038	0.536	0.571	-0.521	0.284	0.062	-0.418	-1.039	-0.081	-0.268
	[0.187]	[0.405]	[0.728]	[0.386]	[0.203]	[0.289]	[0.476]	[0.37]	[0.233]	[0.239]
sex ego	-0.086	0.18	-1.138	-0.233	0.044	-0.135	0.163	0.044	0.442	0.197
	[0.256]	[0.412]	[1.454]	[0.345]	[0.24]	[0.209]	[0.435]	[0.229]	[0.325]	[0.182]
same sex	0.479	0.531	2.102	-0.07	0.901	0.158	0.679	0.107	0.627	0.844
	[0.185]	[0.528]	[1.133]	[0.273]	[0.232]	[0.171]	[0.272]	[0.268]	[0.208]	[0.27]
grade alter	-0.152	-0.026	0.007	0.158	-0.018	0.033	-0.259	0.511	0.187	0.134
	[0.18]	[0.192]	[0.208]	[0.189]	[0.118]	[0.126]	[0.319]	[0.212]	[0.205]	[0.138]
grade ego	-0.437	-0.325	-0.238	0.056	-0.123	0.108	-0.399	-0.01	0.13	0.135
	[0.209]	[0.207]	[0.691]	[0.158]	[0.12]	[0.098]	[0.307]	[0.141]	[0.192]	[0.093]
grade similarity	0.386	0.593	0.445	-0.098	0.264	1.292	0.515	0.8	-0.625	-0.89
	[0.346]	[0.65]	[0.679]	[0.501]	[0.644]	[0.403]	[0.423]	[0.445]	[0.434]	[0.527]
TGB alter	-0.035	0.099	0.065	-0.142	0.013	0.013	-0.14	-0.021	0.075	-0.044
	[0.054]	[0.191]	[0.12]	[0.137]	[0.043]	[0.104]	[0.127]	[0.073]	[0.121]	[0.099]
TGB ego	-0.132	0.239	0.629	0.007	-0.065	0.007	0.003	-0.039	-0.272	-0.087
	[0.076]	[0.22]	[0.539]	[0.127]	[0.041]	[0.085]	[0.158]	[0.057]	[0.351]	[0.08]
TGB similarity	-0.754	0.293	2.621	-0.556	-0.91	-1.695	0.068	0.852	1.593	-0.233
	[0.569]	[0.646]	[2.232]	[1.714]	[0.47]	[0.916]	[0.559]	[0.755]	[1.503]	[0.646]
popularity alter	0.093	0.119	0.037	0.359	0.24	0.076	0.575	0.399	0.206	0.271
	[0.074]	[0.184]	[0.183]	[0.162]	[0.093]	[0.067]	[0.217]	[0.149]	[0.085]	[0.109]
popularity ego	0.112	-0.143	0.525	-0.079	0.003	-0.036	0.17	-0.04	0.176	-0.023
	[0.083]	[0.188]	[0.54]	[0.098]	[0.06]	[0.049]	[0.152]	[0.061]	[0.119]	[0.064]
popularity ego x popularity alter	0.014	0.211	-0.153	0.057	0.04	0.034	-0.056	0.027	-0.026	0.053
	[0.013]	[0.174]	[0.095]	[0.037]	[0.031]	[0.017]	[0.056]	[0.022]	[0.022]	[0.03]
Overall maximum convergence ratio:	0.135	0.166	0.220	0.149	0.204	0.188	0.212	0.209	0.157	0.227

	11	12	13	14	15	16	17	18	19	20
outdegree (density)	-2.94	-2.685	-1.765	-1.248	-0.581	-1.171	0.134	-0.57	-0.699	-1.12
	[0.86]	[0.726]	[0.737]	[0.72]	[0.8]	[0.637]	[2.807]	[1.635]	[1.89]	[2.987]
reciprocity	0.705	1.526	1.191	1.134	0.858	0.513	1.5	2.254	1.939	2.699
	[0.687]	[0.594]	[0.581]	[0.398]	[0.465]	[0.393]	[1.045]	[1.263]	[1.055]	[1.857]
transitive triplets	0.061	0.101	0.337	0.242	0.203	0.064	0.433	1.508	0.409	0.117
	[0.151]	[0.082]	[0.201]	[0.12]	[0.074]	[0.088]	[0.299]	[0.731]	[0.293]	[0.453]
transitive recipr. triplets	0.087	-0.076	-0.239	-0.234	-0.176	0.168	0.057	-1.105	-0.543	0.071
	[0.202]	[0.122]	[0.297]	[0.175]	[0.085]	[0.119]	[0.427]	[0.917]	[0.504]	[0.726]
indegree - popularity	-0.094	0.067	0.099	-0.084	-0.176	-0.018	-0.35	-0.042	-0.422	0.177
	[0.149]	[0.067]	[0.074]	[0.098]	[0.099]	[0.056]	[0.833]	[0.181]	[0.316]	[0.234]
outdegree - popularity	-0.031	-0.071	-0.143	-0.058	0.082	-0.117	1.053	0.364	-0.143	0
	[0.084]	[0.043]	[0.108]	[0.075]	[0.052]	[0.037]	[2.949]	[0.537]	[0.131]	[NA]
outdegree - activity	0.112	0.078	-0.003	0.001	0.01	0.02	-0.734	-0.819	0.117	-0.346
	[0.037]	[0.025]	[0.048]	[0.034]	[0.022]	[0.026]	[0.902]	[0.485]	[0.075]	[0.329]
sex alter	0.555	-0.019	0.975	0.03	-0.343	0.306	0.478	0.033	0.682	0.346
	[0.412]	[0.253]	[0.378]	[0.228]	[0.253]	[0.212]	[0.739]	[0.717]	[0.884]	[0.824]
sex ego	-0.152	0.227	0.905	0.158	-0.319	0.101	-0.441	-0.826	-0.563	-1.878
	[0.269]	[0.241]	[0.441]	[0.236]	[0.153]	[0.207]	[0.799]	[1.1]	[0.802]	[1.393]
same sex	1.306	0.445	0.295	0.941	0.23	0.419	0.6	1.094	0.656	1.538
	[0.332]	[0.22]	[0.377]	[0.245]	[0.154]	[0.188]	[0.418]	[0.632]	[0.545]	[0.736]
grade alter	-0.243	-0.071	-0.046	0.236	-0.069	0.157	0.017	-0.182	0.36	0.561
	[0.194]	[0.194]	[0.134]	[0.155]	[0.107]	[0.122]	[0.525]	[0.379]	[0.451]	[0.445]
grade ego	-0.206	0.395	-0.046	-0.052	-0.152	0.152	0.397	-0.284	0.149	-0.264
	[0.147]	[0.215]	[0.159]	[0.114]	[0.09]	[0.138]	[0.506]	[0.582]	[0.297]	[0.496]
grade similarity	1.088	-0.265	1.03	-0.072	0.04	-0.169	-0.218	1.273	-2.113	0.747
	[0.579]	[0.382]	[0.638]	[0.463]	[0.39]	[0.371]	[1.203]	[0.995]	[1.242]	[1.136]
TGB alter	-0.03	0.09	-0.099	0.009	0.086	-0.007	-0.247	-0.162	-0.149	0.501
	[0.135]	[0.052]	[0.096]	[0.073]	[0.082]	[0.074]	[0.56]	[0.218]	[0.132]	[0.382]
TGB ego	0.055	0.031	-0.121	-0.027	-0.059	-0.11	0.305	-0.106	0.16	-0.622
	[0.124]	[0.046]	[0.099]	[0.081]	[0.053]	[0.102]	[0.348]	[0.24]	[0.126]	[0.508]
TGB similarity	0.564	0.759	0.141	1.504	-0.407	0.039	0.232	-3.336	1.593	-0.238
	[1.625]	[0.524]	[0.746]	[0.912]	[0.321]	[0.75]	[0.919]	[2.791]	[1]	[1.096]
popularity alter	0.487	0.202	-0.005	0.141	0.35	0.264	0.426	0.058	0.544	0.366
	[0.186]	[0.078]	[0.099]	[0.082]	[0.174]	[0.093]	[0.815]	[0.215]	[0.256]	[0.322]
popularity ego	-0.027	-0.004	-0.185	0.037	-0.005	-0.112	-0.191	-0.241	-0.255	-0.16
	[0.092]	[0.054]	[0.146]	[0.06]	[0.063]	[0.065]	[0.277]	[0.304]	[0.13]	[0.388]
popularity ego x popularity alter	-0.021	0.026	0.085	0.033	0.069	0.025	0.049	0.031	0.077	0.119
	[0.028]	[0.018]	[0.049]	[0.024]	[0.025]	[0.032]	[0.066]	[0.08]	[0.048]	[0.086]
Overall maximum convergence ratio:	0.189	0.210	0.151	0.166	0.148	0.115	0.179	0.217	0.124	0.189

Table 23: Results of the meta-analysis for friendship network and TGB, Model 2

	est	se	N	p	tau2	Q	Qp
outdegree (density)	-1.281	0.2837	20	0.000	0.679	28.194	0.080
reciprocity	1.143	0.1347	20	0.000	0.000	8.325	0.983
transitive triplets	0.183	0.0304	20	0.000	0.002	11.371	0.911
transitive recipr. triplets	-0.056	0.0463	20	0.227	0.057	14.928	0.727
indegree - popularity	-0.021	0.0245	20	0.395	0.040	23.327	0.223
outdegree - popularity	-0.107	0.0170	19	0.000	0.011	20.612	0.299
outdegree - activity	0.027	0.0121	20	0.026	0.029	31.209	0.038
sex alter	0.028	0.1044	20	0.789	0.286	31.356	0.037
sex ego	0.028	0.0800	20	0.726	0.171	25.605	0.142
same sex	0.541	0.0895	20	0.000	0.255	35.496	0.012
grade alter	0.040	0.0402	20	0.315	0.000	17.556	0.552
grade ego	-0.029	0.0448	20	0.521	0.088	23.292	0.225
grade similarity	0.214	0.1665	20	0.198	0.476	35.668	0.012
TGB alter	-0.003	0.0243	18	0.911	0.000	9.682	0.916
TGB ego	-0.033	0.0236	18	0.162	0.009	18.980	0.330
TGB similarity	-0.130	0.2151	18	0.546	0.410	18.169	0.378
popularity alter	0.181	0.0279	20	0.000	0.036	21.921	0.288
popularity ego	-0.016	0.0212	20	0.445	0.032	20.422	0.370
popularity ego x popularity alter	0.024	0.0070	20	0.001	0.010	23.798	0.204
int. TGB ego x TGB similarity	-0.092	0.1046	18	0.379	0.258	25.546	0.083

Table 24: Goodness of fit statistics, Friendship & TGB, Model 2

	Indegree distribution	Outdegree distribution	Geodesic Distance	Triad Census
1	0.844	0.534	0.963	0.996
2	0.874	0.341	0.417	0.910
3	0.866	0.375	0.185	0.540
4	0.526	0.622	0.854	0.983
5	0.468	0.087	0.352	0.247
6	0.773	0.738	0.374	0.158
7	0.125	0.666	0.790	0.839
8	0.501	0.760	0.985	0.955
9	0.886	0.941	0.557	0.951
10	0.855	0.752	0.500	0.987
11	0.567	0.004	0.481	0.643
12	0.152	0.589	0.745	0.897
13	0.130	0.229	0.131	0.713
14	0.894	0.790	0.944	0.879
15	0.928	0.984	0.974	0.834
16	0.816	0.474	0.256	0.109
17	0.611	0.736	0.989	0.992
18	0.990	0.515	0.916	0.956
19	0.986	0.616	0.552	0.784
20	0.892	0.832	0.974	0.808

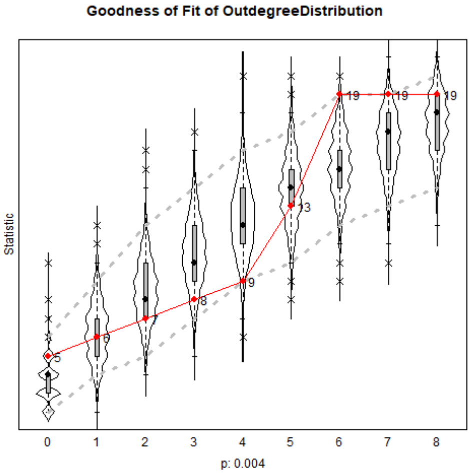

(a) Class 11

Figure 8: Classes with poor fit, Friendship & TGB, Model 2

Table 25: Results of separate SAOMs for friendship network and TGB, Model 2

	1	2	3	4	5	6	7	8	9	10
outdegree (density)	-0.913	-1.422	-7.683	0.937	-2.437	0.195	0.281	1.766	-1.323	-1.778
	[0.647]	[1.66]	[4.211]	[1.309]	[0.735]	[1.267]	[1.088]	[1.256]	[0.573]	[0.923]
reciprocity	1.13	0.918	2.117	0.566	1.56	0.586	0.755	1.1	1.058	1.79
	[0.414]	[0.829]	[1.483]	[0.565]	[0.571]	[0.536]	[0.724]	[0.426]	[0.425]	[0.551]
transitive triplets	0.219	0.199	0.11	0.015	-0.01	0.126	0.316	0.296	0.18	0.279
	[0.103]	[0.247]	[0.281]	[0.171]	[0.121]	[0.147]	[0.16]	[0.123]	[0.087]	[0.142]
transitive recipr. triplets	0.06	-0.055	-0.339	0.196	0.24	-0.006	-0.077	-0.086	-0.094	-0.247
	[0.147]	[0.295]	[0.444]	[0.203]	[0.19]	[0.243]	[0.301]	[0.193]	[0.154]	[0.197]
indegree - popularity	0.016	0.114	0.284	-0.161	0.063	-0.107	-0.171	-0.374	-0.062	-0.088
	[0.044]	[0.136]	[0.236]	[0.16]	[0.071]	[0.121]	[0.115]	[0.154]	[0.058]	[0.09]
outdegree - popularity	-0.213	-0.103	0.068	-0.114	-0.16	-0.123	-0.152	-0.307	-0.094	-0.125
	[0.073]	[0.237]	[0.124]	[0.135]	[0.059]	[0.069]	[0.111]	[0.096]	[0.034]	[0.04]
outdegree - activity	-0.041	-0.017	0.371	-0.008	0.08	-0.001	-0.096	0.004	0.027	0.065
	[0.042]	[0.102]	[0.261]	[0.069]	[0.032]	[0.037]	[0.056]	[0.034]	[0.022]	[0.033]
sex alter	0.022	0.528	0.561	-0.508	0.299	0.032	-0.425	-1.055	-0.118	-0.263
	[0.193]	[0.425]	[0.738]	[0.387]	[0.211]	[0.31]	[0.481]	[0.35]	[0.208]	[0.226]
sex ego	-0.003	0.178	-1.186	-0.341	-0.017	-0.217	0.125	0.037	0.48	0.188
	[0.267]	[0.394]	[1.516]	[0.373]	[0.215]	[0.232]	[0.424]	[0.226]	[0.203]	[0.186]
same sex	0.472	0.526	2.146	-0.083	0.9	0.132	0.719	0.106	0.668	0.859
	[0.181]	[0.501]	[1.141]	[0.276]	[0.228]	[0.184]	[0.277]	[0.273]	[0.199]	[0.27]
grade alter	-0.175	-0.021	0.002	0.156	0.01	0.006	-0.26	0.53	0.194	0.136
	[0.19]	[0.188]	[0.215]	[0.185]	[0.113]	[0.116]	[0.339]	[0.215]	[0.172]	[0.143]
grade ego	-0.482	-0.327	-0.241	0.045	-0.085	0.133	-0.399	-0.007	0.144	0.147
	[0.235]	[0.217]	[0.636]	[0.166]	[0.122]	[0.107]	[0.301]	[0.132]	[0.15]	[0.107]
grade similarity	0.426	0.585	0.45	-0.15	0.021	1.364	0.545	0.774	-0.585	-0.89
	[0.366]	[0.651]	[0.677]	[0.514]	[0.671]	[0.412]	[0.426]	[0.455]	[0.317]	[0.559]
TGB alter	0.002	0.124	0.077	-0.178	-0.049	0.067	-0.088	-0.002	0	0.003
	[0.062]	[0.249]	[0.15]	[0.164]	[0.048]	[0.098]	[0.149]	[0.075]	[NA]	[0.113]
TGB ego	-0.212	0.238	0.641	0.103	0.013	-0.117	-0.041	-0.057	0	-0.114
	[0.127]	[0.229]	[0.554]	[0.169]	[0.051]	[0.132]	[0.134]	[0.063]	[NA]	[0.087]
TGB similarity	-0.597	0.309	2.736	-0.615	-1.315	-1.186	-0.09	0.895	0	-0.128
	[0.596]	[0.634]	[2.437]	[1.885]	[0.489]	[1.02]	[0.574]	[0.772]	[NA]	[0.648]
popularity alter	0.094	0.117	0.035	0.361	0.235	0.074	0.586	0.408	0.194	0.256
	[0.069]	[0.188]	[0.179]	[0.18]	[0.08]	[0.07]	[0.244]	[0.145]	[0.073]	[0.1]
popularity ego	0.134	-0.144	0.55	-0.101	0.014	-0.094	0.165	-0.042	0.091	-0.025
	[0.104]	[0.191]	[0.547]	[0.107]	[0.062]	[0.066]	[0.149]	[0.061]	[0.048]	[0.063]
popularity ego x popularity alter	0.015	0.213	-0.154	0.059	0.035	0.031	-0.057	0.027	-0.026	0.052
	[0.014]	[0.172]	[0.103]	[0.038]	[0.031]	[0.016]	[0.06]	[0.022]	[0.02]	[0.031]
int. TGB ego x TGB similarity	-0.28	-0.114	-0.09	0.316	0.424	-0.357	-0.487	-0.153	0	-0.213
	[0.279]	[0.746]	[0.615]	[0.355]	[0.19]	[0.242]	[0.482]	[0.265]	[NA]	[0.244]
Overall maximum convergence ratio:	0.139	0.121	0.145	0.176	0.150	0.146	0.163	0.163	0.086	0.234

	11	12	13	14	15	16	17	18	19	20
outdegree (density)	-3.726	-2.633	-1.84	-1.291	-0.444	-1.518	0.093	-0.396	-0.639	-1.094
	[1.048]	[0.72]	[0.787]	[0.671]	[0.962]	[0.882]	[4.752]	[1.83]	[2.04]	[2.888]
reciprocity	0.578	1.52	1.235	1.293	0.896	1.102	1.519	2.52	1.922	2.845
	[0.768]	[0.621]	[0.63]	[0.442]	[0.507]	[0.519]	[1.753]	[2.01]	[1.027]	[1.644]
transitive triplets	0.043	0.102	0.333	0.279	0.219	0.131	0.432	1.695	0.393	0.112
	[0.199]	[0.08]	[0.177]	[0.13]	[0.076]	[0.134]	[0.522]	[1.196]	[0.287]	[0.374]
transitive recipr. triplets	0.16	-0.066	-0.216	-0.285	-0.187	0.193	0.061	-1.258	-0.476	0.059
	[0.273]	[0.121]	[0.277]	[0.189]	[0.086]	[0.155]	[0.465]	[1.455]	[0.482]	[0.627]
indegree - popularity	-0.126	0.065	0.103	-0.098	-0.196	-0.024	-0.348	-0.053	-0.418	0.167
	[0.163]	[0.066]	[0.069]	[0.108]	[0.144]	[0.083]	[1.168]	[0.174]	[0.346]	[0.224]
outdegree - popularity	-0.017	-0.075	-0.154	-0.022	0.091	-0.188	1.057	0.364	-0.155	0
	[0.101]	[0.044]	[0.114]	[0.097]	[0.076]	[0.095]	[3.817]	[0.552]	[0.129]	[NA]
outdegree - activity	0.13	0.077	-0.003	-0.004	0.008	0.022	-0.739	-1.009	0.119	-0.349
	[0.045]	[0.025]	[0.049]	[0.035]	[0.023]	[0.034]	[0.976]	[0.716]	[0.079]	[0.337]
sex alter	0.855	-0.019	0.994	-0.006	-0.393	0.5	0.5	0.121	0.668	0.31
	[0.445]	[0.245]	[0.389]	[0.225]	[0.324]	[0.428]	[1.497]	[0.667]	[0.893]	[0.783]
sex ego	-0.534	0.224	1.046	0.192	-0.341	0.146	-0.579	-1.177	-0.491	-2.126
	[0.362]	[0.244]	[0.473]	[0.236]	[0.167]	[0.303]	[1.39]	[1.069]	[0.829]	[1.572]
same sex	1.508	0.441	0.244	0.896	0.23	0.373	0.587	1.227	0.618	1.512
	[0.38]	[0.217]	[0.39]	[0.234]	[0.154]	[0.257]	[0.558]	[0.853]	[0.576]	[0.699]
grade alter	-0.311	-0.07	-0.046	0.305	-0.067	0.111	0.018	-0.154	0.398	0.592
	[0.24]	[0.205]	[0.149]	[0.173]	[0.109]	[0.199]	[0.538]	[0.458]	[0.464]	[0.463]
grade ego	-0.269	0.384	-0.087	-0.047	-0.152	0.225	0.372	-0.517	0.139	-0.229
	[0.181]	[0.216]	[0.17]	[0.12]	[0.086]	[0.519]	[0.53]	[0.666]	[0.311]	[0.511]
grade similarity	1.534	-0.267	1.164	-0.112	0.099	-0.544	-0.245	1.553	-2.239	0.708
	[0.717]	[0.391]	[0.661]	[0.455]	[0.394]	[0.588]	[1.07]	[1.254]	[1.311]	[1.18]
TGB alter	0.067	0.082	-0.031	0	-0.016	-0.622	-0.199	0.034	-0.213	0.671
	[0.195]	[0.063]	[0.127]	[NA]	[0.1]	[0.828]	[0.605]	[0.238]	[0.175]	[0.465]
TGB ego	-0.262	0.034	-0.242	0	-0.057	-0.444	0.229	-0.549	0.171	-0.727
	[0.167]	[0.054]	[0.131]	[NA]	[0.056]	[0.823]	[0.403]	[0.603]	[0.135]	[0.591]
TGB similarity	0.774	0.693	0.359	0	-0.529	0.894	0.346	-2.168	1.568	-0.322
	[2.094]	[0.582]	[0.821]	[NA]	[0.311]	[3.119]	[1.542]	[3.425]	[1.082]	[1.086]
popularity alter	0.55	0.199	-0.001	0.126	0.387	0.336	0.433	0.051	0.531	0.411
	[0.212]	[0.078]	[0.109]	[0.079]	[0.234]	[0.263]	[1.187]	[0.229]	[0.275]	[0.314]
popularity ego	-0.071	-0.007	-0.131	0.03	-0.035	-0.168	-0.188	-0.285	-0.272	-0.196
	[0.129]	[0.055]	[0.144]	[0.063]	[0.065]	[0.116]	[0.325]	[0.306]	[0.139]	[0.353]
popularity ego x popularity alter	-0.039	0.026	0.083	0.029	0.061	0.035	0.049	0.036	0.083	0.125
	[0.035]	[0.019]	[0.051]	[0.023]	[0.027]	[0.037]	[0.059]	[0.089]	[0.054]	[0.084]
int. TGB ego x TGB similarity	-0.752	0.032	-0.559	0	0.403	5.232	-0.256	-1.214	0.255	-0.808
	[0.301]	[0.162]	[0.433]	[NA]	[0.218]	[6.739]	[0.508]	[1.051]	[0.408]	[0.943]
Overall maximum convergence ratio:	0.148	0.190	0.186	0.140	0.248	0.224	0.159	0.237	0.167	0.173

Table 26: Results of the meta-analysis for negative network and PGG, Model 1

	est	se	N	p	tau2	Q	Qp
outdegree (density)	-2.121	0.147	20	0.000	0.202	18.790	0.470
reciprocity	0.583	0.081	20	0.000	0.002	18.129	0.514
transitive triplets	-0.099	0.026	20	0.000	0.052	22.214	0.274
indegree - popularity	0.115	0.014	20	0.000	0.001	16.158	0.647
outdegree - activity	0.096	0.009	20	0.000	0.017	22.467	0.262
indegree - activity	-0.021	0.043	5	0.626	0.000	2.176	0.703
outdegree-trunc(1)	-2.775	0.990	4	0.005	1.245	5.573	0.134
sex alter	0.017	0.082	20	0.837	0.191	31.816	0.033
sex ego	0.104	0.062	20	0.092	0.000	26.822	0.109
same sex	-0.511	0.058	20	0.000	0.001	26.792	0.110
grade alter	0.008	0.035	20	0.829	0.041	16.123	0.649
grade ego	0.036	0.041	20	0.375	0.090	24.820	0.167
grade similarity	-0.005	0.132	20	0.973	0.325	30.929	0.041
PGG alter	0.002	0.025	20	0.944	0.045	18.440	0.493
PGG ego	0.013	0.030	20	0.654	0.068	24.737	0.169
PGG similarity	-0.049	0.110	20	0.655	0.217	24.082	0.193
popularity alter	-0.134	0.021	20	0.000	0.002	25.447	0.146
popularity ego	-0.026	0.016	20	0.102	0.000	13.521	0.811
popularity ego x popularity alter	-0.015	0.005	20	0.002	0.000	13.987	0.784

Table 27: Goodness of fit statistics, Negative & PGG, Model 1

	Indegree distribution	Outdegree distribution	Geodesic Distance	Triad Census
1	0.716	0.396	0.922	0.732
2	0.975	0.807	0.952	0.835
3	0.626	0.974	1.000	0.663
4	0.195	0.322	0.194	0.090
5	0.333	0.160	0.065	0.970
6	0.969	0.020	0.020	0.997
7	0.014	0.777	0.149	0.737
8	0.936	0.488	0.071	0.970
9	0.877	0.425	0.080	0.904
10	0.852	0.007	0.769	0.780
11	0.677	0.831	0.883	0.711
12	0.188	0.545	0.869	0.990
13	0.988	0.108	0.037	0.513
14	0.394	0.216	0.594	0.947
15	0.981	0.118	0.284	0.291
16	0.692	0.692	0.078	0.757
17	0.985	0.833	0.971	1.000
18	0.025	0.403	0.344	0.522
19	0.983	0.800	0.426	0.706
20	0.440	0.543	0.946	0.746

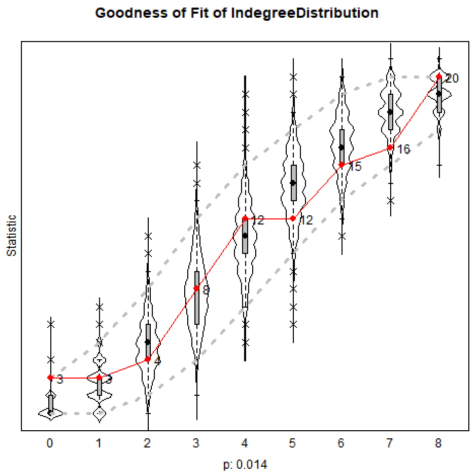

(a) Class 7

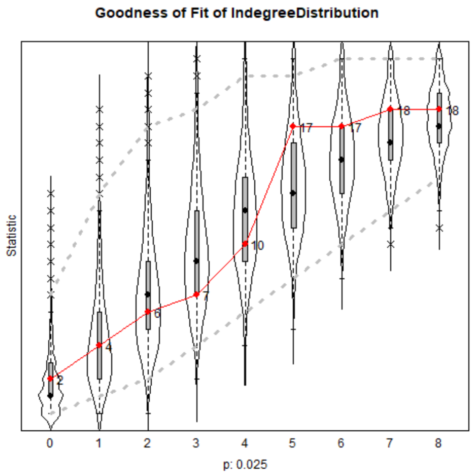

(b) Class 18

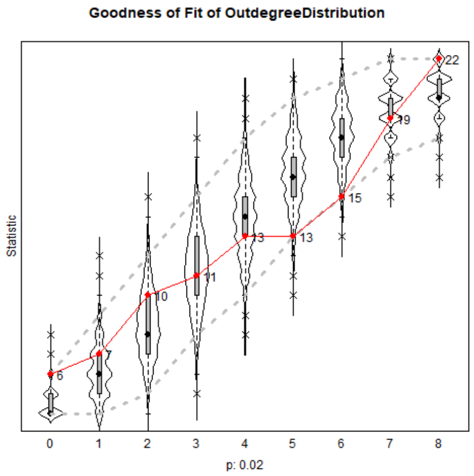

(c) Class 6

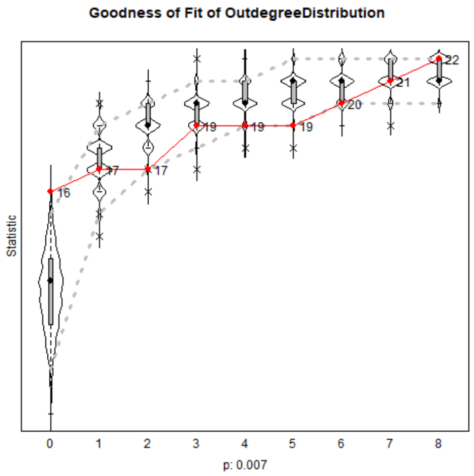

(d) Class 10

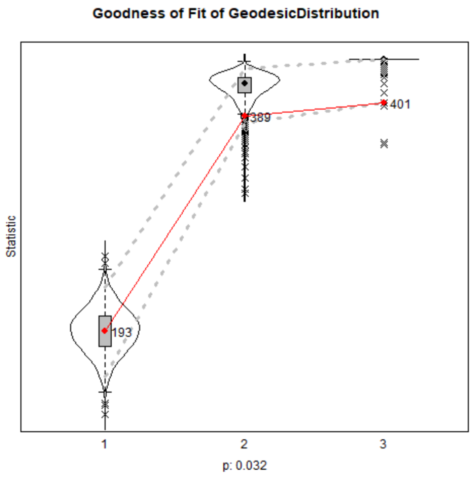

(e) Class 13

Figure 9: Classes with poor fit, Negative & PGG, Model 1

Table 28: Results of separate SAOMs for negative network and PGG, Model 1

	1	2	3	4	5	6	7	8	9	10
outdegree (density)	-1.952	2.595	0.079	-2.931	-2.347	-1.236	-2.207	-1.771	-1.548	-1.837
	[0.347]	[13.541]	[1.668]	[0.861]	[0.58]	[0.795]	[0.641]	[0.367]	[0.397]	[5.697]
reciprocity	0.699	2.286	0.004	0.431	1.112	0.63	1.115	0.429	0.299	0.074
	[0.224]	[1.378]	[0.498]	[0.52]	[0.292]	[0.47]	[0.408]	[0.212]	[0.266]	[0.971]
transitive triplets	-0.057	1.16	-0.161	0.396	-0.203	-0.001	-0.104	-0.247	0.037	0.006
	[0.05]	[1.203]	[0.166]	[0.268]	[0.08]	[0.158]	[0.084]	[0.062]	[0.069]	[0.904]
indegree - popularity	0.093	-0.096	-0.313	0.071	0.091	0.12	0.089	0.11	0.034	-1.177
	[0.036]	[0.5]	[0.279]	[0.122]	[0.068]	[0.047]	[0.065]	[0.045]	[0.052]	[0.775]
outdegree - activity	0.072	-1.468	0.14	0.058	0.152	0.051	0.117	0.098	0.034	0.264
	[0.019]	[4.484]	[0.055]	[0.073]	[0.028]	[0.049]	[0.035]	[0.02]	[0.027]	[0.147]
indegree - activity	0	0	0	0	0	-0.166	0	0	0	0.112
	[NA]	[NA]	[NA]	[NA]	[NA]	[0.119]	[NA]	[NA]	[NA]	[3.613]
outdegree-trunc(1)	0	0	0	1.349	0	0	0	-2.499	0	0
	[NA]	[NA]	[NA]	[2.14]	[NA]	[NA]	[NA]	[1.287]	[NA]	[NA]
sex alter	-0.019	-1.156	-1.967	0.111	-0.485	-0.166	0.087	0.309	0.249	-0.52
	[0.156]	[1.41]	[1.025]	[0.45]	[0.411]	[0.228]	[0.239]	[0.19]	[0.24]	[1.047]
sex ego	-0.027	3.629	-1.057	0.982	-0.341	-0.615	-0.031	0.063	0.34	-0.351
	[0.146]	[13.238]	[0.537]	[0.549]	[0.385]	[0.5]	[0.251]	[0.165]	[0.237]	[0.917]
same sex	-0.531	-1.346	-0.619	0.085	-1.4	-0.149	-0.405	-0.663	-0.501	-1.066
	[0.148]	[1.044]	[0.48]	[0.387]	[0.424]	[0.197]	[0.211]	[0.164]	[0.204]	[0.948]
grade alter	0.089	-0.375	-0.038	0.075	0.139	0.081	0.312	-0.179	-0.272	0.075
	[0.135]	[0.619]	[0.227]	[0.224]	[0.176]	[0.104]	[0.206]	[0.094]	[0.15]	[0.472]
grade ego	0.201	-1.575	0.369	-0.221	0.223	0.103	0.518	-0.152	-0.203	-0.796
	[0.142]	[5.225]	[0.246]	[0.243]	[0.157]	[0.111]	[0.247]	[0.092]	[0.149]	[1.19]
grade similarity	-0.033	-0.42	0.495	-0.475	-0.537	-0.253	-0.231	-0.598	0.135	3.154
	[0.248]	[1.763]	[0.481]	[0.772]	[0.883]	[0.432]	[0.33]	[0.305]	[0.301]	[1.673]
PGG alter	0.046	-0.137	0.154	0.06	-0.007	0.09	0.006	-0.024	0.025	0.063
	[0.065]	[0.923]	[0.313]	[0.167]	[0.113]	[0.08]	[0.151]	[0.077]	[0.094]	[0.38]
PGG ego	-0.107	-6.237	0.445	0.144	-0.145	0.182	0.067	-0.02	-0.02	0.055
	[0.07]	[18.487]	[0.267]	[0.153]	[0.122]	[0.151]	[0.165]	[0.066]	[0.086]	[0.676]
PGG similarity	-0.139	0.918	0.702	1.753	-0.564	0.746	0.563	0.097	-0.385	0.957
	[0.285]	[1.926]	[0.731]	[0.858]	[0.432]	[0.427]	[0.527]	[0.363]	[0.304]	[2.957]
popularity alter	-0.098	-0.07	-0.811	-0.235	-0.263	-0.061	-0.343	-0.167	-0.139	-1.36
	[0.053]	[0.443]	[0.416]	[0.169]	[0.103]	[0.074]	[0.128]	[0.058]	[0.083]	[0.597]
popularity ego	0.016	-3.663	-0.267	0.072	-0.06	-0.248	-0.01	-0.03	-0.105	0.293
	[0.034]	[10.815]	[0.134]	[0.12]	[0.068]	[0.184]	[0.105]	[0.04]	[0.048]	[2.005]
popularity ego x popularity alter	-0.023	0.131	0.013	-0.114	0.019	-0.017	0.013	-0.016	0.004	0.05
	[0.008]	[0.367]	[0.031]	[0.057]	[0.034]	[0.02]	[0.034]	[0.015]	[0.017]	[0.095]
Overall maximum convergence ratio:	0.108	0.154	0.161	0.147	0.119	0.172	0.126	0.210	0.098	0.208

	11	12	13	14	15	16	17	18	19	20
outdegree (density)	-1.624	-2.96	-1.653	-2.968	-3.063	-2.566	-2.163	-2.328	-0.398	-1.749
	[0.696]	[0.6]	[0.835]	[0.492]	[0.534]	[1.067]	[0.838]	[0.401]	[1.252]	[0.784]
reciprocity	0.484	0.988	0.843	0.684	-0.102	0.427	0.453	0.672	-0.829	-0.038
	[0.36]	[0.345]	[0.326]	[0.27]	[0.413]	[0.303]	[0.895]	[0.446]	[1.203]	[0.547]
transitive triplets	-0.22	-0.053	0.006	-0.117	-0.241	-0.116	-0.744	-0.025	-0.336	-0.272
	[0.115]	[0.065]	[0.115]	[0.059]	[0.184]	[0.09]	[0.808]	[0.094]	[0.532]	[0.223]
indegree - popularity	0.112	0.11	0.094	0.168	0.189	0.12	-0.127	0.168	-0.102	0.032
	[0.073]	[0.068]	[0.058]	[0.041]	[0.056]	[0.053]	[0.201]	[0.042]	[0.25]	[0.187]
outdegree - activity	0.091	0.103	0.066	0.109	0.171	0.102	0.033	0.078	0.157	0.211
	[0.047]	[0.026]	[0.037]	[0.026]	[0.049]	[0.028]	[0.069]	[0.034]	[0.131]	[0.065]
indegree - activity	0	0	-0.064	0	0	0.013	0	0.017	0	0
	[NA]	[NA]	[0.108]	[NA]	[NA]	[0.108]	[NA]	[0.058]	[NA]	[NA]
outdegree-trunc(1)	-4.164	0	0	0	0	0	0	0	-4.174	0
	[1.333]	[NA]	[NA]	[NA]	[NA]	[NA]	[NA]	[NA]	[1.599]	[NA]
sex alter	-0.382	-0.291	-0.227	0.272	1.262	0.022	-0.509	0.371	-1.407	-0.68
	[0.251]	[0.24]	[0.334]	[0.184]	[0.42]	[0.233]	[0.737]	[0.228]	[1.209]	[0.504]
sex ego	-0.104	0.119	0.079	0.25	0.808	0.384	1.686	0.414	-0.837	-0.219
	[0.237]	[0.221]	[0.293]	[0.193]	[0.333]	[0.298]	[0.954]	[0.234]	[0.688]	[0.403]
same sex	-0.336	-0.314	-0.448	-0.427	-1.036	-0.5	0.43	-0.99	-1.59	-1.539
	[0.249]	[0.227]	[0.286]	[0.201]	[0.344]	[0.191]	[0.591]	[0.307]	[0.706]	[0.56]
grade alter	0.014	0.042	0.109	0.015	0.041	0.053	-0.007	-0.181	0.19	0.279
	[0.121]	[0.187]	[0.11]	[0.104]	[0.129]	[0.119]	[0.23]	[0.129]	[0.458]	[0.249]
grade ego	0.018	-0.095	-0.115	0.141	-0.035	0.241	-0.017	0.044	-0.078	0.111
	[0.129]	[0.173]	[0.097]	[0.114]	[0.115]	[0.118]	[0.193]	[0.145]	[0.231]	[0.206]
grade similarity	-0.692	-0.661	-0.154	0.794	1.425	0.734	0.98	0.057	-1.408	0.268
	[0.449]	[0.405]	[0.419]	[0.432]	[0.651]	[0.378]	[0.992]	[0.392]	[0.897]	[0.77]
PGG alter	0.166	-0.075	0.024	-0.041	-0.042	0.037	-0.404	-0.14	0.383	-0.219
	[0.077]	[0.09]	[0.085]	[0.072]	[0.098]	[0.068]	[0.262]	[0.059]	[0.329]	[0.289]
PGG ego	0.138	-0.168	0.107	-0.151	-0.001	0.18	0.013	0.022	0.157	-0.08
	[0.082]	[0.108]	[0.088]	[0.083]	[0.089]	[0.084]	[0.202]	[0.055]	[0.199]	[0.214]
PGG similarity	-0.456	-0.321	0.066	0.519	-0.459	-0.23	-0.656	-0.349	0.221	1.109
	[0.293]	[0.445]	[0.411]	[0.303]	[0.453]	[0.336]	[0.708]	[0.248]	[1.142]	[0.996]
popularity alter	-0.27	-0.116	-0.106	-0.05	-0.062	-0.224	-0.28	0.002	-0.718	-0.489
	[0.125]	[0.063]	[0.095]	[0.066]	[0.116]	[0.088]	[0.195]	[0.08]	[0.407]	[0.237]
popularity ego	-0.064	-0.001	0.06	-0.001	-0.02	0.1	-0.098	0.095	-0.003	-0.112
	[0.063]	[0.045]	[0.136]	[0.059]	[0.087]	[0.213]	[0.115]	[0.12]	[0.116]	[0.134]
popularity ego x popularity alter	-0.019	-0.006	-0.019	-0.036	-0.024	0.024	-0.033	-0.026	-0.028	-0.086
	[0.021]	[0.014]	[0.033]	[0.023]	[0.037]	[0.027]	[0.065]	[0.036]	[0.046]	[0.052]
Overall maximum convergence ratio:	0.136	0.133	0.126	0.120	0.134	0.132	0.110	0.136	0.214	0.141

Table 29: Results of the meta-analysis for negative network and PGG, Model 2

	est	se	N	p	tau2	Q	Qp
outdegree (density)	-2.166	0.1496	20	0.000	0.199	18.300	0.502
reciprocity	0.609	0.0801	20	0.000	0.001	17.774	0.538
transitive triplets	-0.100	0.0272	20	0.000	0.057	23.570	0.213
indegree - popularity	0.118	0.0137	20	0.000	0.000	16.338	0.635
outdegree - activity	0.097	0.0092	20	0.000	0.017	22.067	0.281
indegree - activity	-0.018	0.0439	5	0.678	0.000	1.733	0.785
outdegree-trunc(1)	-2.555	1.1216	4	0.023	1.586	6.153	0.104
sex alter	0.026	0.0829	20	0.758	0.198	32.356	0.028
sex ego	0.115	0.0681	20	0.090	0.099	30.268	0.048
same sex	-0.514	0.0577	20	0.000	0.001	27.246	0.099
grade alter	0.005	0.0346	20	0.875	0.037	16.171	0.646
grade ego	0.040	0.0410	20	0.332	0.089	24.703	0.171
grade similarity	-0.004	0.1258	20	0.977	0.280	28.038	0.083
PGG alter	-0.037	0.0306	20	0.224	0.000	10.914	0.927
PGG ego	0.048	0.0320	20	0.135	0.071	24.542	0.176
PGG similarity	-0.096	0.1134	20	0.395	0.225	25.091	0.158
popularity alter	-0.132	0.0210	20	0.000	0.001	25.377	0.149
popularity ego	-0.025	0.0161	20	0.118	0.000	14.081	0.779
popularity ego x popularity alter	-0.016	0.0049	20	0.001	0.000	14.358	0.762
int. PGG ego x PGG similarity	0.238	0.0951	20	0.012	0.000	20.549	0.362

Table 30: Goodness of fit statistics, Negative & PGG, Model 2

	Indegree distribution	Outdegree distribution	Geodesic Distance	Triad Census
1	0.727	0.415	0.935	0.717
2	1.000	0.813	0.962	0.848
3	0.661	0.970	1.000	0.672
4	0.278	0.355	0.186	0.096
5	0.303	0.146	0.056	0.967
6	0.971	0.034	0.015	0.997
7	0.016	0.745	0.187	0.759
8	0.950	0.518	0.072	0.982
9	0.898	0.454	0.091	0.884
10	0.898	0.009	0.804	0.807
11	0.655	0.804	0.901	0.694
12	0.209	0.479	0.866	0.997
13	0.988	0.069	0.042	0.519
14	0.384	0.232	0.657	0.928
15	0.983	0.099	0.361	0.278
16	0.688	0.746	0.072	0.792
17	0.989	0.883	0.990	1.000
18	0.046	0.327	0.235	0.583
19	0.993	0.818	0.532	0.820
20	0.502	0.685	0.935	0.578

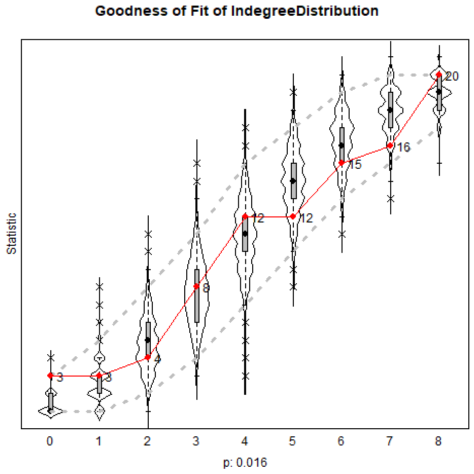

(a) Class 7

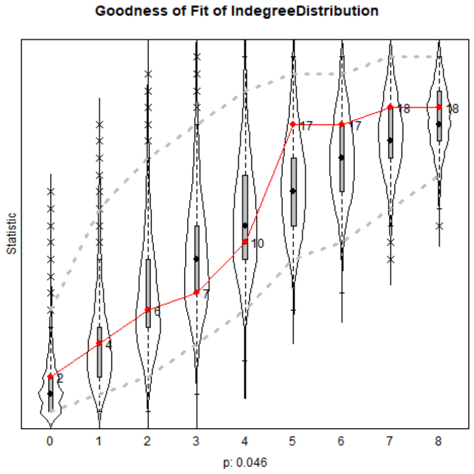

(b) Class 18

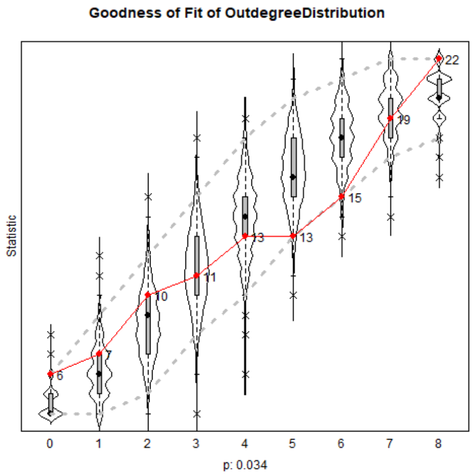

(c) Class 6

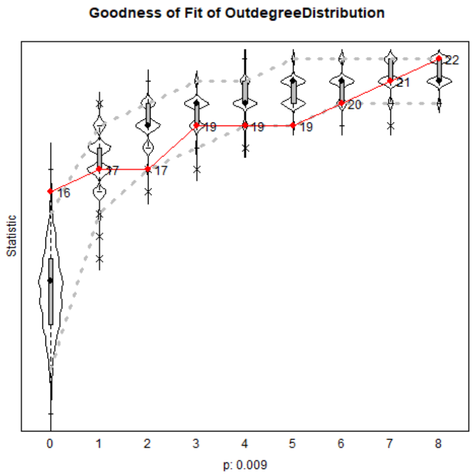

(d) Class 10

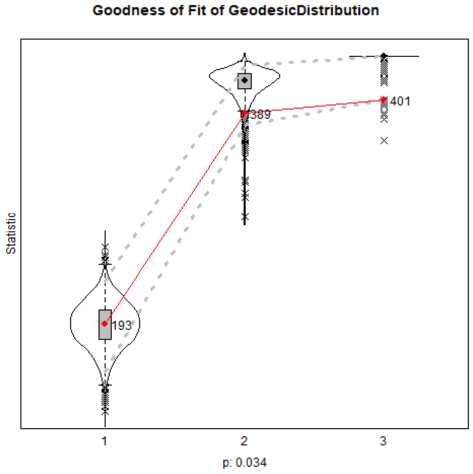

(e) Class 13

Figure 10: Classes with poor fit, Negative & PGG, Model 2

Table 31: Results of separate SAOMs for negative network and PGG, Model 2

	1	2	3	4	5	6	7	8	9	10
outdegree (density)	-1.968	2.376	0.246	-2.922	-2.376	-1.257	-2.122	-1.845	-1.535	-1.596
	[0.37]	[9.995]	[1.612]	[0.907]	[0.586]	[0.959]	[0.668]	[0.413]	[0.411]	[3.337]
reciprocity	0.718	2.533	0.005	0.458	1.135	0.623	1.172	0.502	0.301	0.288
	[0.223]	[1.641]	[0.492]	[0.54]	[0.301]	[0.416]	[0.38]	[0.219]	[0.278]	[1.006]
transitive triplets	-0.058	1.159	-0.163	0.382	-0.2	0.001	-0.099	-0.266	0.043	0.09
	[0.051]	[1.361]	[0.143]	[0.235]	[0.078]	[0.174]	[0.086]	[0.066]	[0.073]	[0.7]
indegree - popularity	0.094	-0.076	-0.308	0.07	0.085	0.12	0.094	0.123	0.032	-1.112
	[0.038]	[0.472]	[0.272]	[0.116]	[0.072]	[0.044]	[0.073]	[0.045]	[0.055]	[0.729]
outdegree - activity	0.073	-1.5	0.137	0.062	0.156	0.052	0.109	0.106	0.035	0.261
	[0.019]	[3.475]	[0.054]	[0.073]	[0.028]	[0.052]	[0.034]	[0.023]	[0.027]	[0.147]
indegree - activity	0	0	0	0	0	-0.164	0	0	0	-0.295
	[NA]	[NA]	[NA]	[NA]	[NA]	[0.134]	[NA]	[NA]	[NA]	[2.185]
outdegree-trunc(1)	0	0	0	1.33	0	0	0	-2.267	0	0
	[NA]	[NA]	[NA]	[1.999]	[NA]	[NA]	[NA]	[1.218]	[NA]	[NA]
sex alter	-0.012	-1.067	-1.959	0.111	-0.472	-0.161	0.125	0.371	0.239	-0.515
	[0.147]	[1.438]	[0.997]	[0.465]	[0.394]	[0.244]	[0.221]	[0.215]	[0.235]	[0.893]
sex ego	-0.028	3.689	-1.177	1.007	-0.328	-0.609	0.018	0.042	0.343	-0.488
	[0.156]	[10.643]	[0.533]	[0.542]	[0.365]	[0.484]	[0.266]	[0.167]	[0.24]	[0.971]
same sex	-0.532	-1.335	-0.654	0.077	-1.402	-0.154	-0.427	-0.675	-0.497	-1.175
	[0.158]	[1.113]	[0.432]	[0.393]	[0.398]	[0.204]	[0.208]	[0.167]	[0.204]	[1.126]
grade alter	0.089	-0.493	-0.059	0.074	0.14	0.077	0.27	-0.169	-0.27	0.098
	[0.133]	[0.688]	[0.237]	[0.224]	[0.173]	[0.103]	[0.19]	[0.094]	[0.149]	[0.49]
grade ego	0.205	-1.569	0.372	-0.217	0.222	0.096	0.553	-0.163	-0.199	-0.75
	[0.144]	[4.669]	[0.233]	[0.23]	[0.159]	[0.119]	[0.258]	[0.101]	[0.15]	[1.192]
grade similarity	-0.03	-0.323	0.519	-0.486	-0.503	-0.248	-0.21	-0.584	0.137	3.277
	[0.255]	[1.681]	[0.459]	[0.743]	[0.878]	[0.427]	[0.334]	[0.319]	[0.308]	[1.906]
PGG alter	0.03	0.7	-0.057	0.012	-0.094	0.087	-0.14	-0.169	-0.062	0.406
	[0.078]	[1.801]	[0.389]	[0.217]	[0.134]	[0.107]	[0.163]	[0.088]	[0.145]	[0.575]
PGG ego	-0.09	-6.041	0.621	0.155	-0.073	0.182	0.321	0.165	0	-0.199
	[0.082]	[15.39]	[0.309]	[0.157]	[0.134]	[0.141]	[0.225]	[0.093]	[0.092]	[0.5]
PGG similarity	-0.173	0.961	0.348	1.735	-0.686	0.749	0.272	-0.25	-0.445	1.991
	[0.293]	[1.758]	[0.819]	[0.856]	[0.464]	[0.407]	[0.519]	[0.352]	[0.314]	[2.393]
popularity alter	-0.098	-0.088	-0.808	-0.237	-0.269	-0.06	-0.33	-0.167	-0.14	-1.334
	[0.055]	[0.447]	[0.417]	[0.154]	[0.111]	[0.073]	[0.159]	[0.057]	[0.084]	[0.569]
popularity ego	0.017	-3.718	-0.28	0.073	-0.051	-0.245	-0.052	-0.037	-0.102	0.013
	[0.034]	[8.543]	[0.126]	[0.125]	[0.069]	[0.191]	[0.097]	[0.04]	[0.049]	[1.561]
popularity ego x popularity alter	-0.024	0.123	0.01	-0.11	0.017	-0.018	0.019	-0.014	0.003	0.046
	[0.008]	[0.379]	[0.036]	[0.052]	[0.035]	[0.019]	[0.038]	[0.015]	[0.017]	[0.102]
int. PGG ego x PGG similarity	0.105	-4.686	0.726	0.253	0.57	0	0.962	0.982	0.267	-1.354
	[0.278]	[9.916]	[0.63]	[0.799]	[0.485]	[0.366]	[0.582]	[0.325]	[0.365]	[1.295]
Overall maximum convergence ratio:	0.145	0.155	0.215	0.162	0.135	0.139	0.167	0.145	0.117	0.194

	11	12	13	14	15	16	17	18	19	20
outdegree (density)	-1.603	-2.87	-1.662	-2.989	-3.062	-2.55	-2.292	-2.322	-0.343	-1.973
	[0.694]	[0.556]	[0.833]	[0.497]	[0.498]	[0.982]	[0.894]	[0.399]	[1.333]	[0.889]
reciprocity	0.502	1.019	0.83	0.685	-0.086	0.436	0.442	0.665	-0.751	0.032
	[0.342]	[0.332]	[0.326]	[0.276]	[0.404]	[0.282]	[0.841]	[0.427]	[1.229]	[0.581]
transitive triplets	-0.213	-0.05	0.003	-0.117	-0.232	-0.116	-0.726	-0.024	-0.329	-0.298
	[0.092]	[0.07]	[0.114]	[0.06]	[0.184]	[0.089]	[0.974]	[0.086]	[0.505]	[0.244]
indegree - popularity	0.11	0.109	0.095	0.169	0.19	0.12	-0.103	0.168	-0.123	0.05
	[0.072]	[0.063]	[0.055]	[0.041]	[0.055]	[0.051]	[0.192]	[0.04]	[0.245]	[0.19]
outdegree - activity	0.088	0.1	0.065	0.109	0.17	0.101	0.032	0.078	0.159	0.22
	[0.041]	[0.027]	[0.037]	[0.028]	[0.045]	[0.028]	[0.079]	[0.032]	[0.115]	[0.075]
indegree - activity	0	0	-0.059	0	0	0.01	0	0.015	0	0
	[NA]	[NA]	[0.105]	[NA]	[NA]	[0.101]	[NA]	[0.06]	[NA]	[NA]
outdegree-trunc(1)	-4.156	0	0	0	0	0	0	0	-4.418	0
	[1.346]	[NA]	[NA]	[NA]	[NA]	[NA]	[NA]	[NA]	[1.929]	[NA]
sex alter	-0.347	-0.284	-0.215	0.265	1.241	0.035	-0.46	0.37	-1.387	-0.783
	[0.244]	[0.226]	[0.334]	[0.187]	[0.405]	[0.238]	[0.72]	[0.22]	[1.229]	[0.561]
sex ego	-0.121	0.101	0.101	0.235	0.802	0.377	1.749	0.419	-0.817	-0.304
	[0.235]	[0.228]	[0.285]	[0.191]	[0.312]	[0.254]	[0.907]	[0.211]	[0.688]	[0.44]
same sex	-0.31	-0.337	-0.446	-0.423	-1.021	-0.492	0.421	-0.976	-1.598	-1.54
	[0.23]	[0.217]	[0.275]	[0.198]	[0.332]	[0.2]	[0.561]	[0.29]	[0.782]	[0.551]
grade alter	0.009	0.027	0.107	0.01	0.043	0.046	0.007	-0.183	0.213	0.347
	[0.119]	[0.183]	[0.113]	[0.108]	[0.129]	[0.12]	[0.237]	[0.125]	[0.497]	[0.267]
grade ego	0.015	-0.075	-0.121	0.146	-0.032	0.223	-0.033	0.056	-0.094	0.2
	[0.133]	[0.18]	[0.104]	[0.113]	[0.116]	[0.119]	[0.218]	[0.132]	[0.236]	[0.21]
grade similarity	-0.642	-0.664	-0.159	0.817	1.496	0.75	0.854	0.085	-1.438	0.183
	[0.436]	[0.406]	[0.41]	[0.443]	[0.704]	[0.406]	[0.998]	[0.394]	[1.125]	[0.737]
PGG alter	0.052	-0.167	0.022	-0.075	-0.154	-0.026	-0.241	-0.006	0.439	0.133
	[0.115]	[0.126]	[0.11]	[0.116]	[0.157]	[0.098]	[0.285]	[0.148]	[0.359]	[0.4]
PGG ego	0.153	-0.118	0.109	-0.145	0.017	0.198	-0.029	0.013	0.109	-0.065
	[0.081]	[0.118]	[0.099]	[0.09]	[0.095]	[0.087]	[0.22]	[0.059]	[0.26]	[0.265]
PGG similarity	-0.537	-0.467	0.072	0.531	-0.578	-0.188	-0.316	-0.339	0.047	1.614
	[0.328]	[0.444]	[0.403]	[0.318]	[0.462]	[0.319]	[0.851]	[0.253]	[1.412]	[1.082]
popularity alter	-0.269	-0.118	-0.102	-0.048	-0.06	-0.222	-0.272	-0.003	-0.762	-0.498
	[0.115]	[0.059]	[0.097]	[0.066]	[0.12]	[0.087]	[0.18]	[0.075]	[0.41]	[0.237]
popularity ego	-0.065	0.002	0.068	0	-0.019	0.099	-0.086	0.092	0.016	-0.127
	[0.072]	[0.044]	[0.146]	[0.06]	[0.086]	[0.211]	[0.117]	[0.109]	[0.123]	[0.156]
popularity ego x popularity alter	-0.023	-0.004	-0.019	-0.038	-0.024	0.024	-0.031	-0.027	-0.024	-0.101
	[0.021]	[0.014]	[0.031]	[0.023]	[0.038]	[0.028]	[0.072]	[0.036]	[0.044]	[0.058]
int. PGG ego x PGG similarity	0.389	0.431	0.013	0.112	0.426	0.247	-0.748	-0.357	-0.384	-1.892
	[0.301]	[0.382]	[0.428]	[0.32]	[0.477]	[0.286]	[0.719]	[0.367]	[0.881]	[1.043]
Overall maximum convergence ratio:	0.155	0.166	0.148	0.094	0.165	0.170	0.137	0.143	0.157	0.165

Table 32: Results of the meta-analysis for negative network and DG, Model 1

	est	se	N	p	tau2	Q	Qp
outdegree (density)	-2.165	0.1536	20	0.000	0.2434	19.936	0.398
reciprocity	0.594	0.0783	20	0.000	0.0000	16.767	0.606
transitive triplets	-0.106	0.0262	20	0.000	0.0483	21.924	0.288
indegree - popularity	0.120	0.0136	20	0.000	0.0016	20.204	0.382
outdegree - activity	0.099	0.0094	20	0.000	0.0184	24.713	0.170
indegree - activity	-0.011	0.0453	5	0.814	0.0000	1.627	0.804
outdegree-trunc(1)	-2.887	1.0315	4	0.005	1.2884	5.508	0.138
sex alter	0.025	0.0729	20	0.737	0.1311	34.714	0.015
sex ego	0.166	0.0625	20	0.008	0.0000	23.010	0.237
same sex	-0.505	0.0587	20	0.000	0.0004	25.987	0.131
grade alter	0.015	0.0370	20	0.682	0.0628	20.632	0.357
grade ego	-0.004	0.0359	20	0.905	0.0464	16.746	0.607
grade similarity	0.014	0.1300	20	0.913	0.3011	28.379	0.076
DG alter	-0.007	0.0167	20	0.688	0.0251	21.209	0.325
DG ego	0.002	0.0210	20	0.937	0.0467	26.018	0.130
DG similarity	-0.201	0.0829	20	0.015	0.0161	26.436	0.118
popularity alter	-0.138	0.0231	20	0.000	0.0324	25.162	0.155
popularity ego	-0.003	0.0151	20	0.852	0.0000	11.102	0.920
popularity ego x popularity alter	-0.017	0.0049	20	0.000	0.0000	12.882	0.845

Table 33: Goodness of fit statistics, Negative & DG, Model 1

	Indegree distribution	Outdegree distribution	Geodesic Distance	Triad Census
1	0.764	0.374	0.937	0.694
2	0.912	0.944	0.981	0.876
3	0.649	0.935	1.000	0.729
4	0.349	0.324	0.258	0.130
5	0.316	0.160	0.034	0.978
6	0.949	0.052	0.014	0.998
7	0.060	0.818	0.612	0.928
8	0.924	0.518	0.062	0.982
9	0.878	0.474	0.098	0.893
10	0.904	0.006	0.764	0.845
11	0.533	0.791	0.900	0.791
12	0.222	0.488	0.877	0.992
13	0.994	0.226	0.040	0.618
14	0.413	0.202	0.402	0.940
15	0.983	0.186	0.334	0.286
16	0.738	0.798	0.048	0.753
17	0.989	0.863	0.918	1.000
18	0.021	0.387	0.478	0.498
19	0.986	0.760	0.230	0.619
20	0.475	0.613	0.949	0.708

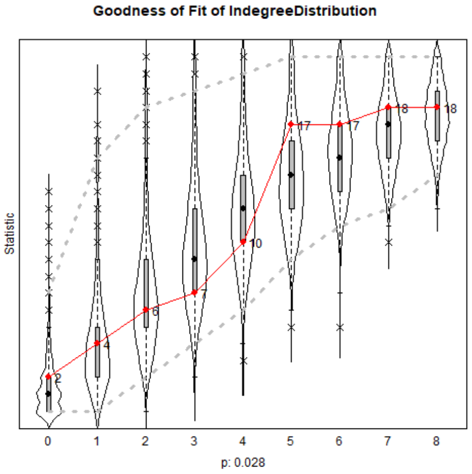

(a) Class 18

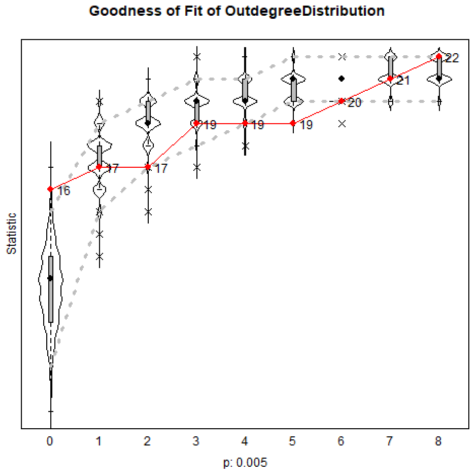

(b) Class 10

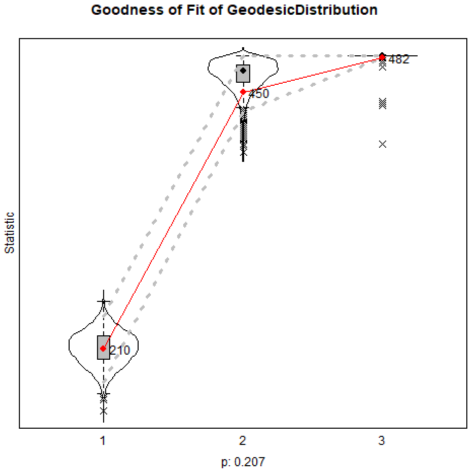

(c) Class 5

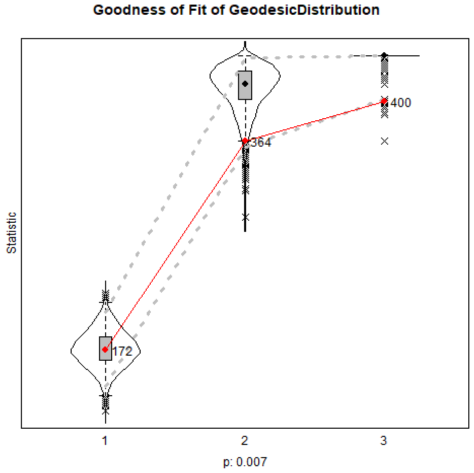

(d) Class 6

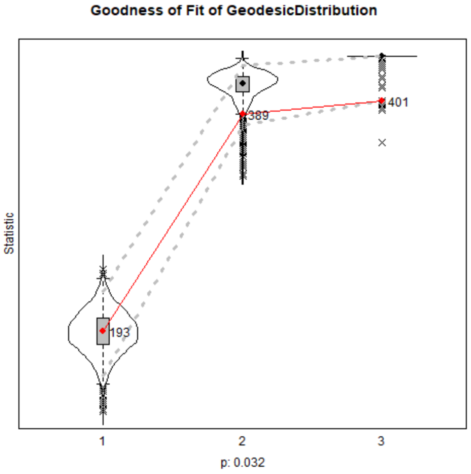

(e) Class 13

Figure 11: Classes with poor fit, Negative & DG, Model 1

Table 34: Results of separate SAOMs for negative network and DG, Model 1

	1	2	3	4	5	6	7	8	9	10
outdegree (density)	-2.043	5.324	-0.565	-2.833	-2.295	-0.831	-2.529	-1.611	-1.535	-1.443
	[0.345]	[7.555]	[1.431]	[0.9]	[0.595]	[1.513]	[0.673]	[0.397]	[0.423]	[4.636]
reciprocity	0.68	1.779	0.101	0.54	1.102	0.65	1.195	0.562	0.336	0.112
	[0.215]	[1.116]	[0.459]	[0.486]	[0.301]	[0.407]	[0.421]	[0.213]	[0.259]	[1.103]
transitive triplets	-0.063	1.072	-0.193	0.328	-0.201	0.033	-0.156	-0.233	0.037	0.119
	[0.05]	[1.156]	[0.165]	[0.253]	[0.079]	[0.169]	[0.115]	[0.065]	[0.073]	[0.53]
indegree - popularity	0.103	-0.949	-0.304	0.075	0.083	0.129	0.122	0.075	0.024	-1.226
	[0.037]	[0.689]	[0.26]	[0.118]	[0.07]	[0.038]	[0.082]	[0.048]	[0.056]	[0.891]
outdegree - activity	0.076	-1.943	0.167	0.07	0.152	0.028	0.158	0.095	0.036	0.252
	[0.017]	[2.461]	[0.053]	[0.075]	[0.028]	[0.071]	[0.052]	[0.021]	[0.026]	[0.144]
indegree - activity	0	0	0	0	0	-0.229	0	0	0	-0.148
	[NA]	[NA]	[NA]	[NA]	[NA]	[0.218]	[NA]	[NA]	[NA]	[3.104]
outdegree-trunc(1)	0	0	0	1.211	0	0	0	-2.321	0	0
	[NA]	[NA]	[NA]	[2.291]	[NA]	[NA]	[NA]	[1.222]	[NA]	[NA]
sex alter	-0.03	-3.259	-1.894	0.007	-0.428	-0.07	-0.024	0.227	0.225	-0.441
	[0.153]	[1.873]	[0.807]	[0.426]	[0.391]	[0.263]	[0.291]	[0.195]	[0.22]	[1.032]
sex ego	-0.013	-0.543	-0.237	0.887	-0.372	-0.528	-0.264	0.163	0.36	-0.44
	[0.154]	[2.082]	[0.382]	[0.503]	[0.365]	[0.622]	[0.333]	[0.162]	[0.236]	[0.93]
same sex	-0.541	-1.426	-0.481	0.081	-1.398	-0.179	-0.501	-0.659	-0.51	-1.305
	[0.157]	[0.988]	[0.485]	[0.389]	[0.422]	[0.204]	[0.26]	[0.166]	[0.21]	[0.787]
grade alter	0.114	-0.445	-0.006	0.157	0.185	0.053	0.363	-0.265	-0.266	0.132
	[0.137]	[0.478]	[0.233]	[0.213]	[0.18]	[0.097]	[0.206]	[0.108]	[0.145]	[0.53]
grade ego	0.141	1.185	-0.075	-0.197	0.217	0.105	0.78	-0.101	-0.198	-0.708
	[0.14]	[1.388]	[0.189]	[0.212]	[0.16]	[0.116]	[0.688]	[0.096]	[0.153]	[1.327]
grade similarity	-0.011	-0.284	0.674	-0.348	-0.232	-0.198	-0.176	-0.661	0.174	3.178
	[0.259]	[1.377]	[0.465]	[0.725]	[0.916]	[0.446]	[0.371]	[0.289]	[0.29]	[2.011]
DG alter	0.029	-0.75	0.087	0.037	-0.137	-0.007	-0.079	-0.104	0.019	0.211
	[0.044]	[0.415]	[0.139]	[0.136]	[0.115]	[0.047]	[0.099]	[0.057]	[0.055]	[0.25]
DG ego	-0.068	-1.394	-0.217	0.088	-0.144	0.056	0.439	0.076	-0.084	0.016
	[0.046]	[1.76]	[0.13]	[0.119]	[0.13]	[0.083]	[0.391]	[0.045]	[0.059]	[0.337]
DG similarity	-0.144	-1.27	-0.428	0.14	0.122	-0.472	1.78	-0.054	0.012	2.248
	[0.268]	[1.508]	[0.51]	[0.721]	[0.557]	[0.29]	[1.051]	[0.251]	[0.321]	[1.495]
popularity alter	-0.1	-0.272	-0.794	-0.252	-0.266	-0.05	-0.26	-0.202	-0.157	-1.43
	[0.055]	[0.438]	[0.363]	[0.158]	[0.1]	[0.073]	[0.13]	[0.065]	[0.091]	[0.675]
popularity ego	0.027	-2.26	-0.106	0.07	-0.03	-0.331	-0.182	-0.024	-0.081	0.117
	[0.03]	[2.948]	[0.096]	[0.117]	[0.068]	[0.322]	[0.303]	[0.039]	[0.05]	[1.99]
popularity ego x popularity alter	-0.026	0.192	0.004	-0.108	0.018	-0.017	-0.023	-0.017	0.003	0.071
	[0.009]	[0.346]	[0.035]	[0.049]	[0.035]	[0.02]	[0.035]	[0.016]	[0.016]	[0.11]
Overall maximum convergence ratio:	0.115	0.130	0.161	0.143	0.125	0.174	0.112	0.185	0.105	0.133

	11	12	13	14	15	16	17	18	19	20
outdegree (density)	-1.994	-3.041	-1.596	-2.686	-3.2	-2.564	-2.475	-2.362	-0.497	-1.647
	[0.585]	[0.552]	[0.976]	[0.447]	[0.547]	[1.078]	[0.877]	[0.411]	[0.959]	[0.895]
reciprocity	0.509	0.982	0.788	0.629	-0.06	0.379	0.059	0.643	-1.194	-0.021
	[0.331]	[0.335]	[0.306]	[0.269]	[0.378]	[0.322]	[0.838]	[0.389]	[1.613]	[0.539]
transitive triplets	-0.233	-0.039	-0.003	-0.112	-0.254	-0.119	-0.664	-0.048	-0.678	-0.313
	[0.093]	[0.068]	[0.128]	[0.061]	[0.184]	[0.082]	[0.762]	[0.1]	[0.59]	[0.235]
indegree - popularity	0.157	0.109	0.095	0.165	0.203	0.117	-0.062	0.182	-0.055	0
	[0.061]	[0.063]	[0.059]	[0.042]	[0.057]	[0.048]	[0.176]	[0.041]	[0.199]	[0.207]
outdegree - activity	0.097	0.102	0.069	0.093	0.177	0.11	0.035	0.082	0.203	0.221
	[0.038]	[0.025]	[0.042]	[0.025]	[0.051]	[0.027]	[0.076]	[0.034]	[0.138]	[0.067]
indegree - activity	0	0	-0.08	0	0	0.003	0	0.017	0	0
	[NA]	[NA]	[0.116]	[NA]	[NA]	[0.125]	[NA]	[0.055]	[NA]	[NA]
outdegree-trunc(1)	-4.534	0	0	0	0	0	0	0	-4.468	0
	[1.457]	[NA]	[NA]	[NA]	[NA]	[NA]	[NA]	[NA]	[1.739]	[NA]
sex alter	-0.119	-0.347	-0.18	0.241	1.229	0.065	-1.327	0.413	-2.241	-0.561
	[0.217]	[0.26]	[0.28]	[0.196]	[0.394]	[0.23]	[0.784]	[0.235]	[1.743]	[0.519]
sex ego	0.091	0.269	0.148	0.216	0.786	0.387	1.826	0.497	-1.029	-0.051
	[0.234]	[0.256]	[0.249]	[0.187]	[0.314]	[0.29]	[0.923]	[0.242]	[0.857]	[0.384]
same sex	-0.275	-0.242	-0.456	-0.419	-1.029	-0.493	0.443	-0.997	-1.392	-1.558
	[0.218]	[0.22]	[0.287]	[0.189]	[0.352]	[0.194]	[0.65]	[0.319]	[0.793]	[0.599]
grade alter	0.039	0.073	0.127	-0.026	0.038	0.037	-0.002	-0.085	0.59	0.245
	[0.113]	[0.202]	[0.115]	[0.106]	[0.129]	[0.112]	[0.248]	[0.112]	[0.502]	[0.236]
grade ego	0.007	-0.106	-0.146	0.004	-0.043	0.221	-0.094	-0.024	-0.031	0.087
	[0.126]	[0.187]	[0.097]	[0.119]	[0.121]	[0.111]	[0.246]	[0.146]	[0.25]	[0.214]
grade similarity	-0.494	-0.727	-0.223	0.922	1.385	0.586	1.348	0.181	-1.377	0.015
	[0.43]	[0.436]	[0.45]	[0.494]	[0.707]	[0.388]	[1.055]	[0.397]	[1.072]	[0.698]
DG alter	-0.021	-0.002	0.069	0.044	0.071	0.036	-0.142	-0.092	0.08	0.19
	[0.055]	[0.058]	[0.06]	[0.053]	[0.109]	[0.048]	[0.111]	[0.042]	[0.172]	[0.175]
DG ego	-0.041	-0.085	0.106	0.095	0.224	-0.038	0.027	-0.015	0.068	0.073
	[0.058]	[0.067]	[0.076]	[0.058]	[0.115]	[0.044]	[0.123]	[0.048]	[0.105]	[0.165]
DG similarity	-0.003	-0.667	-0.21	-0.012	-1.079	-0.336	-1.762	0.252	-0.703	-0.107
	[0.297]	[0.339]	[0.301]	[0.3]	[0.638]	[0.252]	[0.538]	[0.27]	[0.48]	[0.749]
popularity alter	-0.221	-0.092	-0.125	-0.043	-0.054	-0.246	-0.189	-0.015	-0.847	-0.391
	[0.1]	[0.061]	[0.1]	[0.061]	[0.124]	[0.091]	[0.164]	[0.078]	[0.462]	[0.194]
popularity ego	-0.059	0.031	0.055	0.029	0.022	0.046	-0.051	0.126	0.015	-0.049
	[0.065]	[0.04]	[0.158]	[0.05]	[0.084]	[0.234]	[0.122]	[0.12]	[0.123]	[0.093]
popularity ego x popularity alter	-0.019	-0.011	-0.018	-0.027	-0.017	0.028	-0.059	-0.026	-0.02	-0.058
	[0.02]	[0.014]	[0.029]	[0.022]	[0.037]	[0.027]	[0.071]	[0.036]	[0.04]	[0.04]
Overall maximum convergence ratio:	0.153	0.102	0.148	0.115	0.126	0.139	0.167	0.203	0.216	0.173

Table 35: Results of the meta-analysis for negative network and DG, Model 2

	est	se	N	p	tau2	Q	Qp
outdegree (density)	-2.175	0.1567	20	0.000	0.244	19.873	0.402
reciprocity	0.584	0.0814	20	0.000	0.001	17.516	0.555
transitive triplets	-0.105	0.0277	20	0.000	0.055	23.515	0.215
indegree - popularity	0.118	0.0140	20	0.000	0.000	18.314	0.502
outdegree - activity	0.098	0.0095	20	0.000	0.019	24.985	0.161
indegree - activity	-0.014	0.0460	5	0.761	0.000	1.151	0.886
outdegree-trunc(1)	-2.572	1.2347	4	0.037	1.780	6.238	0.101
sex alter	0.028	0.0693	20	0.686	0.100	33.681	0.020
sex ego	0.174	0.0628	20	0.006	0.002	24.712	0.170
same sex	-0.500	0.0593	20	0.000	0.044	28.332	0.077
grade alter	0.016	0.0344	20	0.636	0.029	18.877	0.465
grade ego	0.003	0.0385	20	0.944	0.065	19.327	0.436
grade similarity	0.019	0.1367	20	0.888	0.342	31.224	0.038
DG alter	-0.007	0.0252	20	0.768	0.000	13.061	0.835
DG ego	-0.002	0.0188	20	0.922	0.028	20.969	0.339
DG similarity	-0.204	0.0862	20	0.018	0.001	28.784	0.069
popularity alter	-0.143	0.0238	20	0.000	0.035	25.835	0.135
popularity ego	-0.004	0.0157	20	0.794	0.000	11.074	0.921
popularity ego x popularity alter	-0.017	0.0048	20	0.000	0.001	13.823	0.794
int. DG ego x DG similarity	0.018	0.0833	20	0.828	0.173	27.510	0.093

Table 36: Goodness of fit statistics, Negative & DG, Model 2

	Indegree distribution	Outdegree distribution	Geodesic Distance	Triad Census
1	0.756	0.352	0.925	0.748
2	0.825	0.949	0.985	0.972
3	0.632	0.950	0.990	0.717
4	0.300	0.333	0.277	0.122
5	0.367	0.288	0.105	0.982
6	0.970	0.054	0.024	0.997
7	0.048	0.814	0.484	0.814
8	0.913	0.564	0.078	0.970
9	0.877	0.514	0.086	0.868
10	0.909	0.007	0.772	0.843
11	0.559	0.807	0.829	0.789
12	0.239	0.487	0.892	0.991
13	0.997	0.226	0.058	0.656
14	0.413	0.196	0.425	0.942
15	0.978	0.096	0.284	0.228
16	0.781	0.741	0.076	0.745
17	0.986	0.890	0.938	1.000
18	0.032	0.383	0.383	0.469
19	0.982	0.781	0.286	0.630
20	0.540	0.568	0.942	0.683

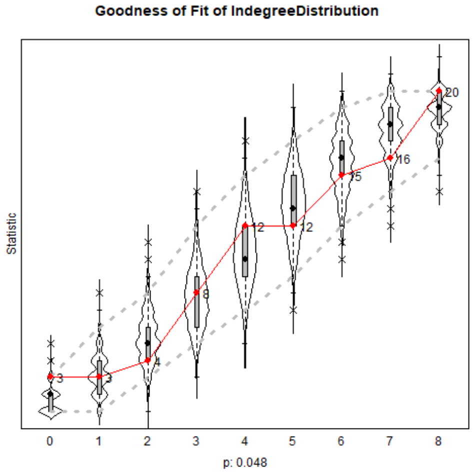

(a) Class 7

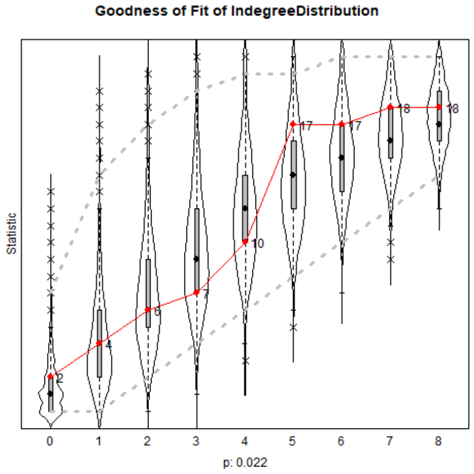

(b) Class 18

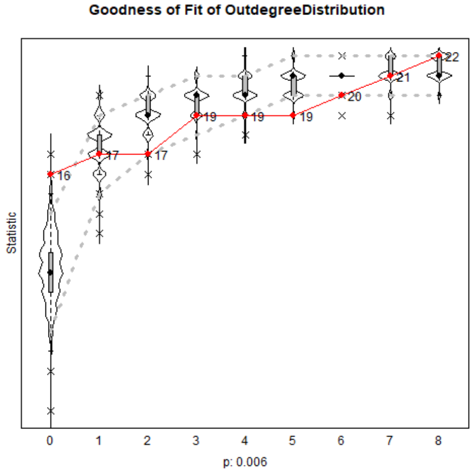

(c) Class 10

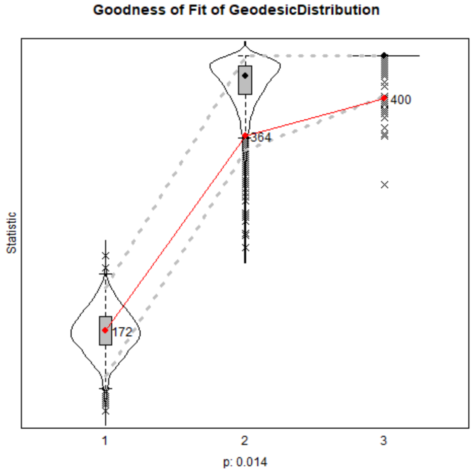

(d) Class 6

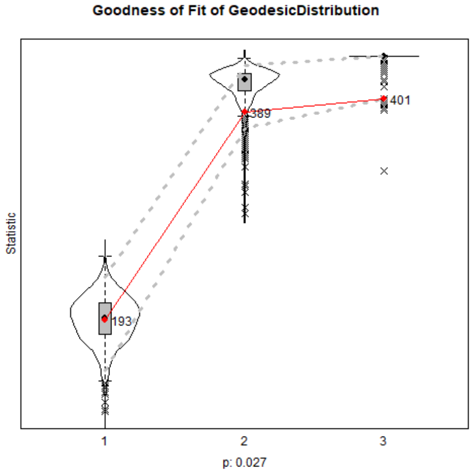

(e) Class 13

Figure 12: Classes with poor fit, Negative & DG, Model 2

Table 37: Results of separate SAOMs for negative network and DG, Model 2

	1	2	3	4	5	6	7	8	9	10
outdegree (density)	-2.082	6.667	-0.615	-2.738	-2.663	-1.021	-2.691	-1.622	-1.537	-1.784
	[0.366]	[8.002]	[1.549]	[0.838]	[0.656]	[1.264]	[0.788]	[0.387]	[0.412]	[3.34]
reciprocity	0.69	2.675	0.12	0.532	1.096	0.638	1.254	0.576	0.332	-0.04
	[0.229]	[2.065]	[0.467]	[0.517]	[0.311]	[0.445]	[0.495]	[0.21]	[0.273]	[1.213]
transitive triplets	-0.064	1.753	-0.205	0.35	-0.218	0.013	-0.169	-0.232	0.036	0.115
	[0.055]	[1.943]	[0.142]	[0.213]	[0.085]	[0.247]	[0.14]	[0.065]	[0.066]	[0.708]
indegree - popularity	0.105	-1.401	-0.302	0.064	0.086	0.13	0.132	0.078	0.026	-1.189
	[0.039]	[1.162]	[0.248]	[0.12]	[0.069]	[0.047]	[0.09]	[0.047]	[0.054]	[1.091]
outdegree - activity	0.077	-2.366	0.171	0.063	0.156	0.037	0.165	0.096	0.036	0.265
	[0.019]	[2.748]	[0.049]	[0.069]	[0.03]	[0.081]	[0.069]	[0.021]	[0.025]	[0.157]
indegree - activity	0	0	0	0	0	-0.202	0	0	0	-0.139
	[NA]	[NA]	[NA]	[NA]	[NA]	[0.232]	[NA]	[NA]	[NA]	[2.024]
outdegree-trunc(1)	0	0	0	1.299	0	0	0	-2.374	0	0
	[NA]	[NA]	[NA]	[1.957]	[NA]	[NA]	[NA]	[1.292]	[NA]	[NA]
sex alter	-0.024	-5.768	-1.908	0.003	-0.384	-0.078	-0.03	0.232	0.233	-0.502
	[0.156]	[3.966]	[0.764]	[0.42]	[0.421]	[0.283]	[0.283]	[0.191]	[0.219]	[1.003]
sex ego	-0.014	-1.786	-0.222	0.898	-0.463	-0.482	-0.15	0.116	0.372	-0.408
	[0.153]	[2.605]	[0.384]	[0.457]	[0.416]	[0.574]	[0.321]	[0.157]	[0.229]	[0.818]
same sex	-0.55	-2.691	-0.447	0.088	-1.404	-0.172	-0.522	-0.619	-0.506	-1.554
	[0.154]	[2.186]	[0.483]	[0.379]	[0.447]	[0.206]	[0.27]	[0.166]	[0.203]	[1.106]
grade alter	0.122	-0.715	-0.003	0.14	0.156	0.059	0.404	-0.265	-0.267	0.057
	[0.136]	[0.753]	[0.222]	[0.209]	[0.168]	[0.095]	[0.27]	[0.119]	[0.149]	[0.58]
grade ego	0.166	1.633	-0.074	-0.227	0.339	0.091	0.849	-0.113	-0.196	-0.601
	[0.147]	[1.84]	[0.174]	[0.209]	[0.181]	[0.11]	[0.854]	[0.102]	[0.147]	[1.291]
grade similarity	-0.036	-0.362	0.627	-0.292	0.345	-0.193	-0.193	-0.687	0.163	3.607
	[0.257]	[1.802]	[0.502]	[0.741]	[0.927]	[0.414]	[0.391]	[0.298]	[0.295]	[1.995]
DG alter	-0.018	-0.071	0.046	0.108	-0.502	0.05	0.116	0.008	0.042	0.518
	[0.068]	[0.639]	[0.143]	[0.185]	[0.241]	[0.092]	[0.219]	[0.077]	[0.075]	[0.453]
DG ego	-0.056	-2.033	-0.201	0.053	0.023	0.046	0.388	0.045	-0.092	0.02
	[0.048]	[2.014]	[0.122]	[0.134]	[0.161]	[0.079]	[0.436]	[0.044]	[0.064]	[0.306]
DG similarity	-0.083	-3.992	-0.362	0.066	1.159	-0.407	1.678	-0.084	-0.022	2.381
	[0.263]	[4.288]	[0.517]	[0.71]	[0.814]	[0.295]	[1.261]	[0.265]	[0.329]	[1.275]
popularity alter	-0.1	-0.664	-0.806	-0.257	-0.269	-0.051	-0.27	-0.192	-0.155	-1.501
	[0.058]	[0.741]	[0.322]	[0.152]	[0.097]	[0.089]	[0.134]	[0.065]	[0.089]	[0.782]
popularity ego	0.026	-2.601	-0.1	0.081	-0.055	-0.295	-0.187	-0.02	-0.082	0.108
	[0.031]	[3.686]	[0.094]	[0.117]	[0.072]	[0.317]	[0.312]	[0.04]	[0.052]	[1.463]
popularity ego x popularity alter	-0.025	0.479	0.005	-0.108	0.011	-0.015	-0.03	-0.014	0.004	0.064
	[0.008]	[0.475]	[0.032]	[0.05]	[0.036]	[0.02]	[0.049]	[0.015]	[0.017]	[0.103]
int. DG ego x DG similarity	0.188	-3.442	0.239	-0.365	1.094	-0.178	-0.887	-0.41	-0.102	-1.403
	[0.206]	[2.454]	[0.326]	[0.559]	[0.621]	[0.262]	[0.73]	[0.206]	[0.223]	[1.117]
Overall maximum convergence ratio:	0.118	0.184	0.158	0.134	0.155	0.105	0.162	0.161	0.151	0.179

	11	12	13	14	15	16	17	18	19	20
outdegree (density)	-1.885	-3.072	-1.606	-2.736	-3.263	-2.498	-2.51	-2.31	-0.482	-1.817
	[0.586]	[0.587]	[0.915]	[0.467]	[0.604]	[0.957]	[0.752]	[0.458]	[1.044]	[0.878]
reciprocity	0.488	1.009	0.81	0.634	-0.065	0.395	0.072	0.697	-1.178	-0.07
	[0.324]	[0.35]	[0.346]	[0.286]	[0.386]	[0.324]	[0.862]	[0.376]	[1.093]	[0.557]
transitive triplets	-0.234	-0.039	-0.006	-0.116	-0.259	-0.111	-0.68	-0.04	-0.654	-0.306
	[0.107]	[0.071]	[0.12]	[0.06]	[0.198]	[0.075]	[0.822]	[0.096]	[0.563]	[0.231]
indegree - popularity	0.154	0.112	0.094	0.166	0.201	0.116	-0.066	0.18	-0.053	0.019
	[0.065]	[0.064]	[0.06]	[0.04]	[0.06]	[0.045]	[0.173]	[0.044]	[0.196]	[0.195]
outdegree - activity	0.095	0.103	0.07	0.095	0.179	0.109	0.035	0.08	0.201	0.223
	[0.04]	[0.027]	[0.037]	[0.025]	[0.052]	[0.026]	[0.062]	[0.034]	[0.118]	[0.071]
indegree - activity	0	0	-0.085	0	0	-0.007	0	0.01	0	0
	[NA]	[NA]	[0.126]	[NA]	[NA]	[0.106]	[NA]	[0.057]	[NA]	[NA]
outdegree-trunc(1)	-4.57	0	0	0	0	0	0	0	-4.436	0
	[1.652]	[NA]	[NA]	[NA]	[NA]	[NA]	[NA]	[NA]	[2.032]	[NA]
sex alter	-0.131	-0.36	-0.14	0.238	1.226	0.066	-1.337	0.412	-2.177	-0.512
	[0.223]	[0.25]	[0.308]	[0.2]	[0.427]	[0.212]	[0.738]	[0.238]	[1.464]	[0.526]
sex ego	0.045	0.306	0.237	0.227	0.781	0.366	1.944	0.52	-1.017	0.052
	[0.231]	[0.27]	[0.26]	[0.194]	[0.326]	[0.288]	[0.813]	[0.252]	[0.916]	[0.427]
same sex	-0.259	-0.208	-0.45	-0.404	-1.029	-0.499	0.457	-0.997	-1.359	-1.577
	[0.207]	[0.226]	[0.263]	[0.199]	[0.324]	[0.19]	[0.597]	[0.282]	[0.725]	[0.561]
grade alter	0.04	0.074	0.139	-0.026	0.038	0.039	-0.028	-0.086	0.575	0.288
	[0.118]	[0.19]	[0.119]	[0.108]	[0.121]	[0.115]	[0.23]	[0.117]	[0.501]	[0.237]
grade ego	0	-0.115	-0.141	0.015	-0.03	0.225	-0.056	-0.015	-0.023	0.19
	[0.135]	[0.187]	[0.098]	[0.123]	[0.121]	[0.109]	[0.253]	[0.156]	[0.272]	[0.236]
grade similarity	-0.508	-0.774	-0.171	0.919	1.406	0.587	1.498	0.218	-1.366	-0.141
	[0.423]	[0.404]	[0.451]	[0.475]	[0.694]	[0.368]	[1.069]	[0.409]	[0.953]	[0.752]
DG alter	-0.115	-0.109	-0.12	-0.052	0.016	0.08	-0.007	-0.03	0.203	0.065
	[0.095]	[0.1]	[0.117]	[0.094]	[0.152]	[0.086]	[0.157]	[0.098]	[0.203]	[0.219]
DG ego	-0.02	-0.077	0.132	0.103	0.256	-0.043	-0.035	-0.019	0.046	0.15
	[0.062]	[0.069]	[0.084]	[0.063]	[0.143]	[0.044]	[0.132]	[0.049]	[0.107]	[0.217]
DG similarity	-0.145	-0.8	-0.234	-0.017	-0.98	-0.345	-2.112	0.19	-0.965	-0.231
	[0.313]	[0.345]	[0.317]	[0.291]	[0.66]	[0.271]	[0.65]	[0.269]	[0.612]	[0.848]
popularity alter	-0.228	-0.086	-0.134	-0.041	-0.06	-0.241	-0.196	-0.02	-0.824	-0.374
	[0.102]	[0.059]	[0.106]	[0.062]	[0.131]	[0.086]	[0.164]	[0.076]	[0.434]	[0.184]
popularity ego	-0.066	0.037	0.049	0.037	0.024	0.03	-0.076	0.117	-0.011	-0.05
	[0.067]	[0.043]	[0.163]	[0.053]	[0.084]	[0.194]	[0.133]	[0.137]	[0.128]	[0.099]
popularity ego x popularity alter	-0.019	-0.011	-0.019	-0.026	-0.016	0.028	-0.06	-0.026	-0.025	-0.065
	[0.02]	[0.014]	[0.032]	[0.023]	[0.04]	[0.027]	[0.069]	[0.037]	[0.041]	[0.043]
int. DG ego x DG similarity	0.292	0.366	0.635	0.35	0.213	-0.146	-0.528	-0.172	-0.395	0.611
	[0.224]	[0.268]	[0.349]	[0.271]	[0.42]	[0.228]	[0.408]	[0.254]	[0.407]	[0.708]
Overall maximum convergence ratio:	0.118	0.138	0.171	0.097	0.148	0.114	0.127	0.140	0.177	0.124

Table 38: Results of the meta-analysis for negative network and TG, Model 1

	est	se	N	p	tau2	Q	Qp
outdegree (density)	-2.098	0.1547	20	0.000	0.252	20.728	0.352
reciprocity	0.588	0.0816	20	0.000	0.000	16.830	0.601
transitive triplets	-0.104	0.0266	20	0.000	0.052	22.547	0.258
indegree - popularity	0.110	0.0142	20	0.000	0.001	17.220	0.575
outdegree - activity	0.098	0.0097	20	0.000	0.021	25.470	0.146
indegree - activity	-0.008	0.0465	4	0.863	0.000	2.165	0.539
outdegree-trunc(1)	-2.808	1.1209	4	0.012	1.548	6.060	0.109
sex alter	0.020	0.0830	20	0.810	0.195	36.574	0.009
sex ego	0.156	0.0771	20	0.043	0.163	32.600	0.027
same sex	-0.507	0.0630	20	0.000	0.091	29.936	0.053
grade alter	0.013	0.0341	20	0.692	0.000	16.551	0.620
grade ego	0.000	0.0331	20	0.990	0.000	13.231	0.827
grade similarity	0.003	0.1244	20	0.980	0.263	26.510	0.117
TG alter	-0.008	0.0207	20	0.705	0.054	34.661	0.015
TG ego	-0.011	0.0240	20	0.638	0.069	35.745	0.011
TG similarity	-0.162	0.1073	20	0.131	0.002	21.701	0.299
popularity alter	-0.146	0.0219	20	0.000	0.001	24.474	0.179
popularity ego	-0.016	0.0161	20	0.321	0.000	10.177	0.948
popularity ego x popularity alter	-0.014	0.0050	20	0.004	0.000	14.168	0.774

Table 39: Goodness of fit statistics, Negative & TG, Model 1

	Indegree distribution	Outdegree distribution	Geodesic Distance	Triad Census
1	0.773	0.560	0.962	0.647
2	0.936	0.881	0.994	0.779
3	0.643	0.961	1.000	0.758
4	0.172	0.282	0.108	0.125
5	0.325	0.112	0.080	0.981
6	0.970	0.040	0.023	0.996
7	0.044	0.791	0.291	0.877
8	0.947	0.519	0.042	0.979
9	0.895	0.449	0.077	0.913
10	0.403	0.017	0.604	0.833
11	0.683	0.722	0.894	0.725
12	0.213	0.502	0.922	0.995
13	0.992	0.133	0.043	0.544
14	0.463	0.147	0.286	0.919
15	0.989	0.275	0.488	0.337
16	0.764	0.851	0.073	0.729
17	0.988	0.845	0.961	1.000
18	0.037	0.314	0.577	0.447
19	0.982	0.741	0.282	0.653
20	0.472	0.636	0.938	0.800

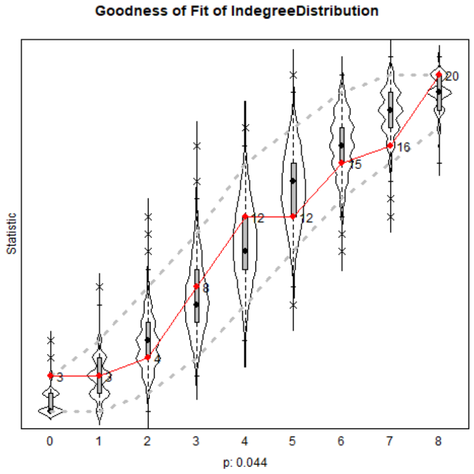

(a) Class 7

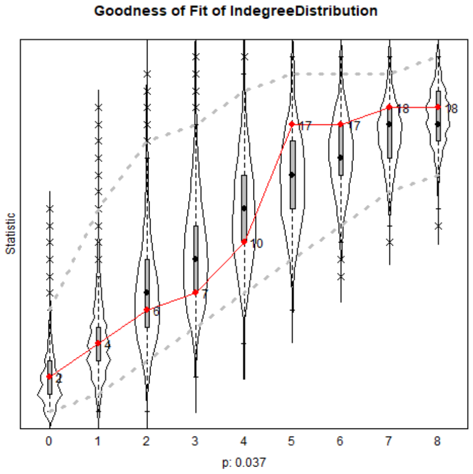

(b) Class 18

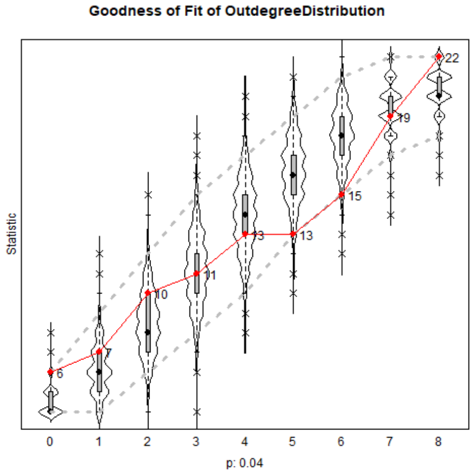

(c) Class 6

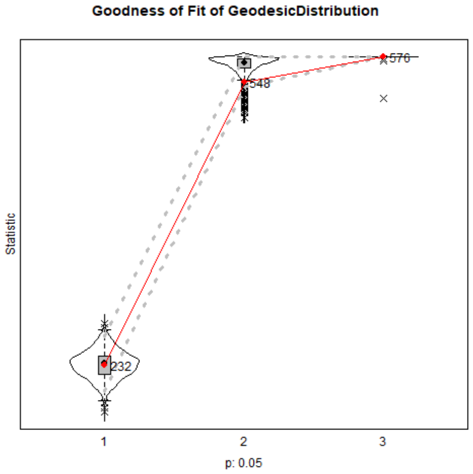

(d) Class 8

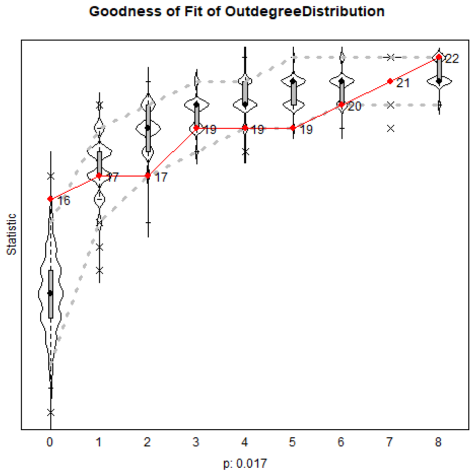

(e) Class 10

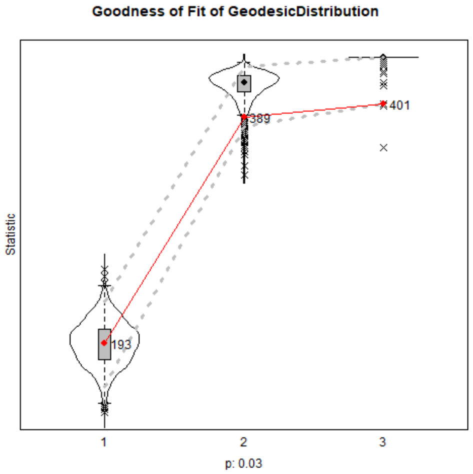

(f) Class 13

Figure 13: Classes with poor fit, Negative & TG, Model 1

Table 40: Results of separate SAOMs for negative network and TG, Model 1

	1	2	3	4	5	6	7	8	9	10
outdegree (density)	-1.894	1.612	-0.043	-2.765	-2.4	-1.139	-2.296	-1.713	-1.52	0.757
	[0.4]	[8.228]	[1.652]	[0.794]	[0.615]	[0.825]	[0.622]	[0.365]	[0.395]	[3.366]
reciprocity	0.722	2.182	0.054	0.351	1.078	0.681	1.15	0.533	0.32	0.269
	[0.229]	[1.348]	[0.491]	[0.548]	[0.315]	[0.486]	[0.367]	[0.219]	[0.272]	[1.325]
transitive triplets	-0.067	1.054	-0.163	0.354	-0.207	0.021	-0.125	-0.236	0.042	-0.045
	[0.055]	[1.142]	[0.168]	[0.241]	[0.084]	[0.165]	[0.084]	[0.069]	[0.067]	[0.658]
indegree - popularity	0.107	-0.724	-0.403	0.058	0.103	0.097	0.085	0.104	0.027	-3.027
	[0.042]	[0.638]	[0.288]	[0.117]	[0.07]	[0.047]	[0.072]	[0.042]	[0.05]	[1.984]
outdegree - activity	0.065	-1.023	0.167	0.064	0.155	0.046	0.13	0.089	0.033	0.312
	[0.02]	[2.418]	[0.061]	[0.078]	[0.031]	[0.049]	[0.031]	[0.022]	[0.025]	[0.16]
indegree - activity	0	0	0	0	0	-0.172	0	0	0	0
	[NA]	[NA]	[NA]	[NA]	[NA]	[0.146]	[NA]	[NA]	[NA]	[NA]
outdegree-trunc(1)	0	0	0	1.495	0	0	0	-2.331	0	0
	[NA]	[NA]	[NA]	[2.244]	[NA]	[NA]	[NA]	[1.385]	[NA]	[NA]
sex alter	0.053	-2.211	-2.312	0.265	-0.52	0.062	-0.034	0.274	0.234	-0.306
	[0.17]	[1.737]	[0.882]	[0.484]	[0.426]	[0.244]	[0.237]	[0.204]	[0.224]	[1.238]
sex ego	-0.107	1.297	-0.358	1.286	-0.564	-0.23	-0.162	0.251	0.349	-0.651
	[0.17]	[3.415]	[0.354]	[0.622]	[0.408]	[0.299]	[0.251]	[0.177]	[0.234]	[0.929]
same sex	-0.516	-1.35	-0.488	-0.031	-1.493	-0.125	-0.445	-0.634	-0.501	-1.49
	[0.158]	[1.044]	[0.485]	[0.405]	[0.45]	[0.193]	[0.212]	[0.168]	[0.204]	[1.001]
grade alter	0.133	-0.612	0.083	0.149	0.112	0.064	0.156	-0.237	-0.262	0.512
	[0.155]	[0.73]	[0.243]	[0.216]	[0.165]	[0.105]	[0.231]	[0.115]	[0.148]	[0.747]
grade ego	0.083	-0.104	-0.048	-0.135	0.088	0.08	0.54	-0.07	-0.184	-0.365
	[0.151]	[1.838]	[0.183]	[0.233]	[0.147]	[0.1]	[0.263]	[0.108]	[0.151]	[0.905]
grade similarity	0.004	-0.749	0.75	-0.305	-0.256	-0.324	-0.324	-0.591	0.158	4.056
	[0.275]	[2.208]	[0.497]	[0.808]	[0.863]	[0.453]	[0.345]	[0.306]	[0.29]	[2]
TG alter	0.027	-1.051	0.198	0.13	-0.027	-0.151	-0.227	-0.093	-0.041	1.735
	[0.037]	[0.837]	[0.161]	[0.08]	[0.048]	[0.076]	[0.141]	[0.06]	[0.067]	[0.982]
TG ego	-0.117	-0.732	-0.211	0.186	-0.128	-0.061	0.055	0.115	-0.019	-0.381
	[0.048]	[1.043]	[0.104]	[0.113]	[0.052]	[0.089]	[0.145]	[0.056]	[0.074]	[0.359]
TG similarity	0.062	-7.4	-0.489	0.762	-0.776	-0.002	1.108	-0.344	-0.218	3.587
	[0.405]	[6.297]	[0.742]	[0.864]	[0.462]	[0.411]	[0.533]	[0.34]	[0.384]	[2.029]
popularity alter	-0.087	-0.361	-0.991	-0.32	-0.244	-0.08	-0.335	-0.174	-0.124	-2.515
	[0.061]	[0.601]	[0.447]	[0.166]	[0.102]	[0.077]	[0.151]	[0.061]	[0.084]	[1.262]
popularity ego	0.014	-2.086	-0.099	-0.002	-0.055	-0.222	-0.001	-0.031	-0.096	-0.04
	[0.035]	[5.215]	[0.103]	[0.129]	[0.068]	[0.183]	[0.09]	[0.04]	[0.058]	[0.496]
popularity ego x popularity alter	-0.021	0.16	0.029	-0.119	0.02	-0.017	0.014	-0.019	0.007	0.073
	[0.009]	[0.355]	[0.036]	[0.053]	[0.035]	[0.02]	[0.038]	[0.015]	[0.017]	[0.123]
Overall maximum convergence ratio:	0.138	0.168	0.139	0.152	0.137	0.165	0.172	0.144	0.128	0.208

	11	12	13	14	15	16	17	18	19	20
outdegree (density)	-1.746	-2.872	-1.576	-2.583	-3.112	-2.756	-2.834	-2.421	-0.37	-1.585
	[0.618]	[0.538]	[0.942]	[0.492]	[0.512]	[1.211]	[0.928]	[0.455]	[0.994]	[0.921]
reciprocity	0.466	0.959	0.809	0.578	-0.089	0.451	0.256	0.528	-0.96	0.152
	[0.368]	[0.328]	[0.341]	[0.277]	[0.392]	[0.306]	[0.797]	[0.42]	[1.062]	[0.598]
transitive triplets	-0.251	-0.042	-0.004	-0.113	-0.187	-0.124	-0.91	-0.08	-0.548	-0.288
	[0.093]	[0.061]	[0.122]	[0.062]	[0.144]	[0.105]	[0.932]	[0.097]	[0.541]	[0.232]
indegree - popularity	0.111	0.099	0.097	0.154	0.186	0.11	0.005	0.174	-0.05	-0.018
	[0.066]	[0.063]	[0.061]	[0.043]	[0.054]	[0.062]	[0.181]	[0.044]	[0.186]	[0.207]
outdegree - activity	0.107	0.099	0.065	0.095	0.165	0.098	0.052	0.098	0.164	0.211
	[0.036]	[0.024]	[0.041]	[0.027]	[0.04]	[0.031]	[0.066]	[0.031]	[0.123]	[0.066]
indegree - activity	0	0	-0.073	0	0	0.061	0	0.023	0	0
	[NA]	[NA]	[0.113]	[NA]	[NA]	[0.128]	[NA]	[0.06]	[NA]	[NA]
outdegree-trunc(1)	-4.478	0	0	0	0	0	0	0	-4.335	0
	[1.38]	[NA]	[NA]	[NA]	[NA]	[NA]	[NA]	[NA]	[1.635]	[NA]
sex alter	-0.335	-0.257	-0.125	0.29	1.19	0.067	-1.121	0.17	-1.813	-0.785
	[0.224]	[0.252]	[0.308]	[0.197]	[0.356]	[0.231]	[0.708]	[0.234]	[1.506]	[0.518]
sex ego	0.01	0.19	0.258	0.301	0.802	0.512	2.452	0.486	-1.02	-0.032
	[0.228]	[0.222]	[0.277]	[0.199]	[0.306]	[0.352]	[0.954]	[0.25]	[0.796]	[0.377]
same sex	-0.247	-0.272	-0.428	-0.482	-1.063	-0.507	0.575	-1.03	-1.498	-1.481
	[0.222]	[0.212]	[0.285]	[0.201]	[0.327]	[0.191]	[0.585]	[0.301]	[0.706]	[0.514]
grade alter	0.059	-0.011	0.126	-0.038	0.041	0.03	0.117	-0.095	0.572	0.269
	[0.123]	[0.198]	[0.115]	[0.108]	[0.123]	[0.118]	[0.194]	[0.121]	[0.465]	[0.236]
grade ego	-0.003	-0.122	-0.135	0.013	-0.07	0.186	0.039	0.011	0.049	0.014
	[0.131]	[0.19]	[0.1]	[0.125]	[0.117]	[0.117]	[0.197]	[0.123]	[0.247]	[0.195]
grade similarity	-0.399	-0.563	-0.2	0.794	1.161	0.703	0.85	-0.136	-1.025	0.053
	[0.43]	[0.418]	[0.432]	[0.463]	[0.62]	[0.393]	[1.027]	[0.42]	[1.005]	[0.703]
TG alter	0.077	-0.04	0.044	0.095	0.069	-0.042	-0.133	-0.116	-0.088	0.163
	[0.048]	[0.05]	[0.063]	[0.063]	[0.058]	[0.067]	[0.091]	[0.059]	[0.136]	[0.133]
TG ego	0.032	-0.036	0.067	0.142	-0.106	0.113	0.075	-0.014	-0.041	-0.086
	[0.046]	[0.053]	[0.069]	[0.07]	[0.06]	[0.105]	[0.111]	[0.056]	[0.084]	[0.113]
TG similarity	0.146	-0.341	-0.575	-0.134	-0.283	-0.026	-1.952	-0.053	-0.408	0.187
	[0.502]	[0.36]	[0.406]	[0.352]	[0.707]	[0.3]	[0.795]	[0.592]	[0.717]	[0.646]
popularity alter	-0.299	-0.121	-0.13	-0.054	-0.042	-0.255	-0.267	-0.08	-0.778	-0.415
	[0.115]	[0.06]	[0.106]	[0.067]	[0.109]	[0.102]	[0.155]	[0.087]	[0.39]	[0.195]
popularity ego	-0.049	0.016	0.034	0.044	-0.026	0.193	-0.114	0.126	-0.015	-0.061
	[0.067]	[0.042]	[0.157]	[0.056]	[0.084]	[0.27]	[0.108]	[0.129]	[0.124]	[0.098]
popularity ego x popularity alter	-0.022	-0.011	-0.016	-0.03	-0.024	0.027	-0.041	-0.019	-0.024	-0.051
	[0.02]	[0.013]	[0.03]	[0.023]	[0.034]	[0.026]	[0.07]	[0.039]	[0.043]	[0.041]
Overall maximum convergence ratio:	0.112	0.095	0.164	0.118	0.140	0.147	0.149	0.189	0.190	0.144

Table 41: Results of the meta-analysis for negative network and TG, Model 2

	est	se	N	p	tau2	Q	Qp
outdegree (density)	-2.131	0.1539	20	0.000	0.218	19.339	0.435
reciprocity	0.582	0.0810	20	0.000	0.000	16.496	0.624
transitive triplets	-0.107	0.0271	20	0.000	0.054	23.497	0.216
indegree - popularity	0.112	0.0145	20	0.000	0.001	15.985	0.658
outdegree - activity	0.098	0.0102	20	0.000	0.024	27.767	0.088
indegree - activity	-0.004	0.0480	4	0.926	0.000	2.206	0.531
outdegree-trunc(1)	-2.834	1.0569	4	0.007	1.343	5.641	0.130
sex alter	0.027	0.0818	20	0.745	0.182	35.391	0.013
sex ego	0.165	0.0762	20	0.030	0.147	31.195	0.038
same sex	-0.507	0.0641	20	0.000	0.097	29.730	0.055
grade alter	0.011	0.0340	20	0.745	0.000	16.505	0.623
grade ego	0.001	0.0334	20	0.965	0.000	11.489	0.906
grade similarity	0.004	0.1253	20	0.974	0.264	25.781	0.136
TG alter	0.015	0.0205	20	0.479	0.012	19.952	0.397
TG ego	-0.021	0.0252	20	0.403	0.068	32.162	0.030
TG similarity	-0.126	0.1151	20	0.272	0.001	19.912	0.400
popularity alter	-0.144	0.0221	20	0.000	0.001	23.530	0.215
popularity ego	-0.017	0.0167	20	0.316	0.000	9.772	0.958
popularity ego x popularity alter	-0.015	0.0049	20	0.002	0.000	14.188	0.773
int. TG ego x TG similarity	-0.087	0.0596	20	0.144	0.000	20.224	0.381

Table 42: Goodness of fit statistics, Negative & TG, Model 2

	1	2	3	4
1	0.790	0.579	0.962	0.656
2	0.926	0.925	0.991	0.783
3	0.725	0.956	1.000	0.719
4	0.300	0.332	0.086	0.117
5	0.321	0.129	0.054	0.977
6	0.973	0.017	0.010	0.998
7	0.027	0.812	0.335	0.840
8	0.944	0.596	0.044	0.976
9	0.864	0.498	0.088	0.915
10	0.500	0.042	0.648	0.852
11	0.688	0.736	0.870	0.756
12	0.167	0.497	0.907	0.992
13	0.991	0.172	0.029	0.517
14	0.376	0.128	0.349	0.938
15	0.988	0.265	0.525	0.310
16	0.796	0.834	0.085	0.760
17	0.996	0.822	0.959	1.000
18	0.053	0.307	0.537	0.414
19	0.986	0.753	0.208	0.689
20	0.448	0.602	0.937	0.841

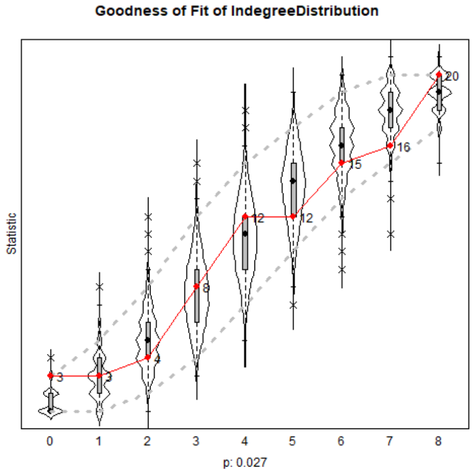

(a) Class 7

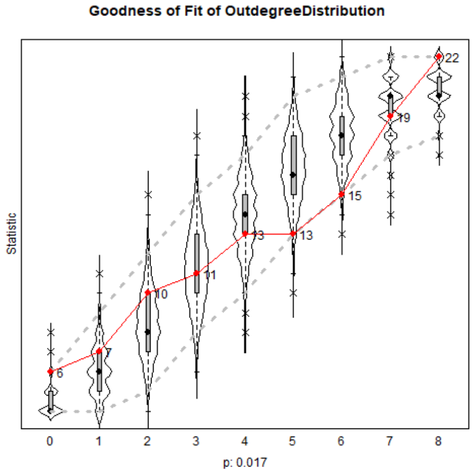

(b) Class 6

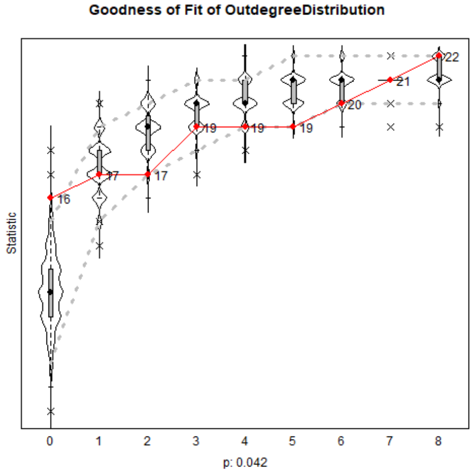

(c) Class 10

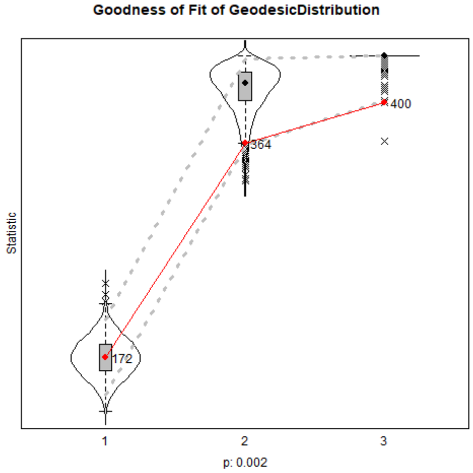

(d) Class 6

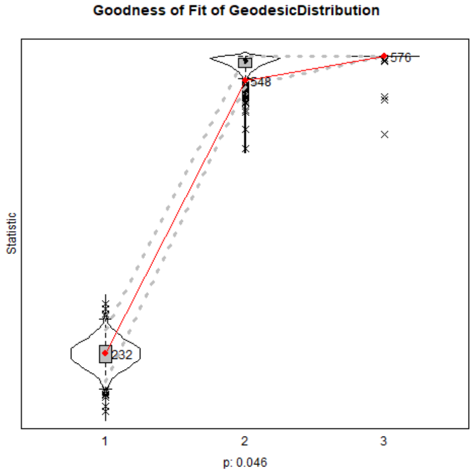

(e) Class 8

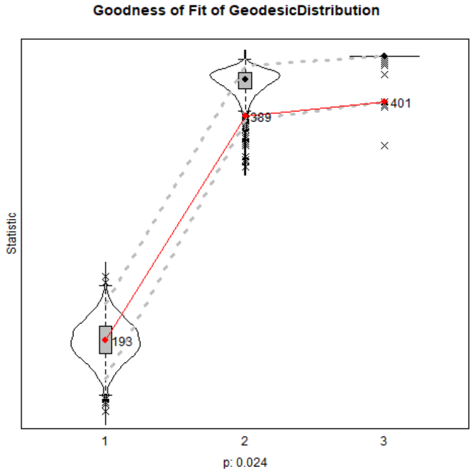

(f) Class 13

Figure 14: Classes with poor fit, Negative & TG, Model 2

Table 43: Results of separate SAOMs for negative network and TG, Model 2

	1	2	3	4	5	6	7	8	9	10
outdegree (density)	-1.901	3.088	-0.073	-2.719	-2.435	-1.304	-2.499	-1.688	-1.526	0.473
	[0.398]	[6.844]	[1.595]	[0.837]	[0.616]	[0.852]	[0.735]	[0.376]	[0.403]	[3.504]
reciprocity	0.715	2.756	0.12	0.327	1.07	0.672	1.201	0.528	0.314	0.285
	[0.229]	[1.974]	[0.47]	[0.527]	[0.302]	[0.465]	[0.416]	[0.22]	[0.276]	[1.329]
transitive triplets	-0.065	1.31	-0.185	0.358	-0.214	0.006	-0.14	-0.239	0.036	-0.026
	[0.055]	[1.282]	[0.166]	[0.239]	[0.084]	[0.142]	[0.091]	[0.071]	[0.062]	[0.76]
indegree - popularity	0.106	-0.979	-0.402	0.058	0.102	0.097	0.1	0.105	0.029	-3.062
	[0.04]	[0.867]	[0.279]	[0.114]	[0.07]	[0.049]	[0.077]	[0.043]	[0.053]	[2.069]
outdegree - activity	0.064	-1.8	0.17	0.058	0.157	0.052	0.145	0.087	0.034	0.329
	[0.021]	[2.556]	[0.055]	[0.075]	[0.03]	[0.045]	[0.039]	[0.024]	[0.024]	[0.188]
indegree - activity	0	0	0	0	0	-0.15	0	0	0	0
	[NA]	[NA]	[NA]	[NA]	[NA]	[0.134]	[NA]	[NA]	[NA]	[NA]
outdegree-trunc(1)	0	0	0	1.535	0	0	0	-2.433	0	0
	[NA]	[NA]	[NA]	[2.305]	[NA]	[NA]	[NA]	[1.305]	[NA]	[NA]
sex alter	0.058	-2.789	-2.227	0.301	-0.534	0.054	-0.067	0.281	0.241	-0.379
	[0.162]	[2.079]	[0.884]	[0.524]	[0.428]	[0.236]	[0.265]	[0.203]	[0.226]	[1.139]
sex ego	-0.113	1.855	-0.291	1.42	-0.578	-0.193	-0.187	0.262	0.331	-0.783
	[0.171]	[3.968]	[0.349]	[0.645]	[0.414]	[0.292]	[0.27]	[0.18]	[0.245]	[0.944]
same sex	-0.524	-1.256	-0.371	-0.037	-1.485	-0.134	-0.482	-0.63	-0.503	-1.362
	[0.159]	[1.086]	[0.512]	[0.433]	[0.45]	[0.191]	[0.235]	[0.169]	[0.206]	[1.067]
grade alter	0.136	-0.676	0.091	0.141	0.109	0.065	0.172	-0.236	-0.257	0.436
	[0.145]	[0.787]	[0.283]	[0.222]	[0.168]	[0.098]	[0.211]	[0.117]	[0.141]	[0.964]
grade ego	0.081	0.29	-0.113	-0.152	0.084	0.079	0.527	-0.072	-0.161	-0.416
	[0.155]	[1.399]	[0.205]	[0.236]	[0.144]	[0.1]	[0.331]	[0.106]	[0.154]	[1.092]
grade similarity	0	-0.341	0.769	-0.324	-0.213	-0.34	-0.357	-0.607	0.14	4.014
	[0.267]	[1.66]	[0.538]	[0.828]	[0.901]	[0.423]	[0.379]	[0.311]	[0.295]	[2.105]
TG alter	0.04	-0.812	0.02	0.091	0.002	-0.079	-0.02	-0.074	-0.008	1.976
	[0.049]	[0.834]	[0.185]	[0.089]	[0.07]	[0.105]	[0.161]	[0.075]	[0.085]	[1.129]
TG ego	-0.13	-1.813	-0.184	0.225	-0.141	-0.082	-0.153	0.111	-0.037	-0.517
	[0.059]	[2.049]	[0.107]	[0.132]	[0.057]	[0.091]	[0.17]	[0.058]	[0.076]	[0.437]
TG similarity	0.153	-11.072	-0.682	0.592	-0.657	0.172	1.126	-0.303	-0.196	3.651
	[0.445]	[10.907]	[0.747]	[0.874]	[0.479]	[0.46]	[0.588]	[0.357]	[0.384]	[1.971]
popularity alter	-0.089	-0.564	-0.99	-0.315	-0.246	-0.079	-0.338	-0.173	-0.123	-2.502
	[0.059]	[0.704]	[0.416]	[0.168]	[0.097]	[0.071]	[0.163]	[0.062]	[0.087]	[1.446]
popularity ego	0.013	-3.085	-0.078	-0.005	-0.052	-0.195	0.01	-0.033	-0.096	-0.024
	[0.038]	[5.149]	[0.112]	[0.136]	[0.069]	[0.181]	[0.12]	[0.042]	[0.058]	[0.579]
popularity ego x popularity alter	-0.021	0.194	0.02	-0.119	0.019	-0.017	0.024	-0.018	0.006	0.072
	[0.009]	[0.339]	[0.037]	[0.052]	[0.035]	[0.017]	[0.039]	[0.015]	[0.017]	[0.149]
int. TG ego x TG similarity	-0.059	-3.261	0.882	0.263	-0.11	-0.327	-1.456	-0.088	-0.162	-1.129
	[0.145]	[4.27]	[0.614]	[0.348]	[0.198]	[0.313]	[0.624]	[0.226]	[0.221]	[1.588]
Overall maximum convergence ratio:	0.168	0.188	0.157	0.134	0.108	0.120	0.245	0.195	0.106	0.153

	11	12	13	14	15	16	17	18	19	20
outdegree (density)	-1.855	-2.932	-1.435	-2.584	-3.115	-2.824	-2.684	-2.536	-0.363	-1.84
	[0.628]	[0.611]	[1.14]	[0.477]	[0.556]	[1.196]	[0.818]	[0.431]	[1.111]	[0.893]
reciprocity	0.5	0.986	0.843	0.58	-0.084	0.422	0.253	0.481	-0.946	0.15
	[0.339]	[0.327]	[0.341]	[0.276]	[0.369]	[0.287]	[0.87]	[0.432]	[1.297]	[0.613]
transitive triplets	-0.25	-0.05	0.008	-0.113	-0.189	-0.124	-0.92	-0.085	-0.563	-0.31
	[0.092]	[0.07]	[0.129]	[0.059]	[0.157]	[0.097]	[0.922]	[0.099]	[0.537]	[0.22]
indegree - popularity	0.111	0.104	0.092	0.153	0.187	0.114	0.013	0.174	-0.051	0.031
	[0.067]	[0.07]	[0.065]	[0.043]	[0.061]	[0.062]	[0.159]	[0.044]	[0.19]	[0.204]
outdegree - activity	0.11	0.102	0.059	0.095	0.166	0.098	0.048	0.098	0.168	0.22
	[0.036]	[0.027]	[0.044]	[0.025]	[0.044]	[0.028]	[0.064]	[0.03]	[0.11]	[0.065]
indegree - activity	0	0	-0.092	0	0	0.07	0	0.028	0	0
	[NA]	[NA]	[0.134]	[NA]	[NA]	[0.131]	[NA]	[0.062]	[NA]	[NA]
outdegree-trunc(1)	-4.415	0	0	0	0	0	0	0	-4.348	0
	[1.454]	[NA]	[NA]	[NA]	[NA]	[NA]	[NA]	[NA]	[1.691]	[NA]
sex alter	-0.338	-0.274	-0.154	0.29	1.19	0.063	-1.064	0.198	-1.85	-0.662
	[0.255]	[0.245]	[0.337]	[0.195]	[0.363]	[0.225]	[0.668]	[0.248]	[1.435]	[0.535]
sex ego	0.077	0.164	0.247	0.3	0.789	0.531	2.259	0.484	-1.007	0.17
	[0.249]	[0.24]	[0.271]	[0.195]	[0.311]	[0.341]	[0.939]	[0.263]	[0.757]	[0.427]
same sex	-0.266	-0.281	-0.422	-0.474	-1.072	-0.517	0.519	-1.048	-1.502	-1.533
	[0.219]	[0.204]	[0.303]	[0.207]	[0.328]	[0.184]	[0.507]	[0.307]	[0.7]	[0.546]
grade alter	0.056	-0.017	0.131	-0.04	0.039	0.035	0.103	-0.101	0.579	0.279
	[0.122]	[0.191]	[0.127]	[0.106]	[0.13]	[0.118]	[0.196]	[0.119]	[0.506]	[0.232]
grade ego	0.033	-0.14	-0.118	0.014	-0.074	0.192	0.073	-0.019	0.059	0.063
	[0.132]	[0.191]	[0.105]	[0.113]	[0.115]	[0.118]	[0.204]	[0.133]	[0.244]	[0.203]
grade similarity	-0.393	-0.564	-0.055	0.793	1.158	0.734	0.887	-0.123	-1.015	0.044
	[0.413]	[0.419]	[0.455]	[0.457]	[0.649]	[0.385]	[1.082]	[0.41]	[1.019]	[0.737]
TG alter	0.114	-0.109	0.15	0.092	0.058	-0.078	-0.181	-0.05	-0.056	0
	[0.066]	[0.107]	[0.079]	[0.095]	[0.068]	[0.101]	[0.107]	[0.076]	[0.191]	[0.156]
TG ego	0	-0.037	0.019	0.142	-0.096	0.125	0.102	-0.055	-0.049	0.024
	[0.058]	[0.054]	[0.089]	[0.07]	[0.071]	[0.107]	[0.127]	[0.07]	[0.091]	[0.142]
TG similarity	0.391	-0.4	-0.379	-0.121	-0.302	-0.098	-1.961	0.363	-0.391	0.109
	[0.575]	[0.387]	[0.439]	[0.355]	[0.768]	[0.331]	[0.842]	[0.693]	[0.824]	[0.736]
popularity alter	-0.296	-0.12	-0.137	-0.053	-0.041	-0.247	-0.258	-0.078	-0.785	-0.378
	[0.123]	[0.065]	[0.116]	[0.069]	[0.11]	[0.102]	[0.156]	[0.086]	[0.374]	[0.194]
popularity ego	-0.064	0.017	-0.01	0.044	-0.02	0.215	-0.131	0.137	-0.023	-0.035
	[0.069]	[0.042]	[0.193]	[0.056]	[0.081]	[0.288]	[0.114]	[0.139]	[0.123]	[0.095]
popularity ego x popularity alter	-0.024	-0.01	-0.022	-0.03	-0.024	0.029	-0.042	-0.02	-0.027	-0.054
	[0.02]	[0.014]	[0.031]	[0.023]	[0.035]	[0.028]	[0.07]	[0.04]	[0.046]	[0.041]
int. TG ego x TG similarity	-0.14	0.272	-0.641	0.004	0.107	0.123	0.301	-0.231	-0.105	0.838
	[0.156]	[0.38]	[0.308]	[0.312]	[0.311]	[0.238]	[0.429]	[0.175]	[0.422]	[0.518]
Overall maximum convergence ratio:	0.130	0.140	0.130	0.082	0.094	0.164	0.156	0.212	0.174	0.148

Table 44: Results of the meta-analysis for negative network and TGB, Model 1

	est	se	N	p	tau2	Q	Qp
outdegree (density)	-2.110	0.1375	20	0.000	0.021	15.599	0.684
reciprocity	0.577	0.0799	20	0.000	0.002	19.787	0.407
transitive triplets	-0.096	0.0249	20	0.000	0.044	22.501	0.260
indegree - popularity	0.112	0.0137	20	0.000	0.001	14.329	0.764
outdegree - activity	0.098	0.0099	20	0.000	0.022	25.912	0.133
indegree - activity	-0.012	0.0421	4	0.785	0.000	1.189	0.756
outdegree-trunc(1)	-2.195	1.2878	4	0.088	1.984	7.211	0.065
sex alter	0.007	0.0782	20	0.929	0.169	36.713	0.009
sex ego	0.173	0.0737	19	0.019	0.137	28.562	0.054
same sex	-0.507	0.0607	20	0.000	0.068	26.224	0.124
grade alter	0.039	0.0351	20	0.264	0.037	17.590	0.550
grade ego	0.019	0.0355	20	0.597	0.035	17.817	0.535
grade similarity	-0.026	0.1119	20	0.819	0.168	24.967	0.162
TGB alter	-0.003	0.0157	20	0.844	0.001	11.878	0.891
TGB ego	-0.008	0.0264	20	0.773	0.071	33.082	0.024
TGB similarity	-0.205	0.2129	20	0.337	0.549	31.947	0.032
popularity alter	-0.145	0.0220	20	0.000	0.021	28.671	0.071
popularity ego	-0.019	0.0162	20	0.250	0.010	12.120	0.880
popularity ego x popularity alter	-0.015	0.0048	20	0.002	0.000	15.762	0.673

Table 45: Goodness of fit statistics, Negative & TGB, Model 1

	Indegree distribution	Outdegree distribution	Geodesic Distance	Triad Census
1	0.743	0.508	0.938	0.734
2	1.000	0.877	0.947	0.908
3	0.656	0.929	1.000	0.762
4	0.166	0.317	0.207	0.100
5	0.357	0.120	0.046	0.986
6	0.963	0.045	0.030	0.999
7	0.028	0.774	0.173	0.797
8	0.929	0.642	0.100	0.978
9	0.912	0.467	0.076	0.871
10	0.924	0.093	0.859	0.775
11	0.612	0.735	0.880	0.755
12	0.179	0.528	0.833	0.987
13	0.990	0.075	0.030	0.519
14	0.376	0.275	0.748	0.901
15	0.995	0.171	0.363	0.242
16	0.727	0.704	0.033	0.683
17	0.986	0.800	0.961	1.000
18	0.036	0.306	0.247	0.455
19	0.975	0.745	0.341	0.377
20	0.444	0.534	0.945	0.753

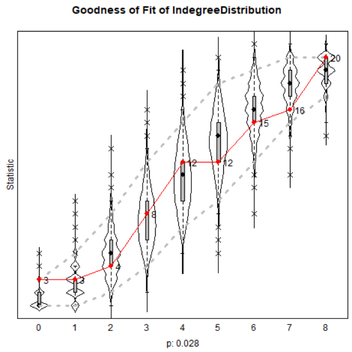

(a) Class 7

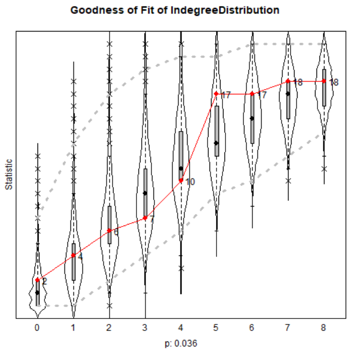

(b) Class 18

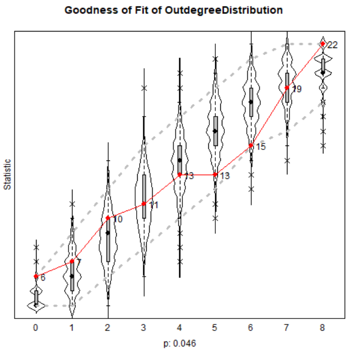

(c) Class 6

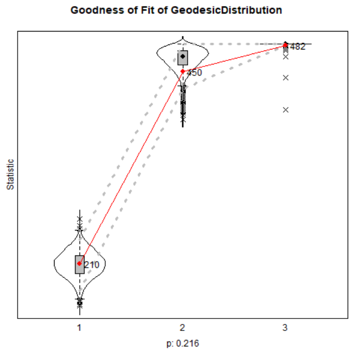

(d) Class 5

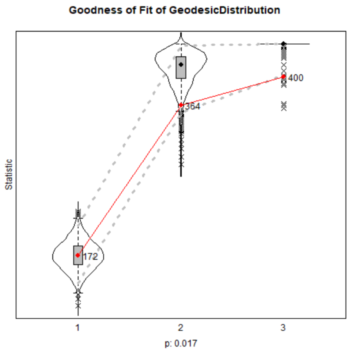

(e) Class 6

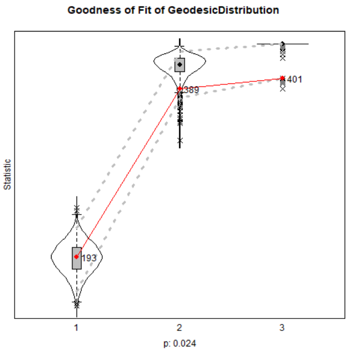

(f) Class 13

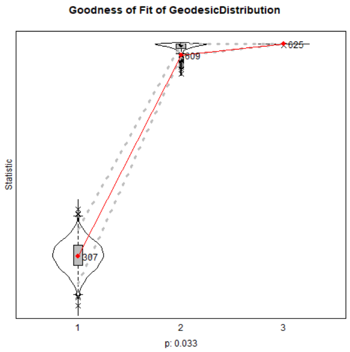

(g) Class 16

Figure 15: Classes with poor fit, Negative & TGB, Model 1

Table 46: Results of separate SAOMs for negative network and TGB, Model 1

	1	2	3	4	5	6	7	8	9	10
outdegree (density)	-1.979	1.384	-0.602	-3.14	-2.335	-1.346	-2.499	-1.905	-1.434	-2.125
	[0.346]	[6.918]	[1.439]	[1.087]	[0.605]	[1.045]	[0.684]	[0.385]	[0.405]	[3.593]
reciprocity	0.659	1.915	0.04	0.245	1.078	0.62	1.205	0.446	0.353	-0.09
	[0.223]	[1.203]	[0.497]	[0.612]	[0.303]	[0.429]	[0.406]	[0.211]	[0.261]	[1.37]
transitive triplets	-0.07	1.504	-0.2	0.312	-0.207	0.005	-0.101	-0.248	0.038	-0.049
	[0.05]	[1.545]	[0.147]	[0.246]	[0.081]	[0.175]	[0.1]	[0.071]	[0.068]	[0.629]
indegree - popularity	0.099	-0.183	-0.256	0.059	0.106	0.123	0.094	0.112	0.016	-1.319
	[0.035]	[0.46]	[0.241]	[0.131]	[0.068]	[0.043]	[0.072]	[0.043]	[0.053]	[1.279]
outdegree - activity	0.075	-1.358	0.16	0.119	0.145	0.051	0.142	0.107	0.033	0.344
	[0.017]	[2.296]	[0.048]	[0.087]	[0.029]	[0.061]	[0.045]	[0.024]	[0.025]	[0.295]
indegree - activity	0	0	0	0	0	-0.164	0	0	0	0
	[NA]	[NA]	[NA]	[NA]	[NA]	[0.17]	[NA]	[NA]	[NA]	[NA]
outdegree-trunc(1)	0	0	0	1.779	0	0	0	-2.134	0	0
	[NA]	[NA]	[NA]	[1.881]	[NA]	[NA]	[NA]	[1.297]	[NA]	[NA]
sex alter	-0.04	-3.859	-1.771	0.015	-0.442	-0.049	0.047	0.331	0.236	-0.018
	[0.153]	[3.002]	[0.661]	[0.496]	[0.402]	[0.249]	[0.232]	[0.189]	[0.217]	[2.003]
sex ego	-0.06	-0.48	-0.44	1.035	-0.376	-0.278	0.204	0.042	0.334	0
	[0.145]	[2.327]	[0.37]	[0.524]	[0.378]	[0.35]	[0.301]	[0.163]	[0.219]	[NA]
same sex	-0.515	-0.595	-0.562	-0.238	-1.388	-0.138	-0.446	-0.657	-0.528	-1.005
	[0.158]	[1.13]	[0.47]	[0.419]	[0.428]	[0.195]	[0.226]	[0.161]	[0.205]	[1.213]
grade alter	0.097	-0.224	0.031	0.622	0.15	0.023	0.339	-0.172	-0.238	0.156
	[0.14]	[0.469]	[0.245]	[0.366]	[0.158]	[0.1]	[0.171]	[0.098]	[0.15]	[0.599]
grade ego	0.106	0.654	-0.027	0.079	0.177	0.022	0.407	-0.177	-0.16	-1.627
	[0.139]	[1.083]	[0.175]	[0.256]	[0.143]	[0.114]	[0.255]	[0.104]	[0.146]	[2.292]
grade similarity	0.009	-0.546	0.721	0.251	-0.617	-0.513	-0.149	-0.639	0.103	3.563
	[0.259]	[1.781]	[0.525]	[0.863]	[0.9]	[0.46]	[0.344]	[0.316]	[0.303]	[2.093]
TGB alter	0.034	-1.767	0.02	-0.125	-0.036	0.022	-0.043	-0.023	-0.059	0.161
	[0.034]	[1.641]	[0.121]	[0.186]	[0.05]	[0.078]	[0.077]	[0.038]	[0.082]	[0.302]
TGB ego	-0.031	-2.008	-0.166	-0.154	-0.114	0.048	0.192	0.089	-0.141	0.613
	[0.035]	[2.581]	[0.094]	[0.182]	[0.057]	[0.096]	[0.135]	[0.044]	[0.087]	[0.455]
TGB similarity	0.53	-4.518	-1.082	-4.815	-0.427	1.417	0.486	0.011	-1.284	0.573
	[0.398]	[4.961]	[0.957]	[2.821]	[0.598]	[1.016]	[0.494]	[0.42]	[0.849]	[1.521]
popularity alter	-0.106	-0.016	-0.724	-0.581	-0.246	-0.057	-0.35	-0.165	-0.161	-1.605
	[0.052]	[0.42]	[0.351]	[0.233]	[0.103]	[0.07]	[0.118]	[0.061]	[0.086]	[0.979]
popularity ego	0.027	-1.544	-0.19	-0.052	-0.015	-0.202	-0.007	-0.039	-0.069	0.551
	[0.03]	[3.319]	[0.1]	[0.154]	[0.067]	[0.211]	[0.098]	[0.041]	[0.05]	[1.116]
popularity ego x popularity alter	-0.023	0.247	0.01	-0.146	0.021	-0.018	0.015	-0.02	0.008	0.077
	[0.008]	[0.319]	[0.035]	[0.066]	[0.035]	[0.018]	[0.035]	[0.016]	[0.016]	[0.14]
Overall maximum convergence ratio:	0.136	0.131	0.186	0.127	0.128	0.159	0.152	0.201	0.082	0.153

	11	12	13	14	15	16	17	18	19	20
outdegree (density)	-1.846	-2.875	-1.553	-2.482	-3.133	-2.29	-2.43	-2.315	-0.808	-1.74
	[0.65]	[0.571]	[0.907]	[0.517]	[0.536]	[1.078]	[0.871]	[0.409]	[0.913]	[0.865]
reciprocity	0.554	0.912	0.804	0.691	-0.273	0.398	0.435	0.717	-2.005	0.042
	[0.352]	[0.325]	[0.303]	[0.278]	[0.394]	[0.315]	[0.861]	[0.396]	[1.58]	[0.566]
transitive triplets	-0.222	-0.038	-0.005	-0.076	-0.217	-0.104	-0.871	-0.036	-0.802	-0.307
	[0.107]	[0.069]	[0.112]	[0.052]	[0.158]	[0.081]	[0.843]	[0.088]	[0.552]	[0.227]
indegree - popularity	0.102	0.108	0.081	0.133	0.192	0.105	-0.071	0.177	0	0.05
	[0.073]	[0.066]	[0.059]	[0.045]	[0.055]	[0.053]	[0.189]	[0.04]	[0.213]	[0.191]
outdegree - activity	0.104	0.087	0.063	0.086	0.176	0.108	0.013	0.076	0.246	0.209
	[0.041]	[0.026]	[0.036]	[0.024]	[0.045]	[0.025]	[0.093]	[0.034]	[0.113]	[0.065]
indegree - activity	0	0	-0.059	0	0	-0.025	0	0.012	0	0
	[NA]	[NA]	[0.122]	[NA]	[NA]	[0.12]	[NA]	[0.05]	[NA]	[NA]
outdegree-trunc(1)	-4.549	0	0	0	0	0	0	0	-3.479	0
	[1.553]	[NA]	[NA]	[NA]	[NA]	[NA]	[NA]	[NA]	[1.884]	[NA]
sex alter	-0.216	-0.327	-0.319	0.168	1.265	0.049	-1.205	0.27	-2.942	-0.656
	[0.238]	[0.289]	[0.287]	[0.195]	[0.404]	[0.228]	[0.847]	[0.211]	[1.606]	[0.52]
sex ego	0.103	0.514	0.078	0.343	0.931	0.463	1.928	0.364	-2.025	-0.171
	[0.247]	[0.322]	[0.265]	[0.198]	[0.348]	[0.251]	[0.996]	[0.235]	[1.294]	[0.394]
same sex	-0.235	-0.23	-0.432	-0.487	-1.181	-0.501	0.47	-0.937	-1.842	-1.473
	[0.218]	[0.222]	[0.245]	[0.201]	[0.364]	[0.196]	[0.625]	[0.287]	[0.919]	[0.525]
grade alter	0.124	-0.006	0.151	0.045	0.015	0.033	-0.042	0.018	0.441	0.249
	[0.128]	[0.212]	[0.114]	[0.101]	[0.132]	[0.122]	[0.237]	[0.13]	[0.481]	[0.28]
grade ego	0.051	-0.319	-0.103	0.111	-0.027	0.169	0.357	0.05	-0.198	0.181
	[0.136]	[0.225]	[0.127]	[0.106]	[0.113]	[0.115]	[0.369]	[0.137]	[0.28]	[0.237]
grade similarity	-0.411	-0.546	-0.195	0.539	1.326	0.435	1.413	-0.101	-1.554	0.272
	[0.424]	[0.418]	[0.447]	[0.427]	[0.636]	[0.392]	[1.045]	[0.39]	[1.113]	[0.67]
TGB alter	0.143	-0.038	0.066	-0.043	0.042	-0.061	-0.363	0.017	0.076	0.05
	[0.133]	[0.071]	[0.063]	[0.057]	[0.078]	[0.072]	[0.223]	[0.069]	[0.154]	[0.147]
TGB ego	0.102	-0.166	0.032	0.096	-0.071	-0.144	0.121	0.085	0.107	0.1
	[0.12]	[0.084]	[0.079]	[0.075]	[0.075]	[0.088]	[0.194]	[0.073]	[0.108]	[0.124]
TGB similarity	0.886	-0.862	0.892	-1.566	-0.883	-0.335	-1.351	0.867	-2.113	0.467
	[1.417]	[0.662]	[0.576]	[0.671]	[0.424]	[0.64]	[1.291]	[0.831]	[1.193]	[0.798]
popularity alter	-0.308	-0.098	-0.16	-0.077	0.027	-0.262	-0.27	-0.045	-0.834	-0.405
	[0.123]	[0.06]	[0.103]	[0.065]	[0.128]	[0.101]	[0.179]	[0.074]	[0.394]	[0.189]
popularity ego	-0.052	-0.009	0.037	0.016	-0.038	-0.011	-0.198	0.082	0.064	-0.106
	[0.065]	[0.048]	[0.189]	[0.053]	[0.095]	[0.237]	[0.142]	[0.101]	[0.151]	[0.113]
popularity ego x popularity alter	-0.026	-0.012	-0.012	-0.03	0.005	0.035	-0.04	-0.018	-0.016	-0.055
	[0.022]	[0.012]	[0.032]	[0.022]	[0.039]	[0.028]	[0.072]	[0.036]	[0.049]	[0.04]
Overall maximum convergence ratio:	0.214	0.157	0.159	0.126	0.134	0.137	0.154	0.161	0.212	0.128

Table 47: Results of the meta-analysis for negative network and TGB, Model 2

	est	se	N	p	tau2	Q	Qp
outdegree (density)	-2.128	0.142	20	0.000	0.000	13.236	0.826
reciprocity	0.591	0.080	20	0.000	0.001	19.624	0.418
transitive triplets	-0.095	0.024	20	0.000	0.040	22.175	0.276
indegree - popularity	0.114	0.014	20	0.000	0.002	14.281	0.767
outdegree - activity	0.098	0.010	20	0.000	0.021	26.164	0.126
indegree - activity	-0.019	0.045	4	0.672	0.000	2.074	0.557
outdegree-trunc(1)	-2.199	1.361	4	0.106	2.091	7.333	0.062
sex alter	0.024	0.077	20	0.756	0.162	36.170	0.010
sex ego	0.178	0.078	19	0.023	0.162	30.996	0.029
same sex	-0.510	0.067	20	0.000	0.117	28.613	0.072
grade alter	0.034	0.035	20	0.333	0.016	17.005	0.590
grade ego	0.029	0.039	20	0.465	0.062	19.492	0.426
grade similarity	-0.045	0.112	20	0.691	0.172	24.937	0.163
TGB alter	-0.014	0.018	20	0.433	0.000	12.943	0.841
TGB ego	0.000	0.036	20	0.998	0.108	40.943	0.002
TGB similarity	-0.295	0.217	20	0.174	0.547	29.831	0.054
popularity alter	-0.147	0.024	20	0.000	0.037	29.148	0.064
popularity ego	-0.015	0.016	20	0.344	0.000	10.104	0.950
popularity ego x popularity alter	-0.016	0.005	20	0.001	0.000	15.893	0.664
int. TGB ego x TGB similarity	0.037	0.066	20	0.577	0.079	19.797	0.407

Table 48: Goodness of fit statistics, Negative & TGB, Model 2

	Indegree distribution	Outdegree distribution	Geodesic Distance	Triad Census
1	0.750	0.547	0.938	0.810
2	1.000	0.901	0.931	0.935
3	0.639	0.954	1.000	0.815
4	0.224	0.280	0.148	0.130
5	0.341	0.096	0.040	0.985
6	0.976	0.157	0.114	1.000
7	0.021	0.763	0.185	0.785
8	0.952	0.641	0.106	0.980
9	0.923	0.420	0.086	0.871
10	0.946	0.075	0.824	0.769
11	0.727	0.792	0.879	0.805
12	0.227	0.478	0.843	0.972
13	0.987	0.088	0.034	0.499
14	0.411	0.246	0.791	0.861
15	0.989	0.131	0.440	0.318
16	0.649	0.620	0.026	0.672
17	0.994	0.839	0.948	1.000
18	0.058	0.250	0.140	0.444
19	0.984	0.748	0.433	0.501
20	0.470	0.562	0.950	0.725

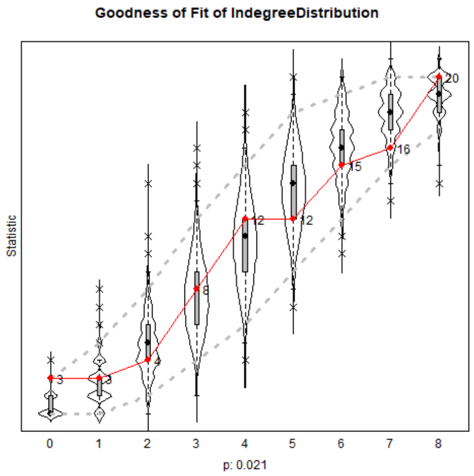

(a) Class 7

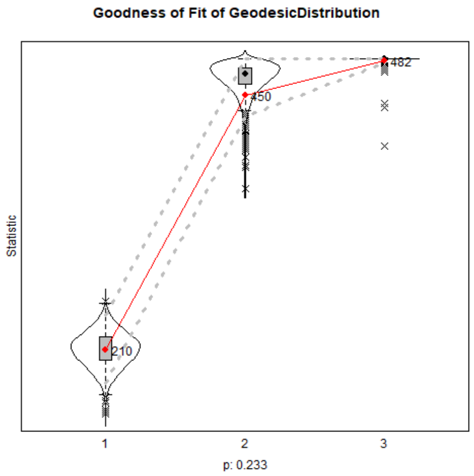

(b) Class 5

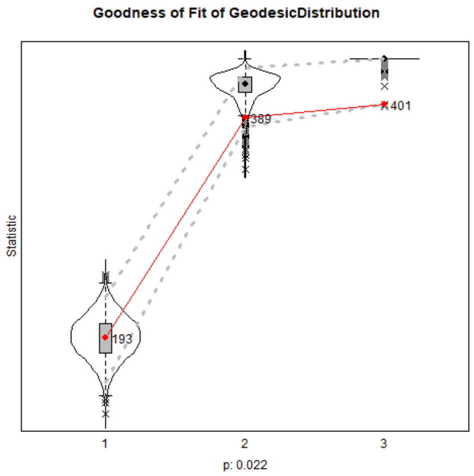

(c) Class 13

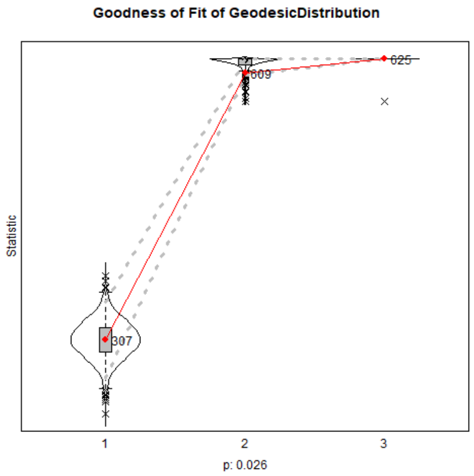

(d) Class 16

Figure 16: Classes with poor fit, Negative & TGB, Model 2

Table 49: Results of separate SAOMs for negative network and TGB, Model 2

	1	2	3	4	5	6	7	8	9	10
outdegree (density)	-1.969	1.26	-0.771	-3.019	-2.409	-0.961	-2.545	-1.936	-1.548	-1.895
	[0.362]	[6.373]	[1.629]	[1.029]	[0.622]	[1.064]	[0.635]	[0.374]	[0.445]	[3.031]
reciprocity	0.652	1.865	0.08	0.233	1.078	0.646	1.243	0.454	0.352	0.045
	[0.22]	[1.222]	[0.469]	[0.622]	[0.299]	[0.413]	[0.388]	[0.214]	[0.275]	[1.339]
transitive triplets	-0.07	1.563	-0.196	0.324	-0.203	0	-0.109	-0.252	0.029	-0.028
	[0.048]	[1.645]	[0.155]	[0.251]	[0.079]	[0.185]	[0.089]	[0.073]	[0.069]	[0.727]
indegree - popularity	0.101	-0.21	-0.251	0.048	0.103	0.134	0.096	0.115	0.023	-1.347
	[0.035]	[0.481]	[0.28]	[0.13]	[0.074]	[0.043]	[0.065]	[0.043]	[0.055]	[1.045]
outdegree - activity	0.075	-1.305	0.154	0.114	0.148	0.03	0.145	0.109	0.03	0.339
	[0.018]	[2.101]	[0.05]	[0.087]	[0.03]	[0.067]	[0.042]	[0.023]	[0.026]	[0.272]
indegree - activity	0	0	0	0	0	-0.197	0	0	0	0
	[NA]	[NA]	[NA]	[NA]	[NA]	[0.152]	[NA]	[NA]	[NA]	[NA]
outdegree-trunc(1)	0	0	0	1.722	0	0	0	-2.027	0	0
	[NA]	[NA]	[NA]	[1.87]	[NA]	[NA]	[NA]	[1.545]	[NA]	[NA]
sex alter	-0.034	-3.898	-1.829	-0.006	-0.492	-0.001	0.04	0.345	0.252	0.121
	[0.156]	[3.079]	[0.769]	[0.486]	[0.393]	[0.235]	[0.227]	[0.193]	[0.23]	[1.746]
sex ego	-0.071	-0.424	-0.555	0.999	-0.387	-0.359	0.228	0.047	0.379	0
	[0.155]	[2.252]	[0.386]	[0.538]	[0.384]	[0.377]	[0.324]	[0.167]	[0.226]	[NA]
same sex	-0.51	-0.558	-0.602	-0.235	-1.436	-0.094	-0.462	-0.659	-0.522	-1.186
	[0.148]	[1.304]	[0.457]	[0.411]	[0.436]	[0.208]	[0.233]	[0.175]	[0.216]	[1.174]
grade alter	0.101	-0.215	0.016	0.607	0.157	0.048	0.364	-0.17	-0.238	0.136
	[0.142]	[0.474]	[0.245]	[0.345]	[0.17]	[0.106]	[0.196]	[0.1]	[0.152]	[0.642]
grade ego	0.104	0.61	-0.018	0.071	0.192	0.084	0.433	-0.184	-0.143	-1.74
	[0.14]	[1.062]	[0.191]	[0.247]	[0.153]	[0.135]	[0.268]	[0.101]	[0.15]	[2.124]
grade similarity	-0.006	-0.563	0.779	0.252	-0.699	-0.583	-0.162	-0.615	0.06	3.54
	[0.248]	[1.693]	[0.525]	[0.916]	[0.938]	[0.467]	[0.36]	[0.29]	[0.303]	[2.102]
TGB alter	0.019	-1.822	0.107	-0.138	0.018	-0.093	-0.116	-0.035	-0.011	-0.04
	[0.039]	[1.834]	[0.133]	[0.178]	[0.061]	[0.086]	[0.111]	[0.045]	[0.094]	[0.38]
TGB ego	-0.013	-1.941	-0.237	-0.126	-0.186	0.273	0.24	0.104	-0.238	0.75
	[0.044]	[2.429]	[0.107]	[0.215]	[0.076]	[0.163]	[0.148]	[0.048]	[0.12]	[0.564]
TGB similarity	0.447	-4.619	-0.752	-4.833	-0.215	0.464	0.575	-0.061	-1.131	-1.249
	[0.404]	[5.106]	[0.959]	[2.537]	[0.62]	[1.06]	[0.53]	[0.457]	[0.92]	[2.901]
popularity alter	-0.103	-0.017	-0.722	-0.576	-0.254	-0.049	-0.36	-0.166	-0.157	-1.72
	[0.053]	[0.426]	[0.378]	[0.242]	[0.105]	[0.073]	[0.122]	[0.058]	[0.088]	[1.001]
popularity ego	0.027	-1.449	-0.151	-0.054	-0.019	-0.144	-0.002	-0.038	-0.053	0.597
	[0.03]	[3.337]	[0.107]	[0.149]	[0.07]	[0.155]	[0.099]	[0.041]	[0.053]	[0.943]
popularity ego x popularity alter	-0.023	0.258	0.023	-0.146	0.028	-0.018	0.016	-0.019	0.005	0.053
	[0.008]	[0.342]	[0.039]	[0.067]	[0.036]	[0.021]	[0.037]	[0.016]	[0.017]	[0.11]
int. TGB ego x TGB similarity	0.101	0.161	-0.455	0.09	-0.453	0.847	0.466	0.092	-0.286	0.991
	[0.155]	[2.741]	[0.292]	[0.367]	[0.253]	[0.52]	[0.485]	[0.15]	[0.216]	[1.293]
Overall maximum convergence ratio:	0.126	0.089	0.190	0.130	0.113	0.180	0.143	0.128	0.130	0.159

	11	12	13	14	15	16	17	18	19	20
outdegree (density)	-1.859	-2.852	-1.596	-2.467	-3.185	-2.241	-2.444	-2.191	-0.886	-1.672
	[0.715]	[0.572]	[0.852]	[0.531]	[0.585]	[0.899]	[0.899]	[0.439]	[1.086]	[0.95]
reciprocity	0.555	0.952	0.798	0.71	-0.218	0.395	0.444	0.741	-1.951	-0.001
	[0.368]	[0.353]	[0.312]	[0.267]	[0.439]	[0.293]	[0.87]	[0.421]	[1.386]	[0.571]
transitive triplets	-0.228	-0.033	-0.012	-0.07	-0.232	-0.098	-0.841	-0.036	-0.78	-0.292
	[0.102]	[0.069]	[0.106]	[0.05]	[0.173]	[0.081]	[0.895]	[0.09]	[0.513]	[0.224]
indegree - popularity	0.105	0.107	0.083	0.129	0.203	0.104	-0.073	0.18	0.003	0.04
	[0.072]	[0.066]	[0.057]	[0.045]	[0.059]	[0.048]	[0.192]	[0.041]	[0.214]	[0.213]
outdegree - activity	0.105	0.089	0.066	0.085	0.184	0.107	0.014	0.068	0.256	0.205
	[0.04]	[0.026]	[0.034]	[0.024]	[0.051]	[0.026]	[0.087]	[0.04]	[0.123]	[0.065]
indegree - activity	0	0	-0.055	0	0	-0.036	0	0.025	0	0
	[NA]	[NA]	[0.107]	[NA]	[NA]	[0.105]	[NA]	[0.06]	[NA]	[NA]
outdegree-trunc(1)	-4.56	0	0	0	0	0	0	0	-3.668	0
	[1.507]	[NA]	[NA]	[NA]	[NA]	[NA]	[NA]	[NA]	[2.087]	[NA]
sex alter	-0.204	-0.318	-0.292	0.152	1.253	0.062	-1.208	0.259	-2.823	-0.642
	[0.242]	[0.288]	[0.323]	[0.197]	[0.376]	[0.219]	[0.828]	[0.207]	[1.511]	[0.504]
sex ego	0.118	0.453	0.082	0.351	0.924	0.414	1.991	0.338	-1.801	-0.19
	[0.258]	[0.282]	[0.284]	[0.209]	[0.331]	[0.229]	[0.893]	[0.235]	[1.062]	[0.41]
same sex	-0.223	-0.177	-0.421	-0.493	-1.146	-0.508	0.476	-0.938	-1.828	-1.506
	[0.221]	[0.211]	[0.254]	[0.199]	[0.342]	[0.205]	[0.633]	[0.287]	[0.774]	[0.554]
grade alter	0.122	-0.037	0.146	0.045	0.016	0.032	-0.029	-0.024	0.376	0.269
	[0.132]	[0.204]	[0.128]	[0.1]	[0.134]	[0.119]	[0.231]	[0.132]	[0.501]	[0.252]
grade ego	0.05	-0.268	-0.105	0.113	-0.035	0.227	0.402	0.134	-0.177	0.19
	[0.133]	[0.213]	[0.119]	[0.106]	[0.115]	[0.138]	[0.373]	[0.151]	[0.279]	[0.23]
grade similarity	-0.417	-0.495	-0.201	0.499	1.414	0.488	1.37	-0.107	-1.635	0.219
	[0.44]	[0.408]	[0.422]	[0.431]	[0.691]	[0.408]	[1.077]	[0.387]	[1.206]	[0.69]
TGB alter	0.137	-0.099	0.066	-0.024	-0.093	-0.033	-0.4	-0.061	0.073	0.082
	[0.126]	[0.084]	[0.073]	[0.059]	[0.115]	[0.083]	[0.237]	[0.094]	[0.156]	[0.171]
TGB ego	0.111	-0.12	0.027	0.093	-0.054	-0.181	0.155	0.212	0.121	0.076
	[0.134]	[0.078]	[0.097]	[0.076]	[0.08]	[0.097]	[0.227]	[0.134]	[0.106]	[0.137]
TGB similarity	0.842	-1.098	0.885	-1.593	-0.931	-0.151	-1.583	0.168	-2.06	0.541
	[1.305]	[0.643]	[0.552]	[0.677]	[0.434]	[0.741]	[1.357]	[1.026]	[0.996]	[0.783]
popularity alter	-0.305	-0.102	-0.154	-0.081	0.044	-0.265	-0.273	-0.025	-0.84	-0.413
	[0.124]	[0.062]	[0.112]	[0.067]	[0.125]	[0.097]	[0.184]	[0.074]	[0.447]	[0.192]
popularity ego	-0.049	-0.004	0.043	0.014	-0.059	-0.035	-0.203	0.065	0.048	-0.115
	[0.067]	[0.046]	[0.17]	[0.054]	[0.099]	[0.209]	[0.141]	[0.106]	[0.137]	[0.111]
popularity ego x popularity alter	-0.027	-0.016	-0.013	-0.03	-0.014	0.038	-0.038	-0.022	-0.011	-0.056
	[0.022]	[0.013]	[0.029]	[0.023]	[0.039]	[0.028]	[0.065]	[0.036]	[0.048]	[0.042]
int. TGB ego x TGB similarity	0.026	0.285	-0.015	-0.349	0.506	-0.192	0.124	0.353	0.181	-0.2
	[0.217]	[0.191]	[0.356]	[0.437]	[0.324]	[0.259]	[0.442]	[0.306]	[0.305]	[0.575]
Overall maximum convergence ratio:	0.183	0.103	0.156	0.138	0.176	0.087	0.171	0.121	0.218	0.113

References

1. Ripley RM, Snijders TA, Boda Z, Vörös A, Preciado P. Manual for RSiena. University of Oxford: Department of Statistics, Nuffield College and University of Groningen: Department of Sociology; 2024.
